# A High-Content Imaging Pipeline to Investigate Subcytotoxic Effects in RTgill-W1 Cells

**DOI:** 10.1021/acs.est.5c18316

**Published:** 2026-07-29

**Authors:** Miha Tome, Barbara Jozef, Sven Lukas Mosimann, Marissa Kosnik, Kristin Schirmer, Anže Županič

**Affiliations:** † Department of Biotechnology and Systems Biology, National Institute of Biology, Ljubljana 1000, Slovenia; ‡ 28499 Environmental Toxicology, Eawag, Dübendorf 8600, Switzerland; ¶ Institute of Biogeochemistry and Pollutant Dynamics, ETHZ, Zürich 8092, Switzerland

**Keywords:** high-content screening, phenotyping, toxicology, fish cells, concentration−response, ecotoxicology

## Abstract

High-content screening offers great potential for in vitro toxicology, yet its application in environmental test systems remains limited by the lack of open, standardized workflows and biologically interpretable analysis frameworks. Here, we established and optimized a reproducible pipeline for image-based phenotypic profiling of rainbow trout gill cells (RTgill-W1) exposed to environmental chemicals, using Tebuthiuron as a case study. The workflow integrates multiple organelle dyes and systematically evaluates critical data processing steps (quality control, standardization, outlier removal, concentration–response-guided feature selection, and dimensionality reduction) to identify robust, biologically meaningful end points. Our systematic comparison demonstrated remarkable robustness: even minimal processing detected concentration–response relationships, while optimized standardization and feature selection substantially improved signal clarity. The framework identified 94 morphological features capturing Tebuthiuron’s concentration- and time-dependent cellular responses, with lysosomal redistribution and mitochondrial fragmentation emerging as early stress indicators. Maximum mean discrepancy and Uniform Manifold Approximation and Projection (UMAP) visualizations provided biologically interpretable maps of phenotypic divergence. This proof-of-concept demonstrates that our quality-controlled, transparent HCS pipeline can resolve concentration- and time-dependent subcellular responses in fish cells, establishing a methodological basis for future multichemical phenotypic profiling.

## Graphical abstract

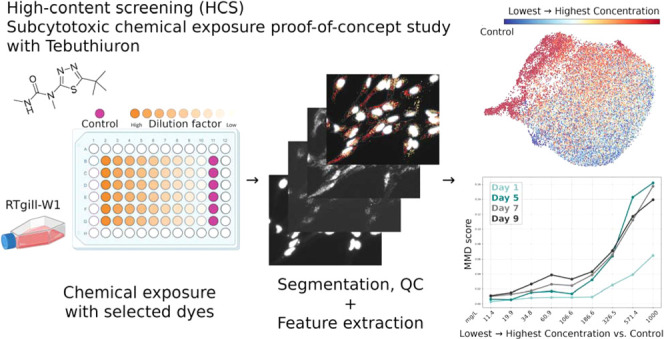

## Introduction

Fish cell lines are foundational tools in aquatic toxicology, offering cost-effective, scalable, and ethical alternatives to whole-organism tests.^1–4^ These in vitro systems have proven effective for toxicity screening of a wide range of chemicals and nanomaterials^5–8^ and researchers increasingly employ them for advanced applications such as genotoxicity assessment, investigation of toxic mechanisms, and biomarker discovery.^4,9–11^ This mirrors a larger trend in toxicology that emphasizes high-throughput, molecular approaches to understand how chemicals exert their effects.^12^ Applying phenotypic profiling approaches to fish cells is vital, as it can uncover a variety of phenotypic reactions, like organelle-specific damage or morphological shifts, that go undetected by conventional viability tests.^13–15^

The RTgill-W1 cell line is an established fish in vitro model for assessing chemical effects on cellular viability, as recognized in OECD Test Guideline 249,^16^ while inhibition of cell population growth has also been shown to predict reduced fish growth in vivo following chemical exposure.^17^ A key unresolved question is whether the common end point of reduced cell proliferation reflects shared or substance-specific cellular mechanisms. Addressing this requires methods capable of capturing early, early subcellular phenotypes before overt cytotoxicity.

High-content screening (HCS) offers distinct advantages over traditional toxicology, providing sensitive, single-cell resolution data at subcytotoxic concentrations.^13,18^ Its untargeted nature facilitates unbiased, evidence-led discovery of mechanistic processes agnostic to mode of action,^19^ opening opportunities for hypothesis-free investigation of perturbation-related effects. As a scalable framework, HCS assays like Cell Painting^20^ are comparatively cost-effective alternatives compared to the majority of omics methods.^19,21^

Despite its power, HCS assays can present significant computational challenges. The high-dimensional, single-cell data requires a complex workflow of quality control, normalization, and feature selection to correct for experimental artifacts and batch effects.^22,23^ Furthermore, standard data aggregation methods like the mean or median often fail to capture important changes in population distributions, necessitating more advanced analytical metrics.^19,24^

While HCS has been extensively developed and applied in mammalian cell systems for drug discovery applications,^25^ its application in ecotoxicology remains largely underdeveloped. Existing fish cell HCS studies have primarily applied Cell Painting-based approaches to RTgill-W1 cells in acute, single-time point settings aimed at benchmarking phenotypic bioactivity against cytotoxic thresholds.^13,26^ The present study takes a complementary direction: rather than broad morphological coverage, we establish a long-term (nine-day), subcytotoxic workflow using a targeted live-cell staining panel, Hoechst 33342 (nuclei), TMRM (mitochondria), and quinacrine (lysosomes), selected for their toxicological relevance to growth-inhibitory end points and compatibility with standard fluorescence imaging equipment. Building on the short-term proliferation assay reported in Jozef et al.^27^ we expand the experimental design to include broad concentration ranges and multiple time points, integrating classical viability readouts with organelle-level phenotypic profiles across the subcytotoxic exposure window. The core contribution is a proof-of-concept framework that integrates experimental choices (targeted live-cell staining, 96-well format, subcytotoxic long-term exposures) with a systematically evaluated analytical workflow (quality control, batch effect correction, concentration–response-guided feature selection, dimensionality reduction, and population-level comparison), with biological interpretation of the resulting phenotypic profiles treated as an integral part of the design.

To demonstrate this framework, we selected the herbicide Tebuthiuron as a model environmental stressor. While exhibiting low acute fish toxicity, Tebuthiuron induces a broad range of sublethal chronic effects across diverse fish species, ranging from reduced growth to oxidative stress and developmental alterations. Additionally, internal (unpublished) data show it arrests cell proliferation in RTgill-W1 cells under chronic exposure. This profile makes it an ideal showcase chemical to establish whether our framework effectively captures concentration- and time-dependent subcellular responses.^28–31^

## Material and Methods

### Materials and Test Chemical

RTgill-W1 rainbow trout gill cell line (CRL-2523 ATCC, Cellosaurus database, reference: RRID: CVCL_6441) was used in this study. Leibovitz’ L-15 medium (L15, P04-27600) and Trypsin (0.25% in PBS, w/o Ca and Mg) were purchased from PAN-Biotech GmbH. Fetal bovine serum (FBS, S182H-500) was purchased from Eurobio Scientific. Cell culture flasks of various sizes (T25, T75) and conical tubes were purchased from Faust. Casy Ton (5651808) and Casy cups were purchased from Omni Life Science GmBh. Versene (11518876), alamarBlue Cell Viability Reagent, Hoechst 33342 (H3570), Tetramethylrhodamine, Methyl Ester, Perchlorate (TMRM), 10× Dulbecco’s phosphate buffer saline (PBS) fortified with Ca^2+^ and Mg^2+^ were purchased from Invitrogen. Quinacrine-dihydrochloride (Q3251) and Tebuthiuron (Sigma 45671, Cas-Nr: 34014-18-1, Purity >98%) were purchased from Sigma-Aldrich.

### Cell Line and Cell Culture

The rainbow trout gill cell line RTgill-W1 was routinely cultured in 75 cm^2^ cell culture flasks in 10 mL L-15 medium supplemented with 5% FBS (v/v) at 19 ± 1 °C under regular atmospheric conditions in an incubator in the dark (referred to as standard conditions). Confluent cells were washed twice with Versene (Invitrogen) followed by detachment with trypsin (0.25% in phosphate-buffer saline without calcium and magnesium; Biowest) and resuspended in the L-15/FBS medium, applying a cell splitting ratio of 1:2, every 2 weeks. Cells obtained in suspension were counted via Casy TTC Cell Counter & Analyzer System according to the manufacturer’s instructions (Schärfe System). RTgill-W1 cells with different passage numbers (from P-54 to P-95) were used for the experiments. Mycoplasma tests (MycoAlert PLUS Mycoplasma Detection Kit, Lonza) were carried out every three months during this study, confirming absence of any contamination.

### Cell Seeding for HCS Experiments

We used the same plate layout as described by Jozef et al.^27^ and showcased in Figure 1. In brief, cells were seeded in a laminar flow hood into each of the inner 60 wells of a 96-well plate (Greiner Bio-One black μClear) at a density of 7500 cells per well (∼2.34 × 10^4^ cells/cm^2^) in 200 μL of cell culture medium (L15, supplemented with 5% FBS). After seeding, plates were placed in the incubator. Chemical exposure started 24 h after cell seeding. For each of the exposure time points, we prepared matched sets of 96-well plates from the same cell suspension. In total, eight plates were seeded: for each time point, one plate was dedicated to cell viability assays, and the corresponding plate was used for high-content imaging.

**Figure 1:**
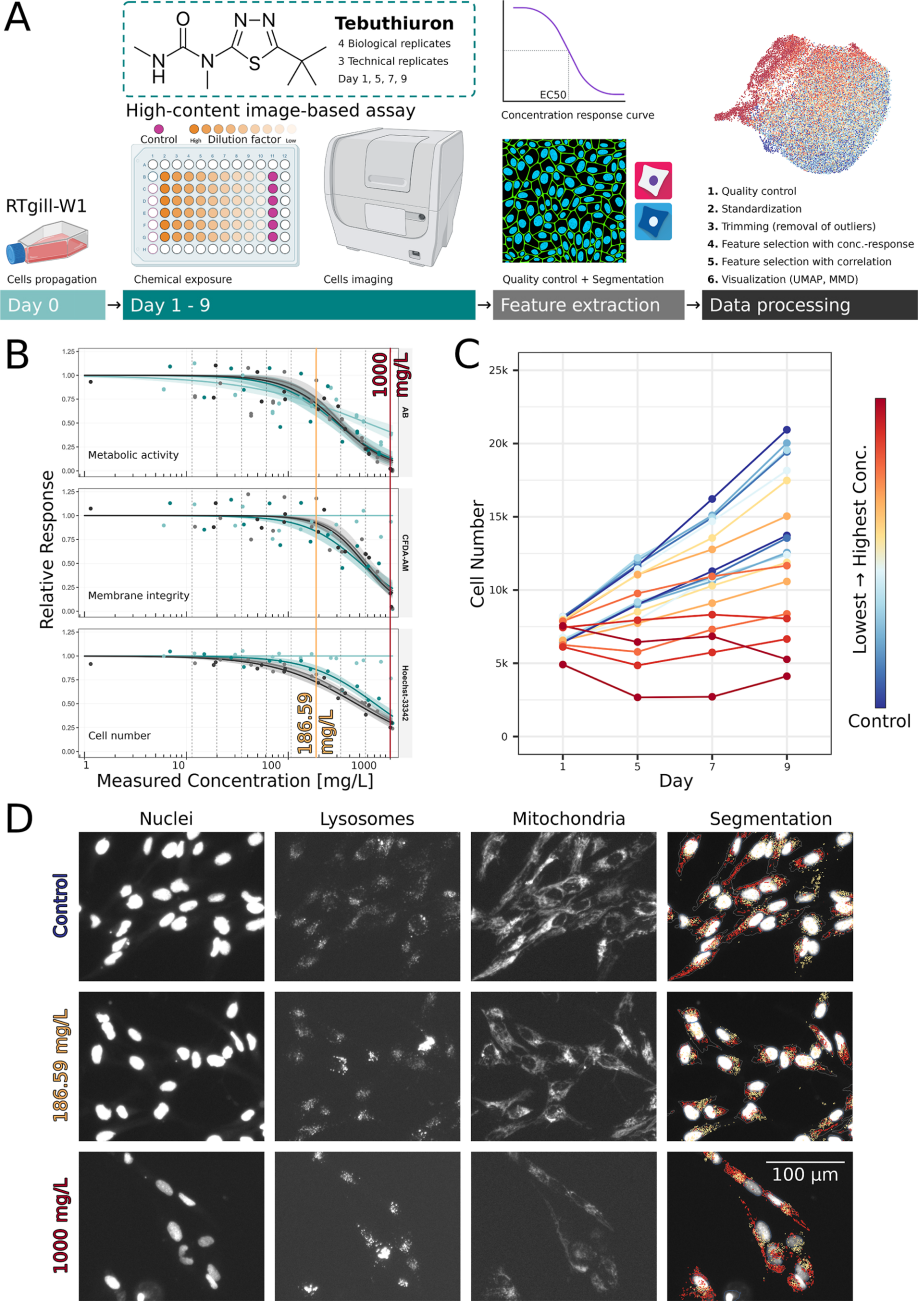
High-content bioimage phenotyping pipeline and viability assessment. (A) Experimental workflow showing RTgill-W1 cells seeded in 96-well plates and exposed to nine concentrations of Tebuthiuron across multiple time points (days 1, 5, 7, and 9). Cells were stained with fluorescent dyes (Hoechst 33342 for nuclei, TMRM for mitochondria, quinacrine for lysosomes) and imaged using Agilent Cytation 5. Images were processed through CellProfiler for segmentation and feature extraction, followed by a six-step computational pipeline: quality control, standardization, outlier removal, concentration–response feature selection, correlation-based feature selection, and dimensionality reduction. (B) Concentration–response curves for metabolic activity (AB), membrane integrity (CFDA-AM), and cell number (Hoechst 33342) across exposure time points. Horizontal dotted lines correspond to the nominal concentrations, whereas points were plotted using the measured values. (C) Growth curves showing cell counts at each concentration over time. (D) Representative fluorescence images and segmentation of control cells and cells exposed to Tebuthiuron for 5 days, showing nuclei (blue), lysosomes (green/yellow), and mitochondria (red). Building on the 96-well short-term cell proliferation assay reported in Jozef et al.,^27^ we further adapted the design here to enable extended sampling at days 1, 5, 7, and 9. This modification allowed us to capture both short- (≤5 days of exposure) and long-term responses (>5 days), and to integrate cell viability measures with multiplex organelle imaging in a unified workflow, providing an essential viability-anchored reference point for the phenotypic analysis. In mammalian cell lines, multiplex imaging of mitochondria and lysosomes (e.g., via related dyes) has been used to detect early drug-induced dysfunction before cell death.^43,44^ Our study translates a similar concept to fish cells, showing that mitochondria and lysosomes show stress at concentrations before overt membrane damage or cell loss.

### Chemical Exposure and Plate Layout

The plates containing serial dilutions of Tebuthiuron and no-chemical (negative) control wells were prepared by combining FBS-containing medium (L-15/5% FBS) and various concentrations of the chemical as follows. An appropriate amount corresponding to the highest used concentration (1000 mg/L), was weighed-in and dissolved in medium. Following three cycles of 20 min sonication and 20 min stirring (150 rpm), the solution was sterile filtered, and 9 exposure solutions ranging from 1000 to 11.36 mg/L were prepared under sterile conditions. Next, a 2.2 mL deep-well plate (Huberlab, Aesch, Switzerland), mirroring the 96-well plate layout (Figure 1), was prepared, and cells were exposed by transferring 200 μL from the corresponding deep-well plate. In addition, a negative control consisting of pure cell culture medium was added to one column and pure medium was added to the outer wells in order to reduce evaporation. Plates were then sealed with adhesion film and incubated under standard cell culture conditions. Cells were exposed for day 1, 5, 7, and 9 before processing. Medium concentrations were analytically confirmed to be as close to nominal across two independent biological replicates (detailed in Supporting Information—Section S7); therefore, concentrations are presented as nominal for all subsequent analyses.

### Concentration Response Curves

After 1, 5, 7, and 9 days of exposure, concentration response curves for cell viability and cell number were obtained. For this purpose, the cells were washed with PBS supplemented with Ca^2+^ and Mg^2+^ (Biowest, Nuaillé, France) and measured on a plate imaging reader (BioTek Cytation 5 Cell Imaging Multimode Reader, Agilent, Santa Clara, CA, USA). While the upper 3 rows were used for the assessment of metabolic activity and membrane integrity, using alamarBlue (ThermoFisher Scientific, Waltham, MA, USA) and CFDA-AM (ThermoFisher Scientific, Waltham, MA, USA), respectively, the lower 3 were used for cell counting via nuclear staining with Hoechst-33342 (ThermoFisher Scientific, Waltham, MA, USA). Metabolic activity and membrane integrity data were normalized following, and cell numbers were directly normalized to the unexposed control. Two-parameter concentration response curves (upper limit set to 1, lower to zero) were fitted, using the R (version 4.5.1) package drc^32^ and visualized using ggplot2.^33^

### High-Content Image-Based Assay Using Multiplexed Fluorescent Dyes

Live-cell imaging was performed using a multiplexed staining approach to visualize nuclei, mitochondria, and lysosomes in RTgill-W1 cells. The details of cytological markers, their cellular targets and suppliers are described in Table 1. TMRM stock (10 mM) was prepared in DMSO and diluted to a 100 μM intermediate solution in L-15. Quinacrine stock (5 mM) was prepared by dissolving the powder directly in L-15. Hoechst 33342 was prepared as an intermediate solution in L-15.

**Table 1:** Overview of Fluorescent Dyes Used for Multiplex Live-Cell Imaging of Organelle Function in RTgill-W1 Cells

cellular target	dye	stock solution	final exposure concentration	supplier/catalogue number	storage
nucleus	Hoechst 33342	16.2 mM water	9.2 μM in L-15	Invitrogen/H3570	2–8 °C
mitochondria	TMRM	10 mM in DMSO	80 nM in L-15	Invitrogen/T668	–20 °C
lysosomes	Quinacrin	5 mM in L-15	5 μM in L-15	Sigma/Q3251	2–8 °C

For staining, TMRM and quinacrine were diluted in L-15 (without FBS) to generate the labeling solution. From each well, 190 μL of exposure or culture medium was removed and replaced with 100 μL of the TMRM/quinacrine solution, followed by a 30 min incubation. Subsequently, 6 μL of the Hoechst intermediate solution was added directly to each well and cells were incubated for an additional 30 min. The staining solution was then removed, cells were washed three times with 100 μL L-15, and wells were left in the final wash. Plates were immediately transferred to the Cytation 5 (Agilent) for image acquisition.

A total of 270 images (16 bit) were acquired using three different excitation/emission wavelengths: 365/447 nm (nuclei), 465/525 nm (lysosomes), 623/647 nm (mitochondria). Exposure time and laser power setting were optimized using nonexposed cells, while the autofocus was trained based on the center position of the nonexposed well. Images were acquired at 20× objective in wide-field of view mode. The focus for each channel was optimized by examining nonchemical control wells. Typically, 9 unique fields per view were acquired for each well. No postacquisition image processing features of the Cytation 5 software (e.g., filtering, deconvolution, or other image enhancement tools) were applied. Raw images were used directly for analysis.

### Image Analysis

Automated image analysis was performed using CellProfiler 4.2.6 in headless mode.^34^ Quality control (QC) metrics were first extracted from each image using the MeasureImageQuality module. These metrics were subsequently used to train supervised machine learning classifiers in CellProfiler Analyst 3.0.4 ^35^ with Scikit-learn 1.3.2 ^36^ to identify and remove problematic images, including those with artifacts, oversaturation, or empty images.^37^ Classifiers were developed using Random Forest algorithms on a representative subset of images, which were picked manually. Classifier performance was evaluated on the full data set of 4806 images (detailed in Supporting Information—Section S4). Following QC, the remaining images underwent segmentation and feature extraction in CellProfiler. Nuclei stained with Hoechst 33342 were identified as primary objects. Cytoplasmic boundaries were defined by combining adjacent mitochondrial (TMRE) and lysosomal (Quinacrine) fluorescence signals to the primary objects concerning the shape of neighboring cells. Segmentation parameters were manually optimized through visual inspection. Fluorescence quantification was performed for each cell (which was defined as the combined area of nuclei and cytoplasm), mitochondria, lysosomes and nuclei. A total of 781 morphological features per cell were extracted, including area, texture, shape, granularity, signal colocalization, and intensity measurements (detailed in Supporting Information—Section S11). Cell-level data aggregation was performed by calculating the median value of each feature per cell, with total mitochondrial and lysosomal counts per cell appended to each cell’s profile.

### Data Standardization

To account for nonrandomized sample placement on 96-well plates, data were standardized using a two-step approach. Row effects were first corrected by calculating residuals for each feature by subtracting each measurement from its row median (a technical replica). Plate batch effects were subsequently mitigated by subtracting the median residual value across all four biological replicate plates from each individual residual. This two-step standardization produced unitless scores that were used for all downstream analyses. Standard *Z*-score normalization was performed for comparison (Supporting Information—Section S5). To address image variability and segmentation artifacts, outliers were removed by applying 2.5% cutoffs at both distribution tails for each feature, per well.

### Feature Selection

Feature selection was performed through multiple filtering steps. Before data aggregation and standardization, illogical features were removed, including compartment measurements from inappropriate fluorescent channels (e.g., lysosomal measurements from nuclear signal) and highly correlated Haralick texture features.

Following standardization, earth mover’s distance (EMD) scores were calculated for all possible pairwise combinations between control and treatment conditions for all biological replicates. For each feature, concentration–response relationships (from lowest to highest exposure concentration) were evaluated by fitting multiple models to EMD scores: Brain-Cousens modified log–logistic (4- and 5-parameter), constant, linear, four-parameter log–logistic, and Weibull models. Model performance was assessed using Akaike information criterion (AIC) and Bayesian information criterion (BIC). Features were retained for downstream analysis only if they met these conditions across all measurement days: (1) did not exhibit negative slope in linear concentration–response fit (indicating that the highest exposed cells are the most similar to controls), and (2) were not best fitted by a constant model (indicating no concentration–response). The highest exposure concentration was excluded from model fitting to ensure concentration–response detection occurred within subcytotoxic exposure levels. Features with more than 30% missing data were excluded (4 features were removed after correlation filtering step).

For comparison, an alternative feature selection approach following Pearson et al.^24^ was applied, using EMD scores to filter features with high variability among biological replicates in control populations (all above upper inner quartile threshold were removed) and features with very low activity between treated and control populations (Supporting Information—Section S8).

The resulting feature set (182 features) underwent final filtering to remove redundant features. Features were hierarchically clustered (median linkage) based on Pearson correlation, with groups exhibiting *r* ≥ 0.9 considered highly redundant. All features within a group captured essentially identical biological information, differing only in minor computational details (e.g., mean vs median statistics). From each cluster, we retained a single representative feature using a consistent, reproducible rule based on the hierarchical clustering dendrogram structure (Supporting Information—Section S12), removing a total of 82 redundant features.

### Dimensionality Reduction and Population Comparison

Two approaches were employed for dimensionality reduction and population comparison. Maximum mean discrepancy (MMD)^38^ was calculated for pairwise comparisons between each exposure concentration and control across all time points (days 1, 5, 7, 9), with higher values indicating greater morphological divergence from control populations. UMAP^39^ visualization provided qualitative assessment of data transformation and processing steps.

Both analyses used subsampled data sets with equal cell numbers across all concentrations for each day. Missing values were imputed using median values, and data for UMAP analysis was additionally scaled using Quantile Transformer. Alternative distance metrics, including EMD and hybrid Mahalanobis distance,^40^ were calculated on the same subsampled data sets for comparison. For the Mahalanobis distance, the untreated control population alone represented the baseline covariance structure to calculate how far each treated population deviated from this baseline.

## Results and Discussion

Our primary goal was to design and optimize an experimental and computational workflow that enhances concentration–response signal clarity and phenotypic interpretability from large-scale bioimage data sets. We established this proof-of-concept HCS pipeline using Tebuthiuron as a model compound to evaluate chronic and sublethal phenotypic responses; accordingly, the results reflect a compound-specific phenotypic profile in RTgill-W1 cells, with generalizability across structural classes and modes of action to be established through future application to a broader chemical library. The imaging-based end points capture effects with mechanistic underpinnings but do not directly resolve molecular mechanisms, and the targeted live-cell dye panel, prioritizing lysosomal and mitochondrial resolution across multiple exposure time points, represents a biologically motivated adaptation of the morphological profiling concept envisioned in the Cell Painting protocol,^20^ serving a complementary role to broader untargeted approaches. Application to new compounds will probably require revalidation of workflow parameters, including dye selection, QC settings, and exposure duration, but the analytical framework is designed to accommodate these adaptations as the chemical scope expands.

### Setting the Stage

Our experimental design began by adapting a short-term cell proliferation assay^27^ for use in long-term (chronic) exposure experiments. We generated concentration–response data for Tebuthiuron in RTgill-W1 cells using the plate reader functionality of the Cytation 5 instrument for viability measurements. Two complementary nonimaging readouts were employed: alamarBlue to assess cell metabolic activity, and CFDA-AM to evaluate plasma membrane integrity. Cell proliferation was assessed separately by image-based whole-well nuclei counting following Hoechst 33342 staining in imaging mode, using nuclear counts as a proxy for cell number. A nine-point dilution series of Tebuthiuron was tested alongside appropriate controls, including a no-chemical control and a no-cell control for both plate reader viability assays. The same plate layout was used across experiments, allowing the cell population to be followed over extended exposures (days 1, 5, 7, and 9) and providing a window into the time-dependent cellular response. In parallel, the incorporation of a multiplex cell organelle staining, which ultimately expanded the information space, enabled integration of proliferation and organelle-specific stress responses. For every well, a 3 × 3 image tile (no overlap) was acquired using a 20× wide-field of view objective in each fluorescent channel, resulting in 27 images per well (each image ∼690 × 690 μm). Given nine concentrations and three technical replicates, a total of 270 images were captured per biological replicate and time point. Altogether, this experimental design (Figure 1A) was merged into a high-content bioimage phenotyping pipeline. The final steps incorporated a tailor-made image-based cellular analysis converging with the concentration–response curve data, ultimately leading to an integrated workflow that links organelle-level phenotypes with classical viability and cell number readouts for quantitative and mechanistically informed toxicity assessment.

As shown in Figure 1B, all three measures exhibited a sigmoidal concentration–response relationship, with EC_50_ values shifting over time (Supporting Information—Table S7). The tested concentrations were chosen following preliminary range-finding experiments aimed at identifying a concentration range that elicits measurable cellular responses while ensuring adequate cell viability for high-content imaging analysis. These concentrations span a broad concentration–response spectrum, from no observable effect to clearly detectable sublethal effects. The selection was not based on environmental exposure levels but focused on establishing concentration–response relationships under controlled in vitro conditions. In the case of our model chemical Tebuthiuron, the most sensitive indicator was metabolic activity (measured by alamarBlue), which decreased within 24 h post exposure, followed by a significant loss of plasma membrane integrity (CFDA-AM) by day 5, and culminating in reduced cell number at later time points (Table S2). Figure 1C provides further insight into the cellular proliferation of the RTgill-W1 cells under Tebuthiuron exposure over time. The cell number increased steadily for the unexposed cells (control) and the lower chemical concentrations (<200 mg/L), across 9 days, consistent with continued proliferation. In contrast, cells exposed to high concentrations (>750 mg/L) showed plateaued growth curves, indicating arrested cellular proliferation. Intermediate concentrations showed partial cell proliferation in the earliest time points with strong suppression at the later time points. To capture more organelle-specific stress responses, we applied multiplexed biomarker dyes targeting key cellular compartments: Hoechst 33342 (nuclei), TMRM (mitochondria), and quinacrine (lysosomes), to our knowledge, the first such combination reported for phenotypic profiling in fish cells. This combination was selected as a minimal multiplex design, prioritizing accessibility and reproducibility across laboratory settings while targeting the cellular compartments most relevant to antiproliferative and cytotoxic outcomes. Together, nuclear count, mitochondrial membrane potential and morphology, and lysosomal positioning cover the mitochondrial–lysosomal axis, cellular compartments with established relevance to early stress responses related to proliferative and cytotoxic outcomes in fish cells. This complements existing RTgill-W1 HCS studies based on the Cell Painting approach;^13,26^ unlike fixed-cell protocols, the targeted live-cell panel used here prioritizes organelle-specific resolution and concentration–response sensitivity across multiple time points. The trade-off of this minimalistic strategy is a reduced capacity for broad mechanistic inference, as cellular compartments and pathways beyond those targeted may contribute to toxicological outcomes not captured by the selected markers. As shown in Figure 1D, multiplex imaging revealed progressive and organelle-specific perturbations across Tebuthiuron concentrations. Under control conditions, nuclei were evenly distributed and intact, lysosomes appeared numerous and punctate, and mitochondria displayed an extended filamentous network, consistent with healthy proliferating cells. At 186.5 mg/L, nuclei showed irregular morphology, lysosomes appeared enlarged and more clustered, and mitochondria began to lose their organization, indicating early signs of cellular stress. At the highest concentration (1000 mg/L), pronounced nuclear shrinkage and condensation were evident, lysosomes were fewer and displayed abnormal aggregation, and mitochondria appeared highly fragmented and diminished in intensity, and overall cell numbers were substantially reduced. This pattern suggests that Tebuthiuron exposure triggers cellular level stress responses detectable at intermediate concentrations, particularly lysosomal and mitochondrial alterations, before culminating in widespread nuclear damage and cell detachment at high concentrations.

### Pipeline Optimization Strengthens Concentration–Response Signal

Acquisition of images led to feature extraction and data processing (Figure 1A). Raw imaging data was systematically processed in CellProfiler^34^ to achieve robust segmentation of all relevant cellular compartments. Sample output of our complete data processing pipeline is presented in Figure 2. Day 5 was selected due to its strongest concentration response and the greatest morphological divergence from day 1 (as quantified in Figure 2C). We used Uniform Manifold Approximation and Projection (UMAP) for dimensionality reduction because of its ability to preserve both the local and global structure of high-dimensional data.^39^ The UMAP visualization revealed a clear concentration-dependent separation of cell populations, where each point represents an individual cell (Figure 2A and Supporting Information—Section S1). Cells at the highest exposure concentration aggregate in a distinct region from the control cells, indicating that our pipeline successfully captured distinct morphological phenotypes. Mapping individual feature values onto the UMAP embedding enabled feature-specific visualization and biological interpretation (Figure 2B). This approach allowed us to identify which morphological features characterized specific cell populations and their responses to the chemical exposure.

**Figure 2:**
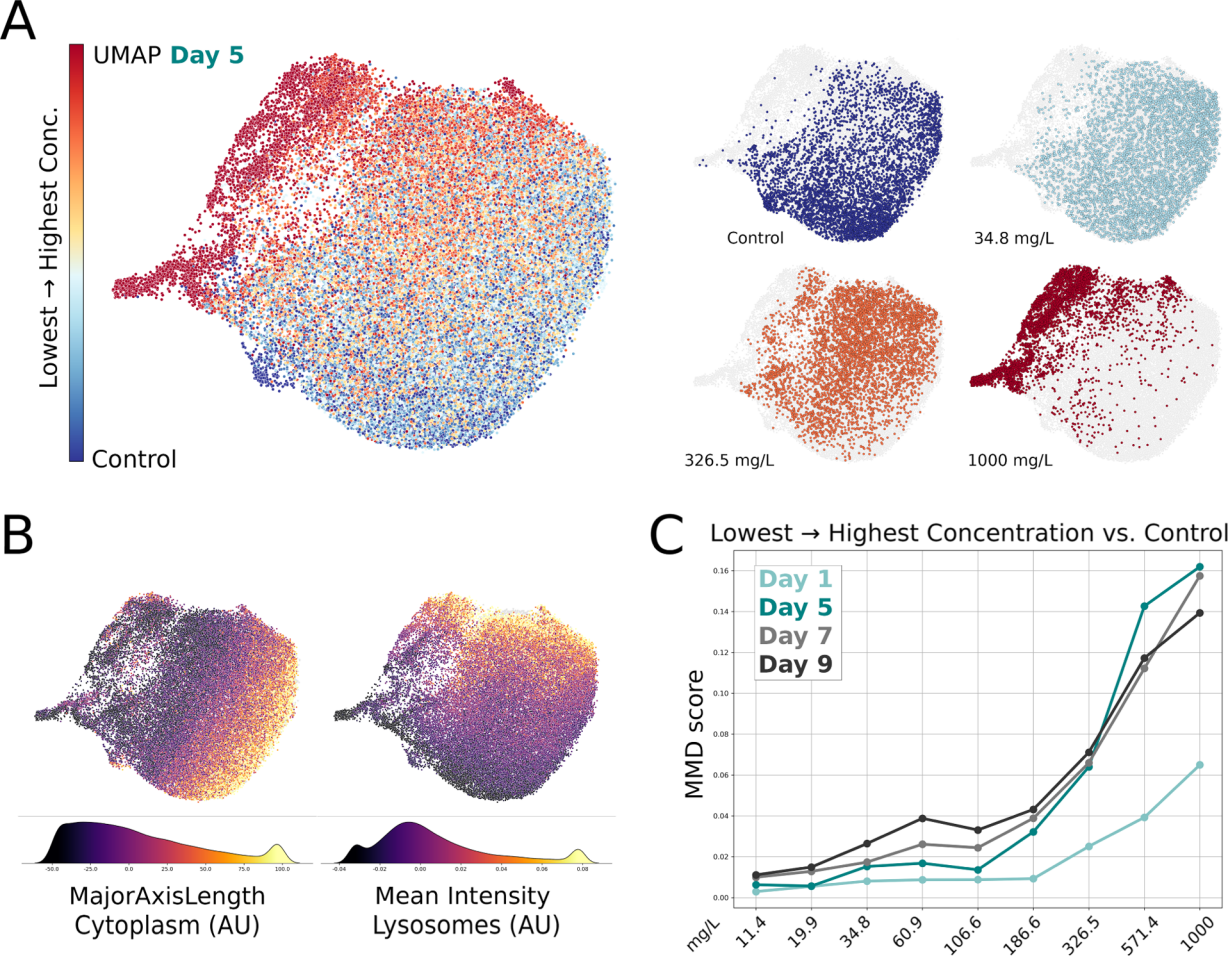
Concentration–response visualization at day 5 and population heterogeneity analysis. (A) UMAP dimensionality reduction of single-cell morphological profiles colored by Tebuthiuron concentration, showing concentration-dependent clustering in the 2D embedding space. (B) The same UMAP embedding colored by representative morphological features (MajorAxisLength cytoplasm and mean intensity lysosomes) showing spatial distribution of feature values across the phenotypic space. (C) Maximum mean discrepancy (MMD) scores quantifying morphological divergence between each exposure concentration and control populations across all time points (days 1, 5, 7, 9).

To quantitatively measure the differences between entire cell populations, we applied the maximum mean discrepancy (MMD) metric.^38^ In essence, MMD quantifies how different two populations are by comparing the average of a kernel function applied across all pairwise combinations of cells from each group; a value of zero indicates identical distributions, while larger values reflect greater dissimilarity. We chose MMD because it is a powerful and robust method for comparing complex, high-dimensional distributions without making any assumptions about their underlying shape. The MMD analysis quantified the concentration–response relationship, with values progressively increasing across the concentration gradient (Figure 2C). Higher MMD values indicated greater morphological divergence from control populations. The analysis captured both concentration-dependent and temporal responses; for example, cells exposed for 5 days showed greater divergence than those exposed for 1 day at equivalent concentrations, demonstrating the sensitivity of our approach to time-dependent effects as well.

#### Data Processing Pipeline Summary

After evaluating and optimizing each step, the final data processing pipeline comprised six steps: (1) quality control, including supervised machine learning image filtering that removed 112 (out of 4086) images containing artifacts, oversaturation, or empty fields, and removal of illogical features (for example, measuring mitochondrial signal in the nuclear channel); (2) correction of row and batch effects using two-step row and plate standardization; (3) removal of outliers beyond the 2.5th and 97.5th percentiles to address segmentation artifacts; (4) selection of concentration-responsive features through model fitting; (5) removal of redundant features with Pearson correlations ≥0.9, at the end retaining 94 out of the initial 781 features per cell; and (6) dimensionality reduction and population-level comparison using UMAP and MMD. This systematic approach successfully extracted concentration–response signals from complex morphological data while preserving temporal dynamics, enabling phenotypic characterization of cellular responses to subcytotoxic long-term chemical exposure.

### Impact of Quality Control

#### Image-Level Quality Control

Visual inspection of the raw image data set revealed three categories of errors common in HCS experiments: artifacts from foreign objects or plate movement, oversaturated fluorescent signals, and empty images. To address the scale of the data set, we employed supervised machine learning classifiers in CellProfiler Analyst^45^ using Random Forest algorithms trained on manually annotated image subsets. Three separate classifiers identified problematic images: artifacts (1.3% detection rate), oversaturation (1.1%), and empty fields (1.4%) (Supporting Information—Section S3). We prioritized stringent selection criteria, accepting false positives over false negatives. The combined classifiers removed 112 out of 4806 images (2.33%). Despite this quality control (QC) step, the impact remained minimal, the concentration–response signal was completely preserved even when omitting this step, demonstrating the robustness of the analytical approach (Figure 3).

**Figure 3:**
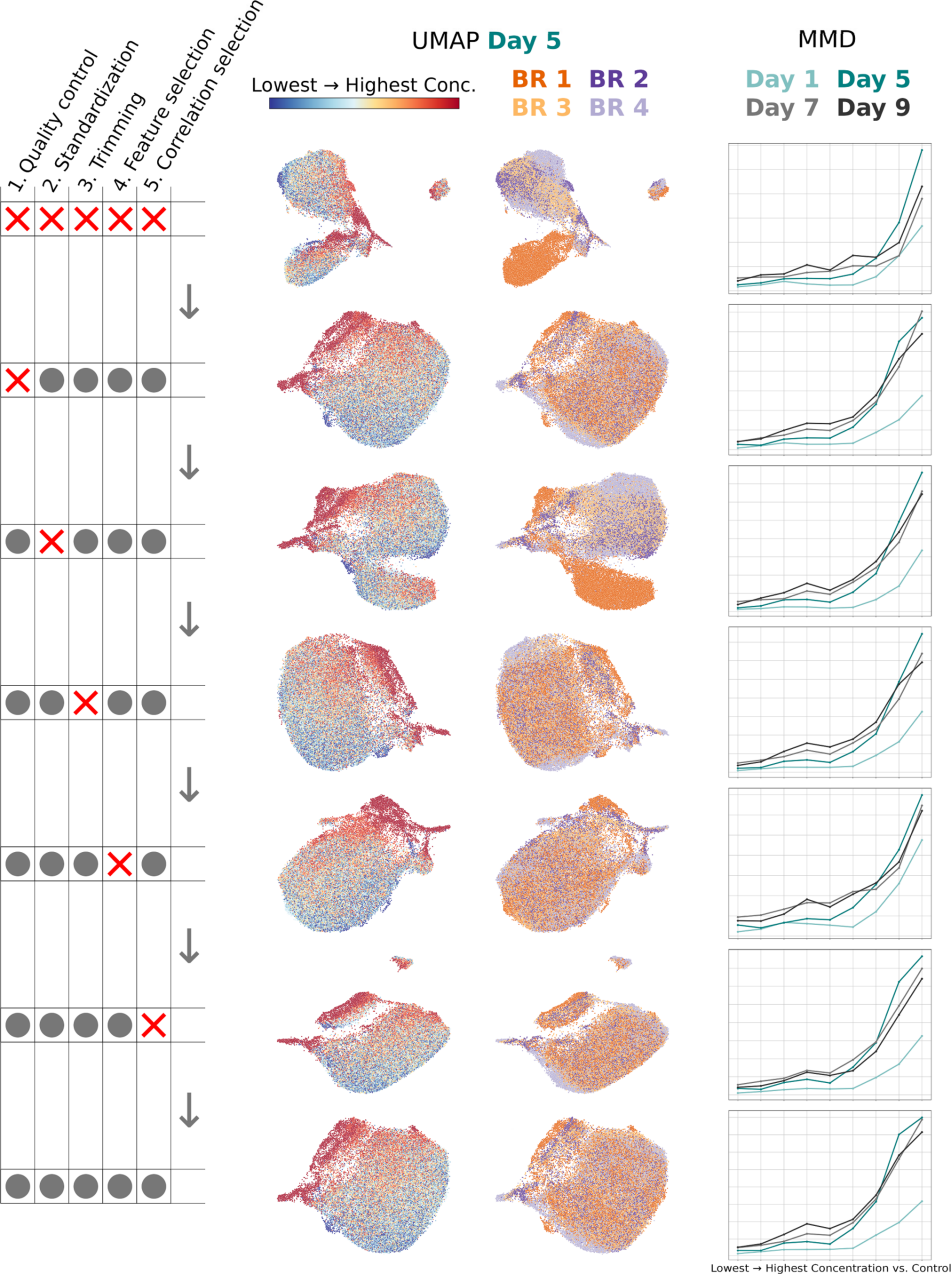
Systematic evaluation of data processing pipeline steps. Left panel: matrix indicating which processing steps are included or omitted (red **X**) in each analysis variant. Middle panels: UMAP embeddings of day 5 data colored by exposure concentration (left) and biological replicate (right), showing how pipeline modifications affect concentration-dependent separation and batch effects. Right panel: MMD scores across all time points (days 1, 5, 7, 9) for each pipeline variant, quantifying the impact of processing steps on concentration–response signal detection.

#### Cell-Level Quality Control

Following feature extraction and standardization, we addressed outliers arising from segmentation artifacts. Existing outlier detection methods that assume feature normality are poorly suited for morphological profiling, as heterogeneous cell populations exhibit diverse phenotypic distributions.^23^ Our data showed non-normal distributions across multiple features (Supporting Information—Section S2), and visual inspection revealed some incorrectly segmented cells producing extreme values (Supporting Information—Section S4). Therefore, we applied well-level outlier removal using 2.5% cutoffs at both distribution tails for each feature, per well. While untrimmed data still showed clear concentration–response trends, outlier removal marginally improved signal clarity, the MMD plots revealed better temporal separation between exposure groups at higher concentrations (Figure 3).

Although QC had minimal impact on the concentration–response signal strength, we still recommend these steps as standard practice, as technical artifacts can mask biological signals in ways that are not always apparent from the data alone.

### Standardization Mitigates Batch Effects

Batch effects severely limit efforts to integrate and interpret image-based profiling data, masking true biological signals and potentially leading to incorrect conclusions.^46^ Our experimental design used fixed well positions for each concentration across plates with the entire row serving as technical replicates and entire plates as biological replicates. This structure precluded the use of standard two-way median polish approaches.^23^ Instead, we implemented a two-step standardization strategy. First, row effects were mitigated by calculating residuals for each feature by subtracting each measurement with its row median (standardization on the technical replica level). Second, batch effects were addressed by subtracting the median residual across all four biological replicate plates from individual residuals (biological replicate level). This approach produced unitless values suitable for downstream analysis.

The effectiveness of batch correction was demonstrated through UMAP visualization, where unstandardized data showed extreme clustering by biological replicate, particularly for BR 1, while standardized data displayed concentration-dependent rather than batch-dependent separation (Figure 3). Consistent with Arevalo et al.,^46^ we found UMAP to be a valuable qualitative tool for assessing batch correction effectiveness, despite its known limitations.^47^ As a comparison *Z*-score normalization yielded comparable results, with similar UMAP separation but marginally worse temporal separation of the MMD scores at lower exposure concentrations (Supporting Information—Section S5).

### Hunting for Concentration–Response

Feature selection is critical in HCS to reduce nonuseful or redundant information and render complex data sets more meaningful, improving both efficiency and accuracy of analysis without losing essential biological information.^23,48^ We initially applied the feature selection methodology from Pearson et al.,^24^ which uses earth mover’s distance (EMD) scores to filter features based on control population variability and treatment response magnitude. EMD measures the minimum amount of work required to transform one feature distribution into another, where work is defined as the product of the amount of probability mass moved and the distance it is shifted; larger values indicate greater distributional divergence between exposed and control populations. However, this approach eliminated features that exhibited concentration–response relationships despite high control variability or low activity. To retain these biologically relevant features, we developed a novel concentration–response-based selection method.

#### Design of Concentration–Response Feature Selection

For each extracted feature, we fitted multiple concentration–response models to EMD scores between exposed and control cell populations. Model performance was assessed using Akaike information criterion (AIC) and Bayesian information criterion (BIC). Features were retained only if they exhibited positive concentration–response relationships (excluding negative linear slopes or constant models) across any measurement days (Figure 4 and Supporting Information—Section S6). This approach prioritized features showing clear concentration-dependent morphological changes relevant to toxicological assessment of the observed phenotype of reduced fish cell proliferation due to chemical exposure (Figure 5 and Supporting Information—Section S7).

**Figure 4:**
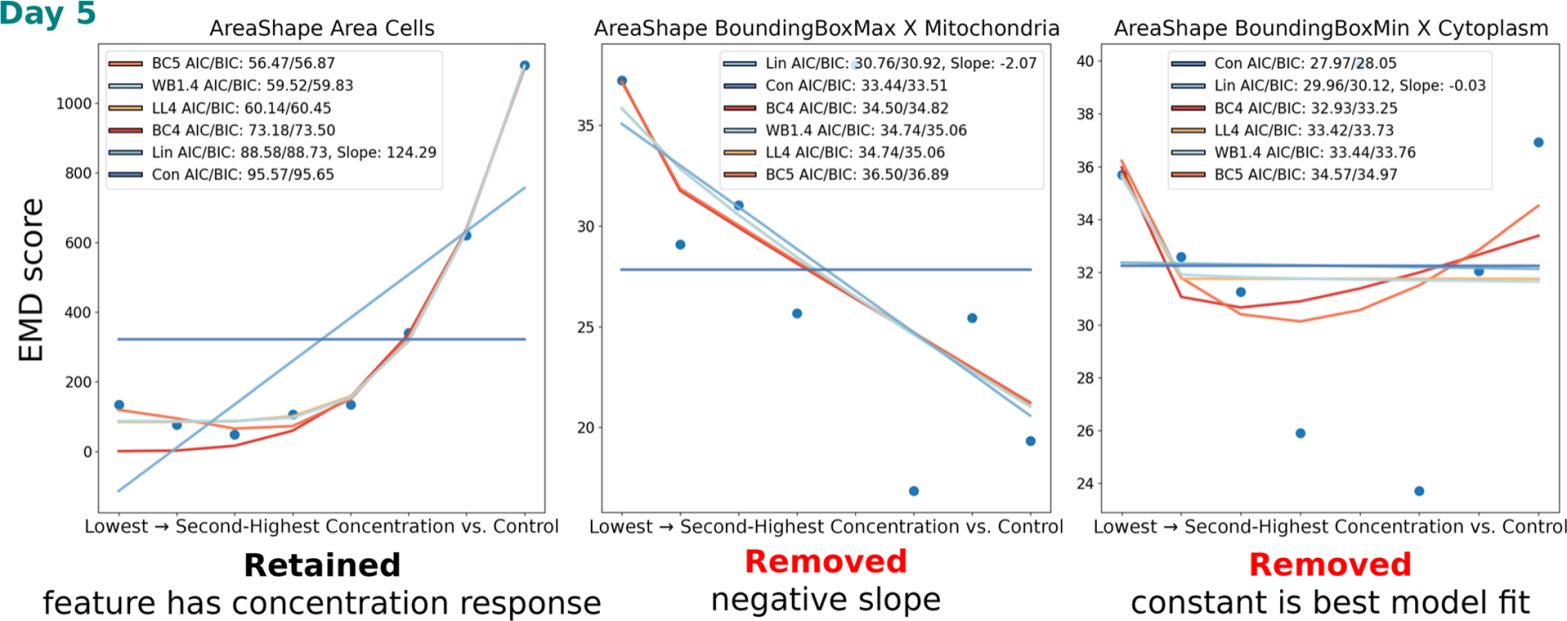
Concentration–response feature selection methodology. Conceptual framework for selecting morphologically relevant features. For each extracted feature, multiple concentration–response models (**BC4/BC5**—Brain-Cousens modified log–logistic model 4/5, **Con**—constant, **Lin**—linear concentration–response, **LL4**—four-parameter log–logistic function, **WB1.4**—Weibull model) were fitted to earth mover’s distance (EMD) scores between exposed and control cell populations. EMD quantifies morphological divergence, with higher values indicating greater population-level differences. Features were retained based on model fit criteria and positive concentration–response relationships. The *X*-axis shows exposure concentrations (lowest to second highest), and the *Y*-axis shows corresponding EMD scores.

**Figure 5:**
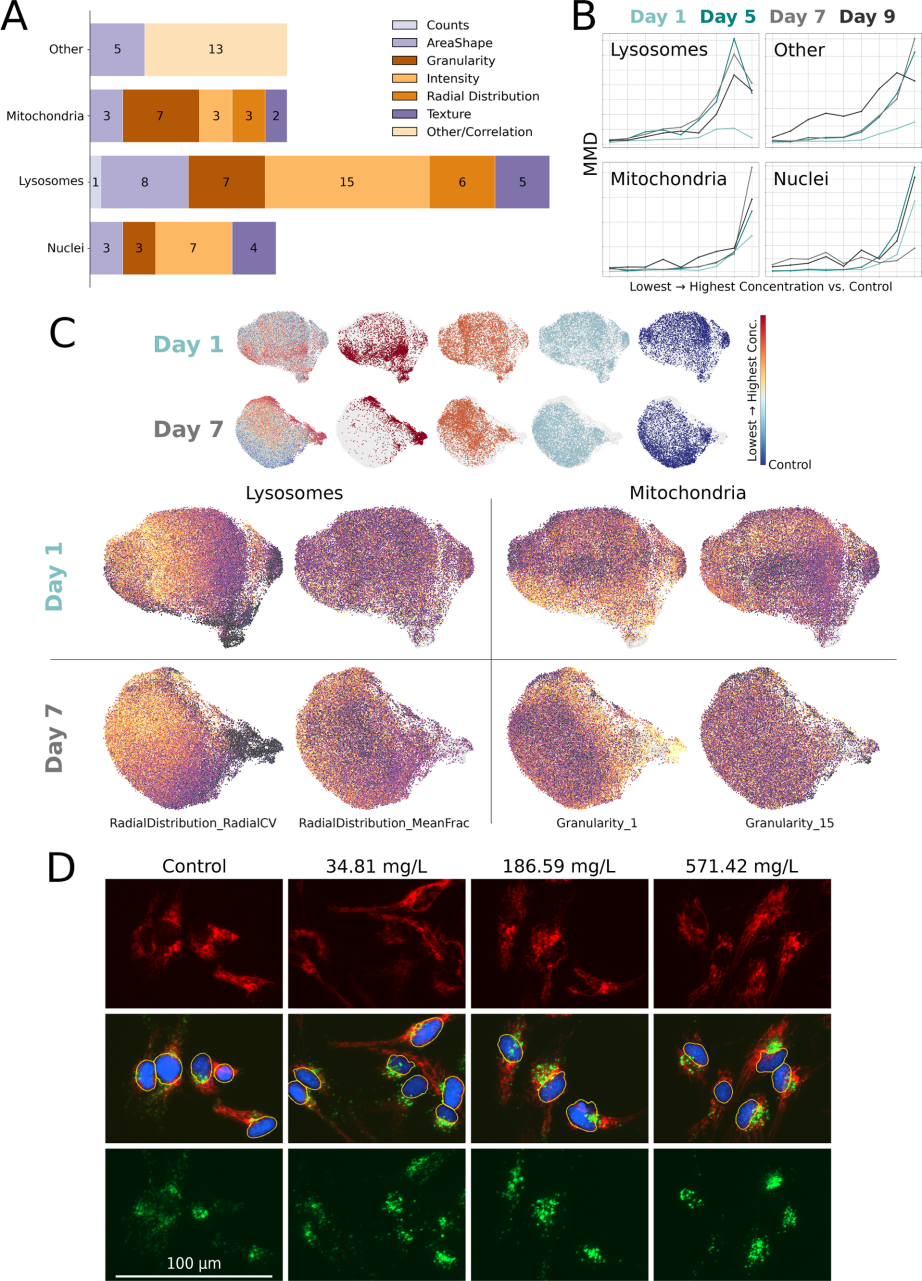
Feature-based dissection of Tebuthiuron-induced phenotypic alterations. (A) Distribution of 94 selected morphological features across CellProfiler feature categories and cellular compartments. (B) MMD scores for individual organelles showing compartment-specific sensitivity to Tebuthiuron exposure. (C) UMAP embeddings colored by representative features (RadialCV Lysosomes and RadialDistribution Mitochondria) showing temporal progression of morphological changes from day 1 to day 7. (D) Representative fluorescence microscopy images showing control cells and cells exposed to Tebuthiuron for 7 days, with nuclei (blue), lysosomes (green), and mitochondria (red).

Comparison with the Pearson et al. method^24^ revealed that our concentration–response approach captured greater morphological divergence at higher exposure concentrations, as evidenced by MMD scores (Supporting Information—Section S8). While both methods produced similar overall patterns, demonstrating the robustness of phenotypic profiling approaches, our method specifically retained features relevant to concentration-dependent phenotypes.

#### The Effect of Similar Features

To address redundancy in the selected features, we removed highly correlated features (Pearson correlation ≥0.9). This step was critical as redundant features can create artifacts in dimensionality reduction techniques like UMAP, potentially resulting in less meaningful or less accurate embeddings, producing false clustering patterns or obscuring genuine biological variation.^39^ Without correlation-based filtering, multiple readings of similar features (in our example, multiple similar high lysosomal signals—Supporting Information—Section S9) resulted in a small cluster of cells unrelated to the concentration–response effects (Figure 3, **no correlation selection**). Following correlation-based selection, UMAP embeddings more accurately reflected concentration-dependent morphological changes rather than technical redundancy. Additionally, MMD separation at the highest exposure concentrations was less prominent where there was no correlation selection (Figure 3, **no correlation selection**). The final feature set comprised 94 features that demonstrated both concentration–response behavior and nonredundancy, providing a robust foundation for mechanistic interpretation of cellular responses to chemical exposure.

### Dimensionality Reduction Enables Phenotypic Visualization

Following feature selection, we still faced the challenge of comparing high-dimensional data sets containing multiple feature types with diverse statistical distributions. High-dimensional feature profiles are often prone to the drawback of dimensionality, where traditional distance metrics become less meaningful as feature counts increase.^23^ The choice of distance metric is therefore crucial for exploiting the structure of the available features. For feature-level comparisons, we applied EMD rather than commonly used *Z*-scores to capture population-level distribution modalities without assuming normality.^24^ Pearson et al.^24^ already introduced EMD as a superior metric to compare unevenly distributed features in phenotypic profiling. Orlova et al.^49^ have proven EMD particularly suitable for drug response data due to its insensitivity to instrument drift and other technical variations.

To compare high-dimensional populations with multiple features across exposure concentrations for each day, we evaluated three distance metrics. MMD^38^ was selected as our primary metric for its flexibility in capturing complex multivariate feature interactions and robust high-dimensional performance.^50,51^ We compared MMD performance against Mahalanobis^40^ and EMD. Although Mahalanobis distance has been applied to HCS data previously, it has known limitations, such as potential covariance matrix instability in high dimensions^52^ and the need for robust estimation techniques.^53^

MMD and EMD performed similarly, with MMD showing clearer separation at higher exposure concentrations. Both metrics retained temporal and concentration-dependent responses across different processing conditions (Supporting Information—Section S10), demonstrating consistent performance across the tested processing conditions for evaluation of high-dimensional feature profiles. Mahalanobis distance proved less sensitive in separating both temporal and concentration-dependent responses (Supporting Information—Section S10), increasing the risk of failing to detect subtle but biologically relevant morphological shifts at lower exposure concentrations.

For single cell visualization purposes, we applied UMAP dimensionality reduction, which provided critical assessment of processing steps’ effectiveness, revealing high sensitivity to batch correction and feature redundancy removal (Figure 3). While UMAP highlighted false clustering patterns and batch effects when processing steps were omitted, it consistently retained some concentration–response structure across all processing combinations, confirming both the importance of systematic data preparation and the robustness of the underlying biological signal.^39^

### Phenotypic Profiles for Hypotheses Generation

Although the connection between specific image-derived features and their underlying biological mechanisms is not always straightforward, we believe these multidimensional phenotypic profiles can provide a powerful framework for hypothesis generation in the context of in vitro long-term chemical exposure. To translate our high-content data into biological hypotheses, we categorized the 94 most informative features by cellular compartment and feature type. The majority of these features originated from the lysosomal channel (42 features), followed by the mitochondrial (18) and nuclear (16) compartments (Figure 5A).

An organelle-specific MMD analysis revealed a clear and concentration-dependent increase in phenotypic divergence for all organelles (Figure 5B). The lysosomal MMD showed the strongest and most gradual increase, suggesting that this compartment was most sensitive to Tebuthiuron; however, the signal dropped at the highest concentration, likely reflecting a highly stressed phenotype. In contrast, the mitochondrial and nuclear compartments remained relatively stable across most concentrations and exposure days.

To illustrate and evaluate how each cellular compartment and feature category contributed to phenotypic separation, we examined representative features that quantify lysosomal redistribution and mitochondrial fragmentation in 2D UMAP space (Figure 5C). The RadialCV feature represents the coefficient of variation of fluorescence intensity within concentric slices extending from the cell’s periphery toward the nucleus and captures the spatial redistribution of lysosomes between the peripheral and perinuclear regions. The MeanFrac reflects the balance of lysosomal signal intensity across radial zones within cytoplasm. The two mitochondrial granularity features provide a measure of mitochondrial fragmentation (granularity 1) and fusion (granularity 15) tendencies. Lysosomal UMAP on day 1 showed limited separation across exposure levels; however, by day 7, subclusters emerged, suggesting heterogeneity and redistribution of lysosomes within the cytoplasm. Mitochondrial UMAP projections showed quantitative changes in fine or larger-scale textural variation within days and chemical exposure, pointing to significant changes in the mitochondrial network.

These quantitative changes were directly corroborated by fluorescence microscopy (Figure 5D). RTgill-W1 control cells exhibited an elongated and filamentous mitochondrial network distributed across the cytoplasm, characteristic of healthy cells. At low chemical concentration, thinning of mitochondria filaments and reduced branching could be observed. Midconcentration mitochondria appeared increasingly punctate and shortened, forming discrete dotted structures, while at the highest concentration, the mitochondria signal showed small and rounded fragments with overlap between mitochondrial and nuclear compartments. Under control conditions, lysosomes appeared as small, discrete puncta evenly distributed throughout the cytoplasm. At low chemical concentrations, some individual vesicles appeared slightly enlarged and more numerous, while at higher concentrations, lysosomes showed signs of clustering and shifting to a more perinuclear region. Finally, the highest concentration showed pronounced perinuclear accumulation, with reduced presence in the peripheral cytoplasmic region, indicative of altered vesicular trafficking and potential stress-induced remodelling of the endolysosomal compartment.

The intracellular positioning of lysosomes is known to be tightly regulated by nutrient and energy availability. Under nutrient-rich conditions, lysosomes are dispersed toward the cell periphery, where they participate in endocytosis and plasma membrane repair, whereas nutrient starvation or metabolic stress causes their relocation toward the perinuclear region.^54,55^ Similarly, mitochondrial network fragmentation and puncta formation indicated increased mitochondrial stress. Notably, the lysosomal compartment showed detectable phenotypic alterations at lower concentrations than mitochondria, as reflected in both MMD-based feature separation and single-cell UMAP distributions, suggesting that lysosomal remodeling may precede mitochondrial fragmentation in the Tebuthiuron stress cascade, despite Tebuthiuron being a soil-applied herbicide whose intended mode of action is the inhibition of photosynthetic electron transport.^28^ While mitochondrial dysfunction is widely recognized as a central integrator of chemical and oxidative stress,^56,57^ our findings indicate that lysosomal remodeling represents an early phenotypic indicator of cellular stress in RTgill-W1 cells under Tebuthiuron exposure. These early lysosomal responses may reflect altered vesicular trafficking, acidification, or autophagic signaling. The relationship between lysosomal and mitochondrial alterations under these conditions remains to be established. This observation emphasizes the importance of lysosomal readouts in high-content assays for detecting initial cellular adaptation that precedes mitochondrial damage and loss of viability in fish cells. Previous work has highlighted the importance of lysosomal early responses in fish cells under cyanobacterial toxin stress,^58^ while a more recent image-based study has emphasized mitochondrial morphology (surface area) as a sensitive indicator of chemical stress in the rainbow trout liver cell line, RTL-W1 cells.^15^ However, to our knowledge, no studies have systematically examined lysosomal and mitochondrial phenotypes in parallel under chemical exposure in fish cell systems. The present data thus provide an initial framework to explore how these two organelles interact and whether lysosomal signaling modulates downstream mitochondrial fragmentation and loss of proliferative capacity.

### Methodological Implications and Next Steps

Our systematic evaluation demonstrates that the pipeline performs reliably across a range of processing conditions, with the underlying biological signal remaining detectable even in the absence of any computational preprocessing steps (Figure 5). However, each processing step contributed to signal clarity and interpretability. Standardization and feature selection emerged as the most critical components, with standardization effectively eliminating batch effects and concentration–response-guided feature selection ensuring retention of toxicologically relevant morphological features. Our two-step row-plate correction and standard *Z*-score normalization yielded similar results, providing flexibility in method choice without compromising analytical precision.

The integration of concentration–response relationships into feature selection represents a key methodological contribution for ecotoxicological applications, where concentration-dependent effects are fundamental to risk assessment. Unlike existing RTgill-W1 HCS studies that rely primarily on pairwise treated-vs-control comparisons,^13,26^ our approach specifically retains features that exhibit concentration-dependent responses across exposure gradients, extending the analytical scope from acute, high-throughput chemical screening to chronic, multitime point exposures. Organelle-specific resolution includes lysosomes, which are absent from standard Cell Painting panels and showed the strongest early phenotypic response in this data set. This approach ensures that selected features are directly relevant to toxicological end points. The MMD metric proved particularly valuable for quantifying population-level morphological changes across multiple concentrations and time points, providing a robust framework for concentration–response characterization in high-dimensional feature space.

While our computational pipeline was tailored to the specific experimental design of this proof-of-concept study, the core analytical concepts, systematic quality control, batch effect correction, concentration–response-guided feature selection, and MMD-based population comparison, represent broadly applicable practices for phenotypic profiling data analysis in long-term, subcytotoxic exposure scenarios. Implementation in different experimental contexts will require adaptation of parameters, such as plate layout, number of exposure concentrations, biological and technical replicates, time points, and CellProfiler compartment nomenclature. Similarly, the manual optimization of segmentation parameters and the training of image-level quality control classifiers applied here would need to be repeated for each new experimental context, which has implications for interstudy comparability. Future adoption of more advanced or automated segmentation methods^59,60^ could reduce this burden and improve reproducibility and quality control, while leaving the analytical concepts intact.

The Tebuthiuron-induced phenotypic profile established here, characterized by lysosomal spatial redistribution and mitochondrial fragmentation as the dominant early responses, provides an initial reference point against which future profiles can be compared. In a multichemical context, the strategy for feature selection will require further consideration: whether features should be selected independently for each compound or defined as a shared set applied across a chemical library carries different implications for profile comparability and pattern-based classification of stress responses. While this simplified marker set enabled robust extraction of organelle-level phenotypes within a scalable workflow, it limits mechanistic interpretation because additional cellular compartments and signaling pathways that may contribute to toxicological responses are not captured. Consequently, the present profiles should be interpreted primarily as phenotypic descriptors that support hypothesis generation rather than comprehensive mechanistic resolution. Broader validation across chemical classes and experimental systems (for example additional staining) will ultimately be required before this framework can contribute to environmental hazard assessment in a regulatory context, but the analytical concepts demonstrated here are designed to remain applicable as the experimental scope expands.

## Supplementary Material



## Data Availability

All data is available in the Zenodo repository at 10.5281/zenodo.17951792 (release v1 ^41^). All Python scripts and additional files used in this manuscript are available in the GitHub repository at https://github.com/NIB-SI/HCS-proc.git (release v1 ^42^). Any additional supporting code is available upon request.

## Notes

Additional UMAP embeddings and feature maps (S1–S2), image quality-control classifier performance and segmentation artifacts (S3–S4), standardization (S5–S6) and feature-selection comparisons (S8–S9), concentration–response experiment data including nominal-vs-measured concentrations (S7), distance-metric comparisons (S10), the full feature list (S11), and correlation-based feature removal (S12) (PDF)

## Abbreviations

AB: alamarBlue
AIC: Akaike information criterion
ARIS: Slovenian Research and Innovation Agency
BIC: Bayesian information criterion
BR: biological replicate
CFDA-AM: 5-carboxyfluorescein diacetate, acetoxymethyl ester
CI: confidence interval
DMSO: dimethyl sulfoxide
EC_50_: half maximal effective concentration
EMD: earth mover’s distance
FBS: fetal bovine serum
HCS: high-content screening
IQR: interquartile range
MMD: maximum mean discrepancy
QC: quality control
RMSE: root mean square error
SNSF: Swiss National Science Foundation
TMRM: tetramethylrhodamine, methyl ester
TR: technical replicate
UMAP: Uniform Manifold Approximation and Projection for dimension reduction
