# Supplementary material for: A High-Content Imaging Pipeline to Investigate Subcytotoxic Effects in RTgill-W1 Cells
Source: Environ Sci Technol. 2026 Jul 29;60(31):21402–16. doi: 10.1021/acs.est.5c18316 (PMC13472083; doi:10.1021/acs.est.5c18316)
Supplement: Supplementary file 1 [file es5c18316_si_001.pdf]

Supplementary Files: A high-content imaging pipeline to investigate subcytotoxic effects in RTgill-W1 cells

Miha Tome[1], Barbara Jozef[2], Sven Lukas Mosimann[2,3], Marissa Kosnik[2], Kristin Schirmer[2,3], and Anže Županič[1]

1 - Department of Biotechnology and Systems Biology, National Institute of Biology, Ljubljana, Slovenia
2 - Environmental Toxicology, Eawag, Dübendorf, Switzerland
3 - Institute of Biogeochemistry and Pollutant Dynamics, ETHZ, Zürich, Switzerland

Table of content

Table of content 1
Concentration range 2
S1: UMAP embeddings for each day, colored by each exposure concentration 2
S2: UMAP embedding Day 5 colored by feature 7
S3: Image Quality Classifier Performance Summary 30
S4: Poor segmentations artefacts 31
S5: Z-score standardization comparison 33
S6: Concentration-response feature selection results 36
S7: Concentration-response experiments 37
S8: High variability, low activity feature selection 39
S9: Effect of multiple highly similar features on global UMAP structure 42
S10: MMD, EMD and Mahalanobis distance comparison 43
S11: List of features 44
S12: Removal of similar features 55
Full dataset 56
References 56

Concentration range

Table 0: Exposure concentration range and coloring/marking in the supplementary text

Concentration [mg/L]	Color	Marked in supplementary files
1000	#a50026	2
571.42	#d73027	3
326.530	#f46d43	4
186.588	#fdae61	5
106.62	#fee090	6
60.92	#e0f3f8	7
34.81	#abd9e9	8
19.89	#74add1	9
11.36	#4575b4	10
0	#313695	11

S1: UMAP embeddings for each day, colored by each exposure concentration

Concentrations marked: **2** is the highest exposure concentration, where **10** is the lowest exposure, **11** being untreated control.

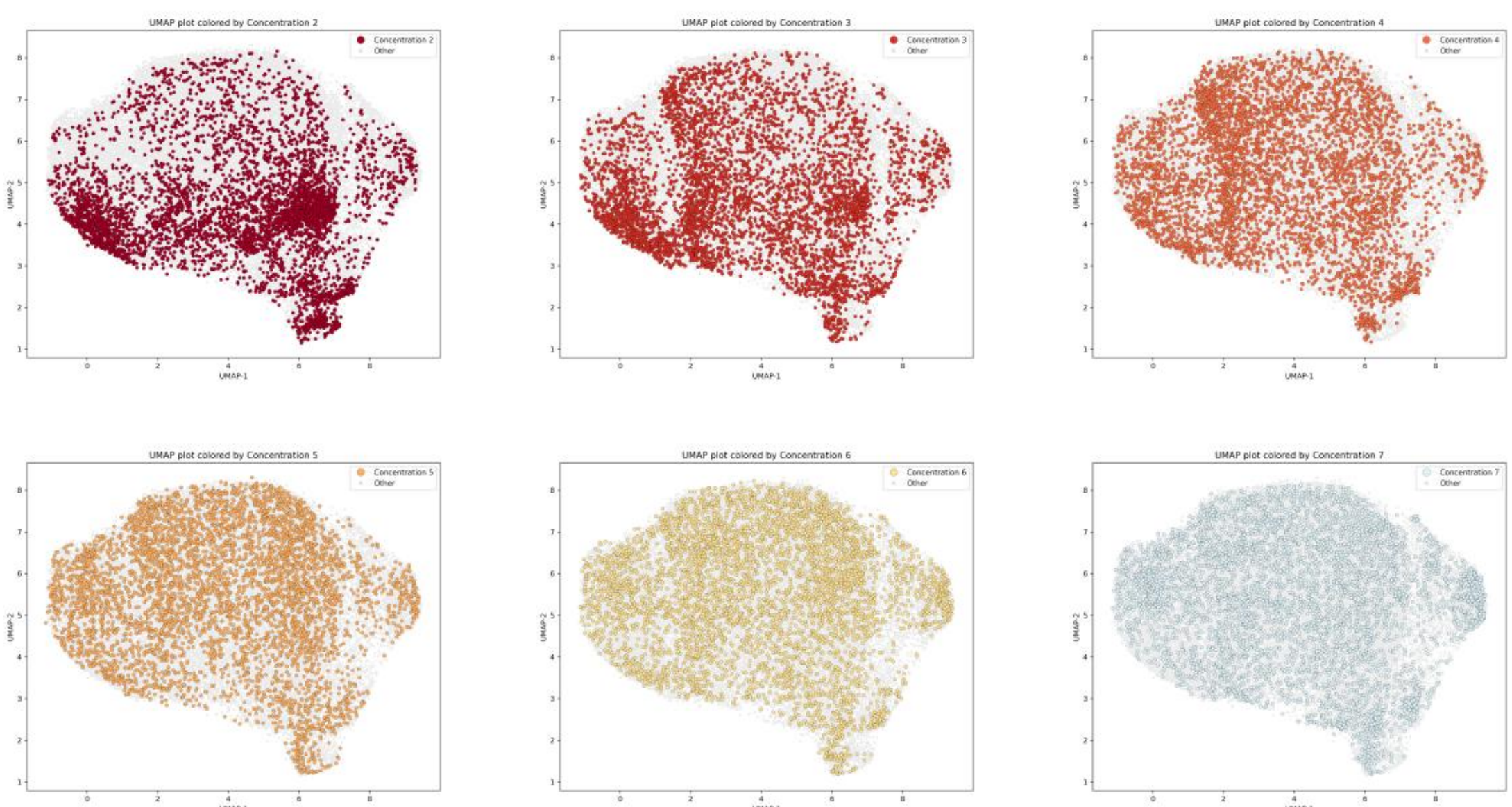

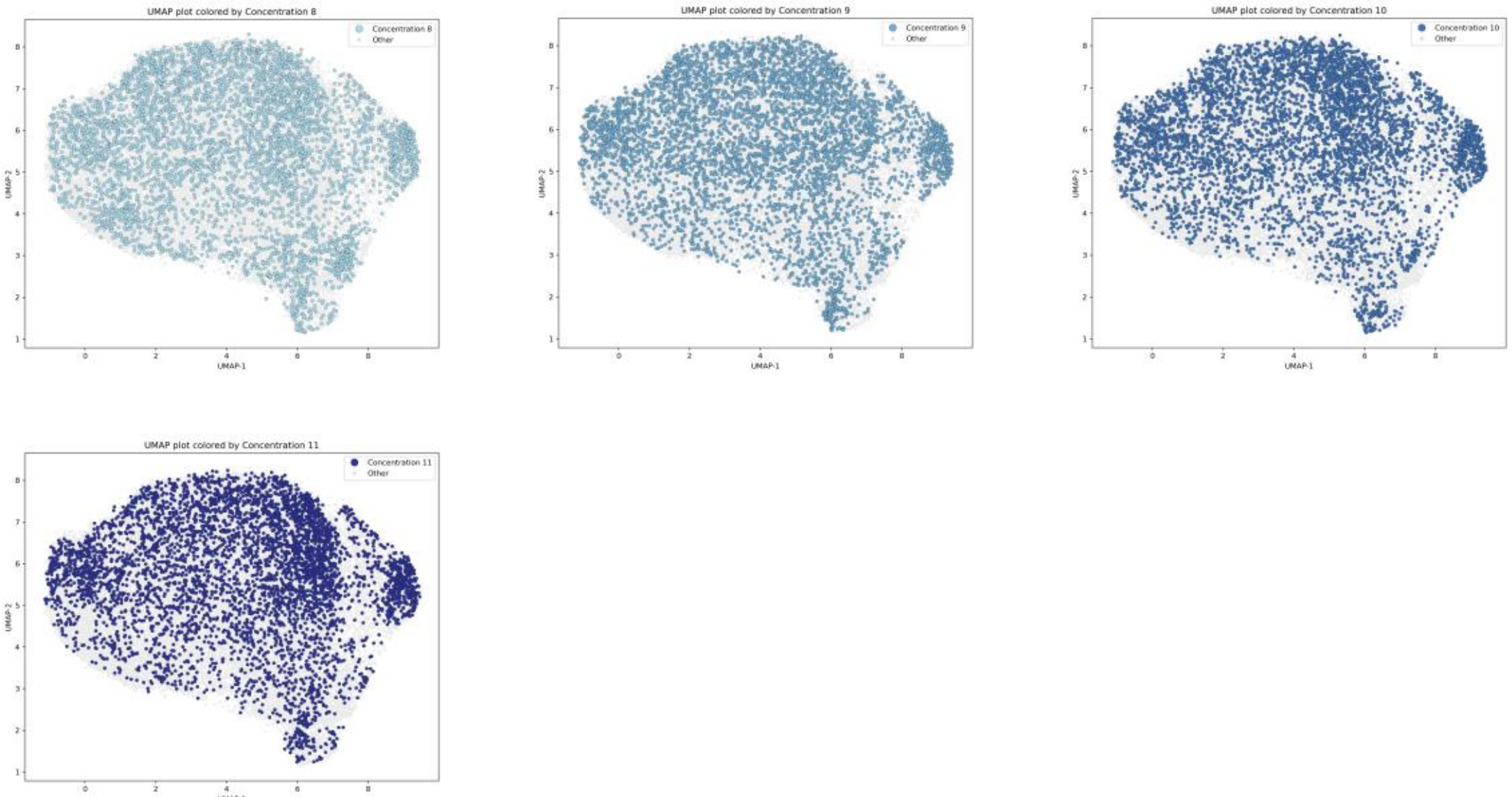

Figures S1.1: UMAP embeddings for each **Day 1**, cells colored by each concentration

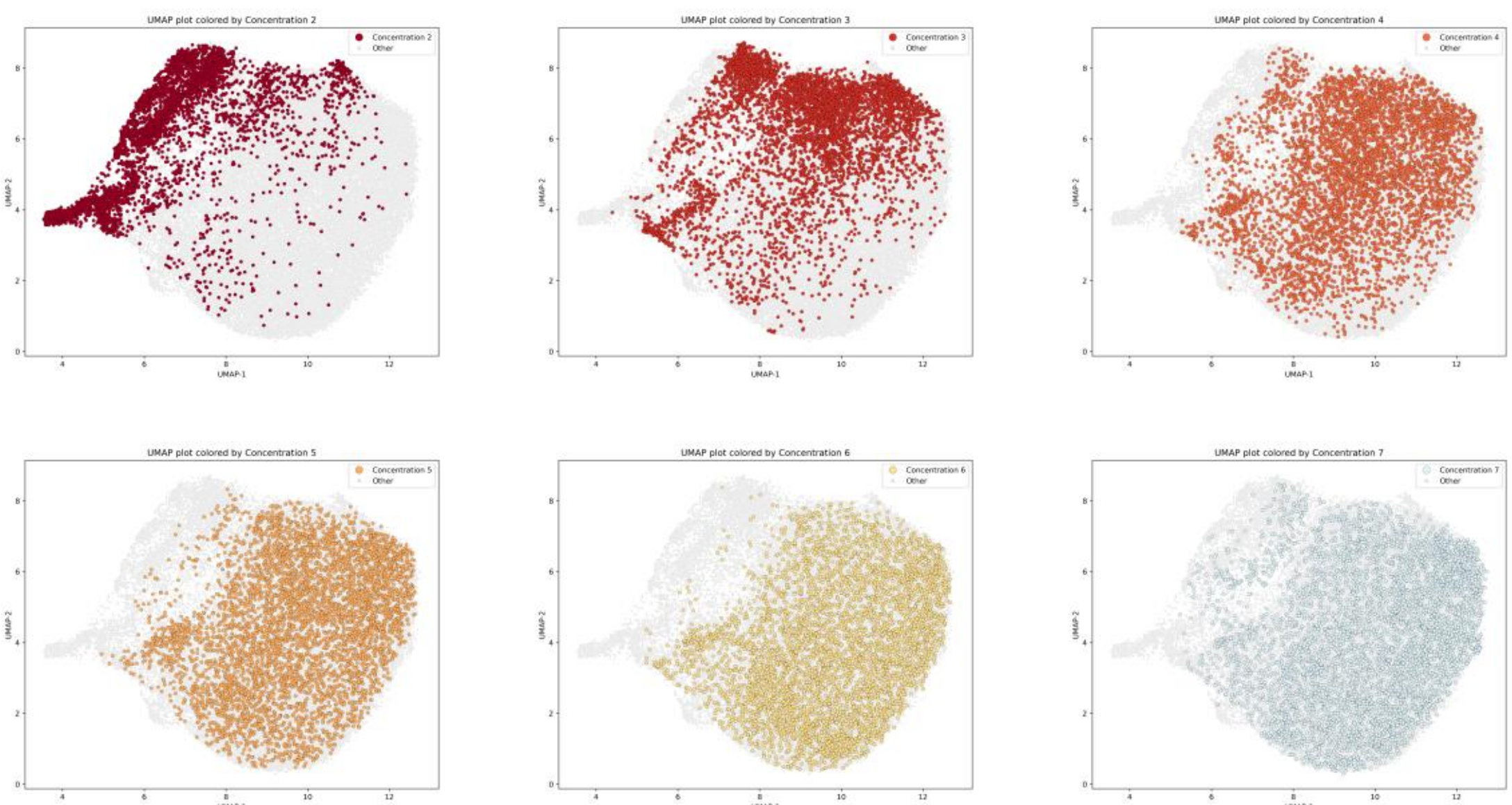

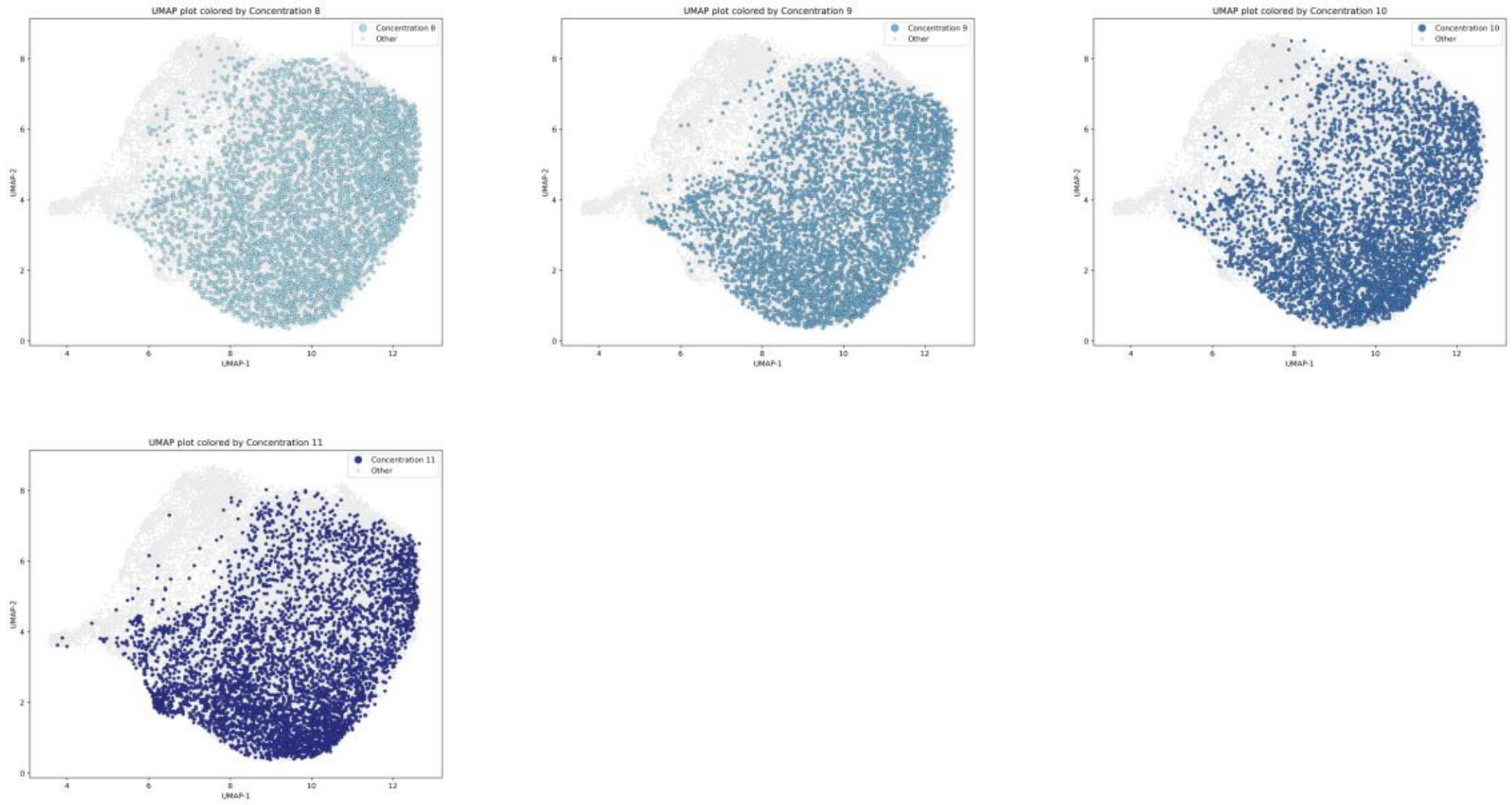

Figures S1.2: UMAP embeddings for each **Day 5**, cells colored by each concentration

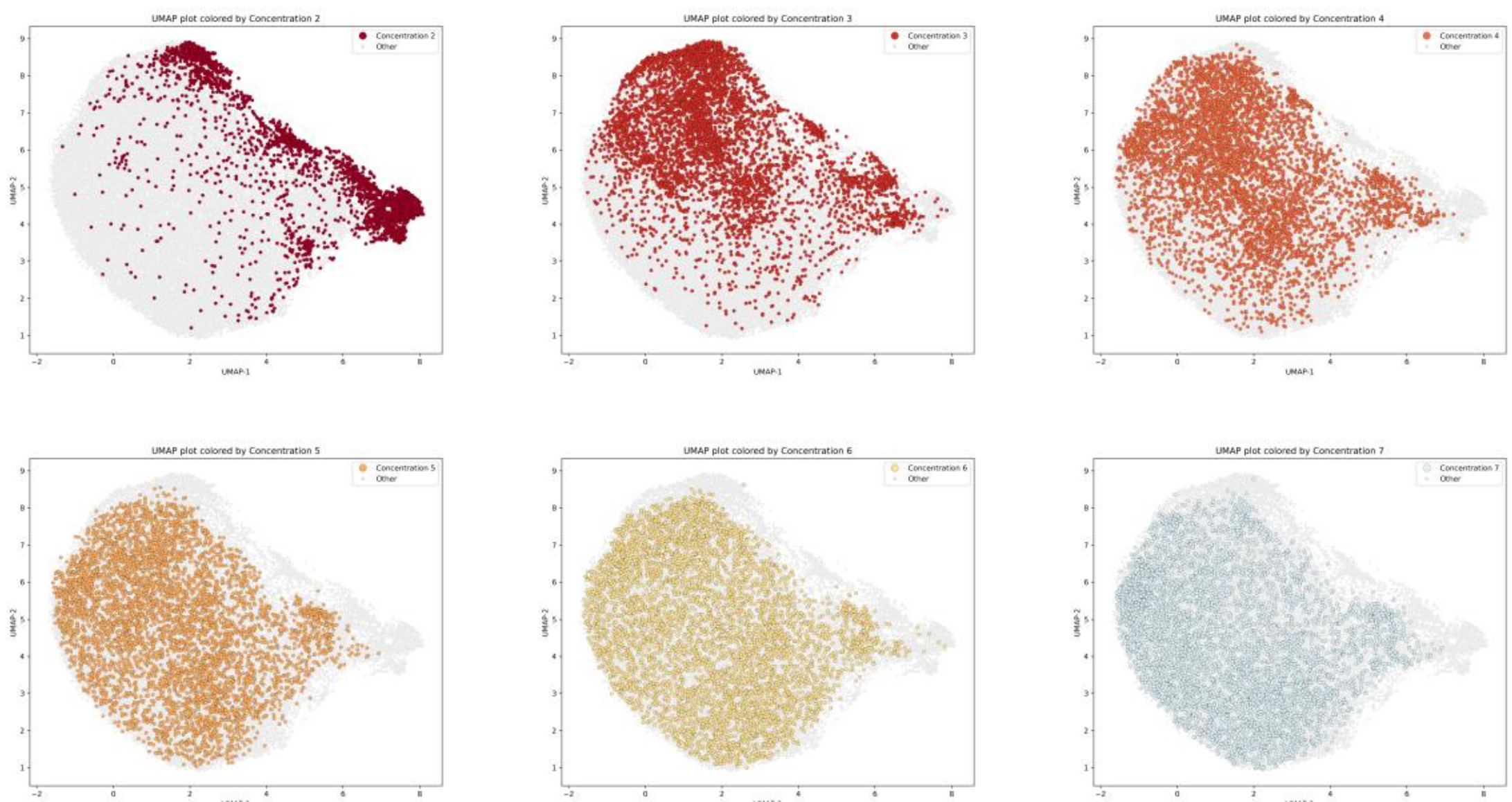

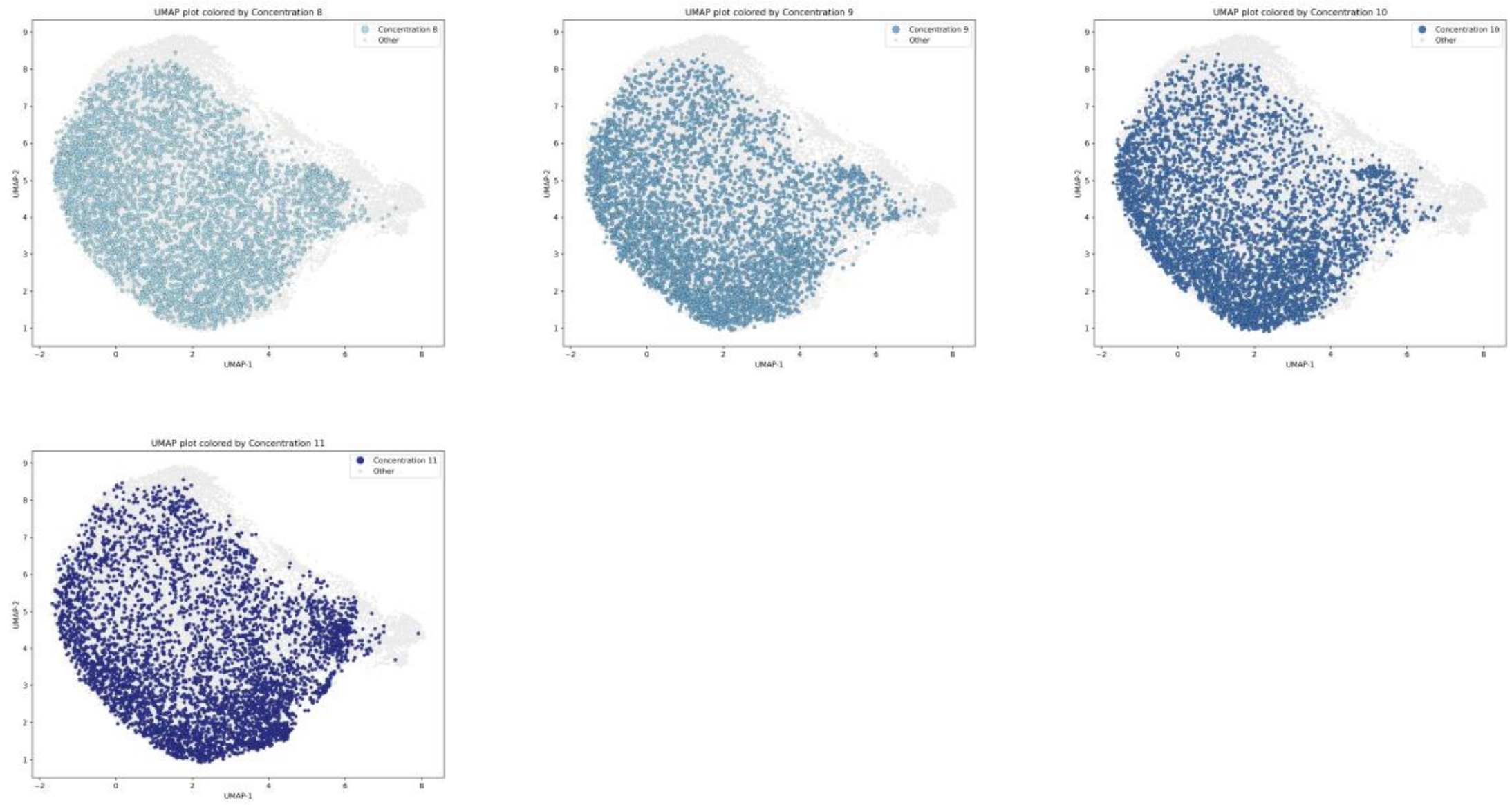

Figures S1.3: UMAP embeddings for each **Day 7**, cells colored by each concentration

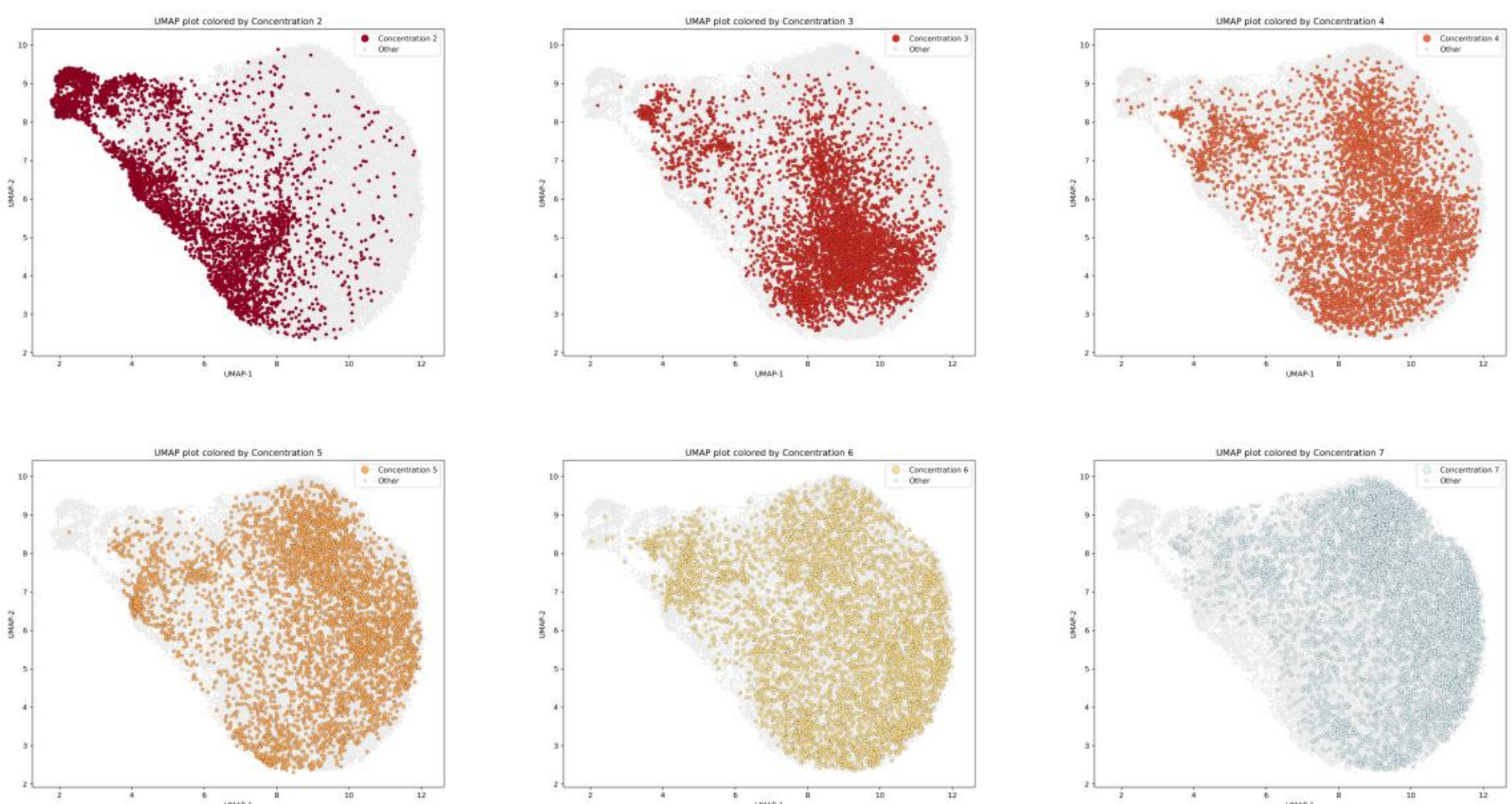

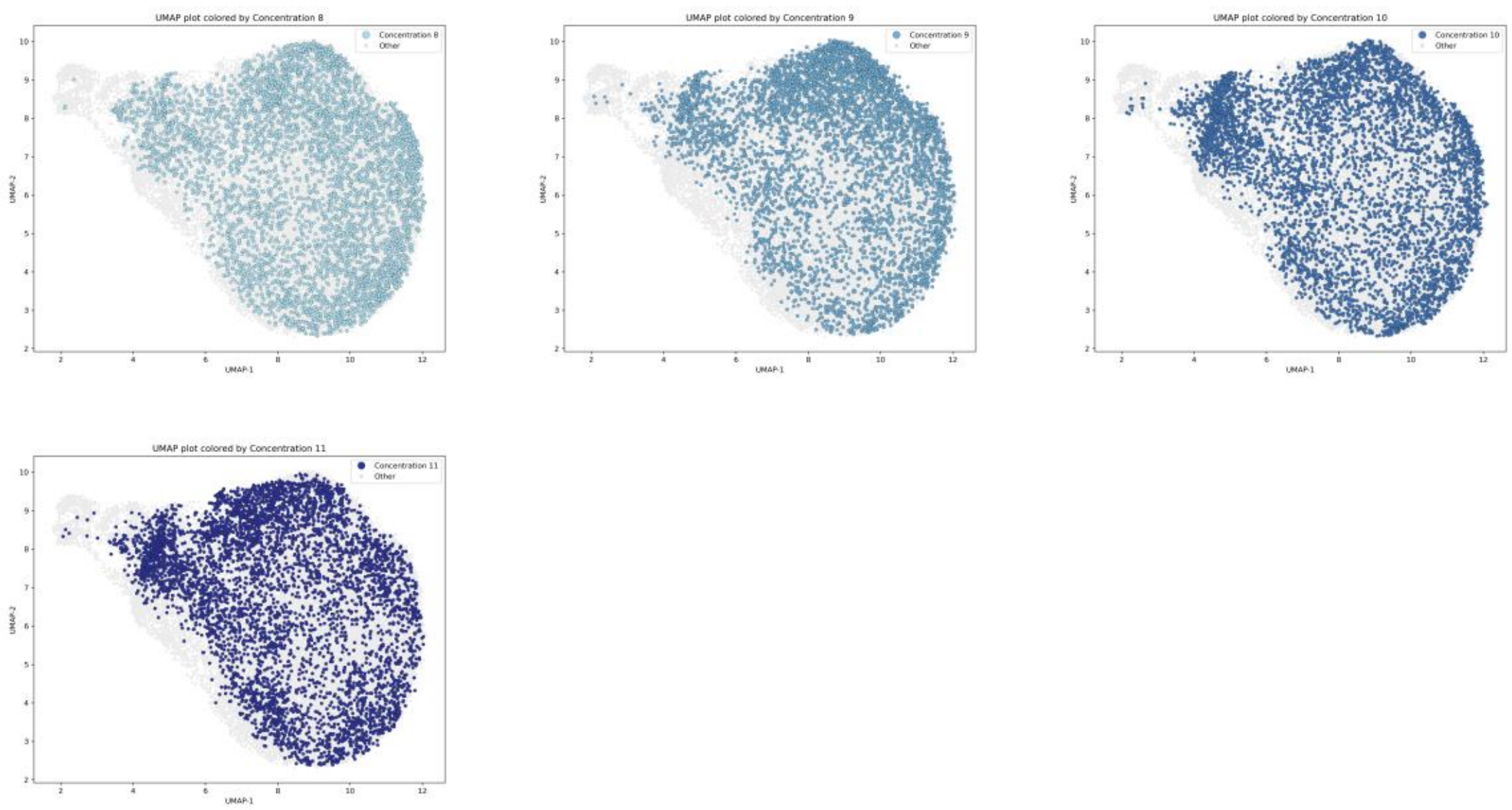

Figures S1.4: UMAP embeddings for each **Day 9**, cells colored by each concentration

S2: UMAP embedding Day 5 colored by feature

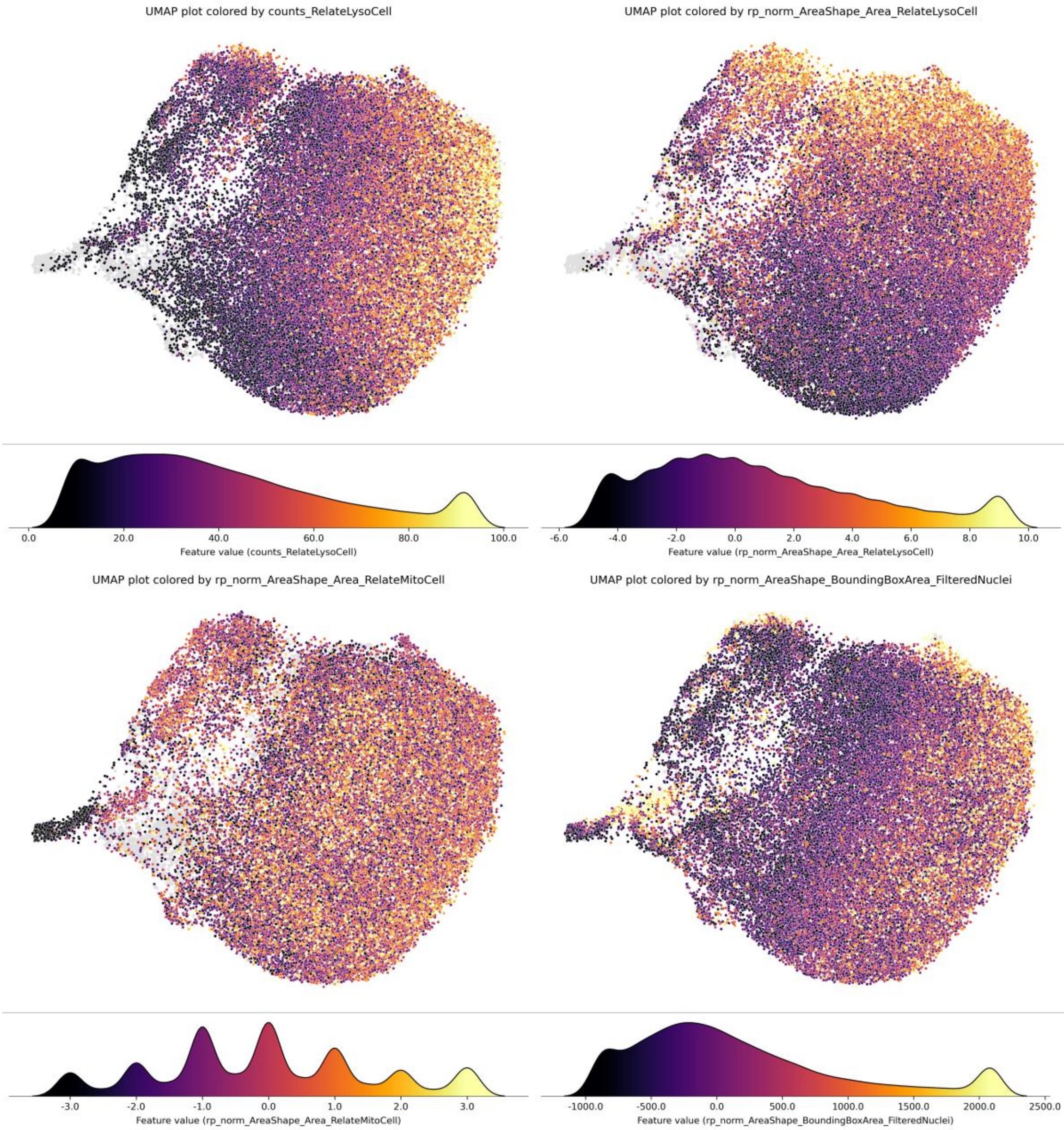

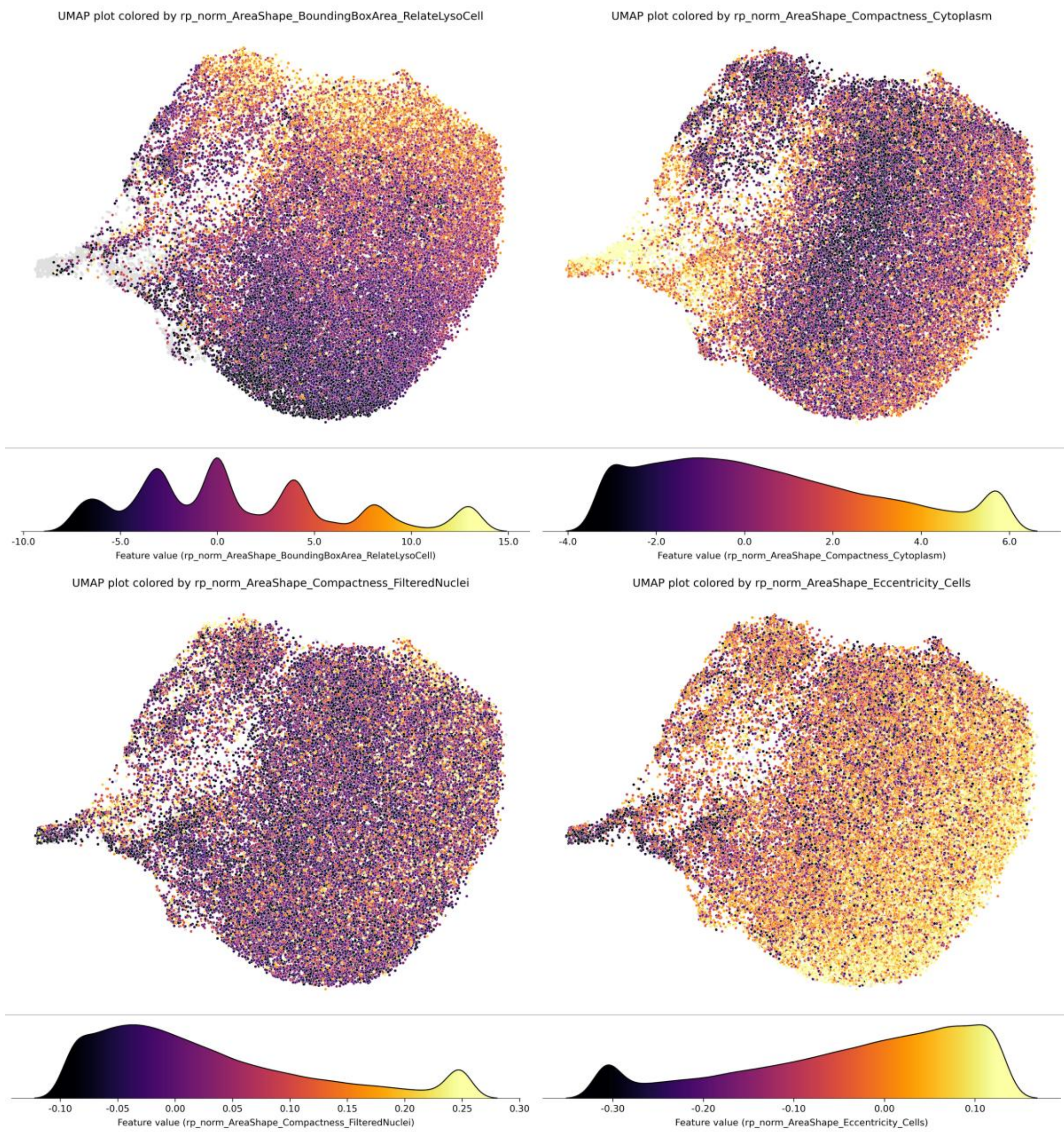
UMAP plot colored by rp_norm_AreaShape_BoundingBoxArea_RelateLysoCell
-10.0
-5.0
0.0
5.0
10.0
15.0
Feature value (rp_norm_AreaShape_BoundingBoxArea_RelateLysoCell)
UMAP plot colored by rp_norm_AreaShape_Compactness_Cytoplasm
-4.0
-2.0
0.0
2.0
4.0
6.0
Feature value (rp_norm_AreaShape_Compactness_Cytoplasm)
UMAP plot colored by rp_norm_AreaShape_Compactness_FilteredNuclei
-0.10
-0.05
0.00
0.05
0.10
0.15
0.20
0.25
0.30
Feature value (rp_norm_AreaShape_Compactness_FilteredNuclei)
UMAP plot colored by rp_norm_AreaShape_Eccentricity_Cells
-0.30
-0.20
-0.10
0.00
0.10
Feature value (rp_norm_AreaShape_Eccentricity_Cells)

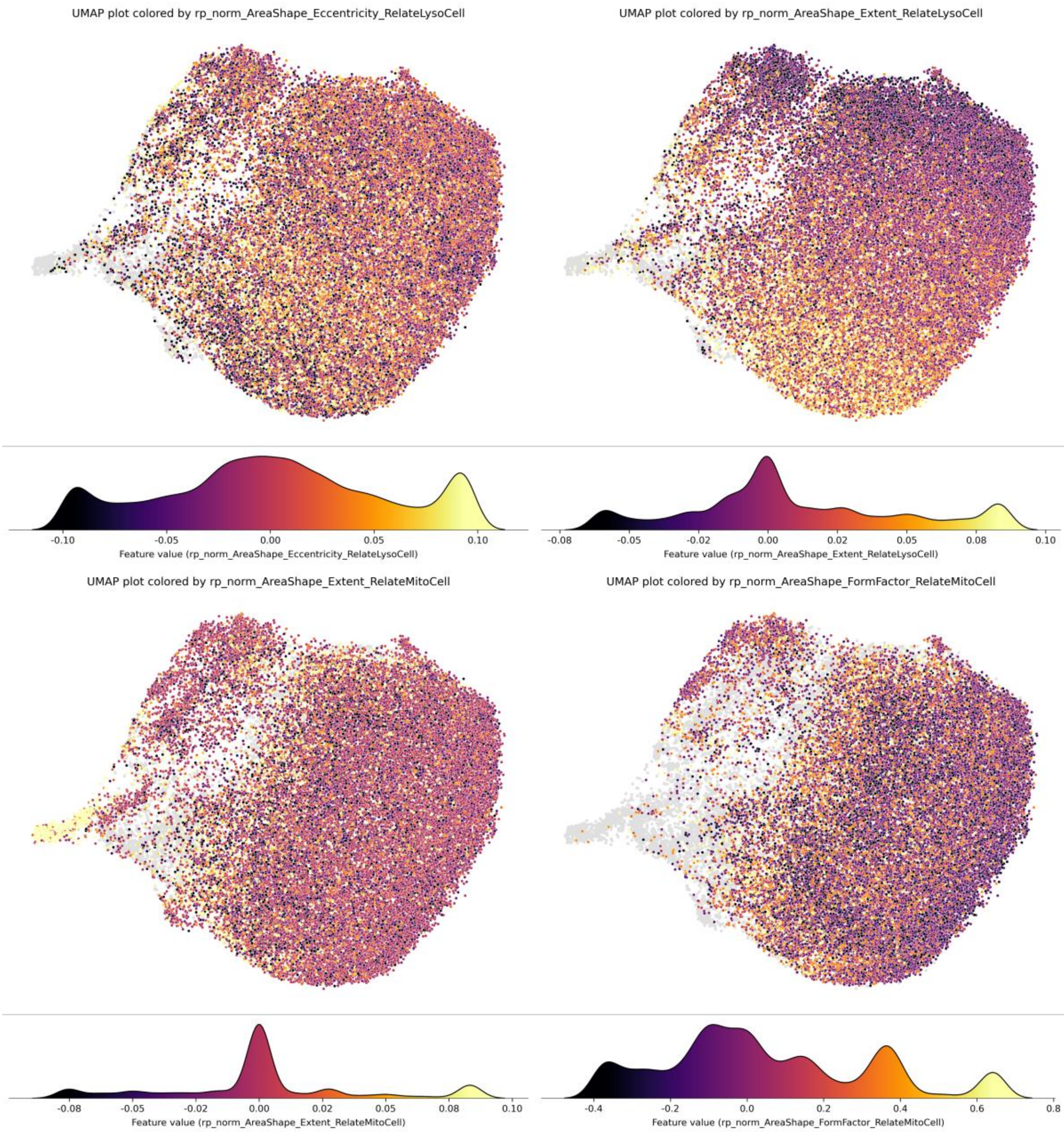
UMAP plot colored by rp_norm_AreaShape_Eccentricity_RelateLysoCell
-0.10
-0.05
0.00
0.05
0.10
Feature value (rp_norm_AreaShape_Eccentricity_RelateLysoCell)
UMAP plot colored by rp_norm_AreaShape_Extent_RelateLysoCell
-0.08
-0.05
-0.02
0.00
0.02
0.05
0.08
0.10
Feature value (rp_norm_AreaShape_Extent_RelateLysoCell)
UMAP plot colored by rp_norm_AreaShape_Extent_RelateMitoCell
-0.08
-0.05
-0.02
0.00
0.02
0.05
0.08
0.10
Feature value (rp_norm_AreaShape_Extent_RelateMitoCell)
UMAP plot colored by rp_norm_AreaShape_FormFactor_RelateMitoCell
-0.4
-0.2
0.0
0.2
0.4
0.6
0.8
Feature value (rp_norm_AreaShape_FormFactor_RelateMitoCell)

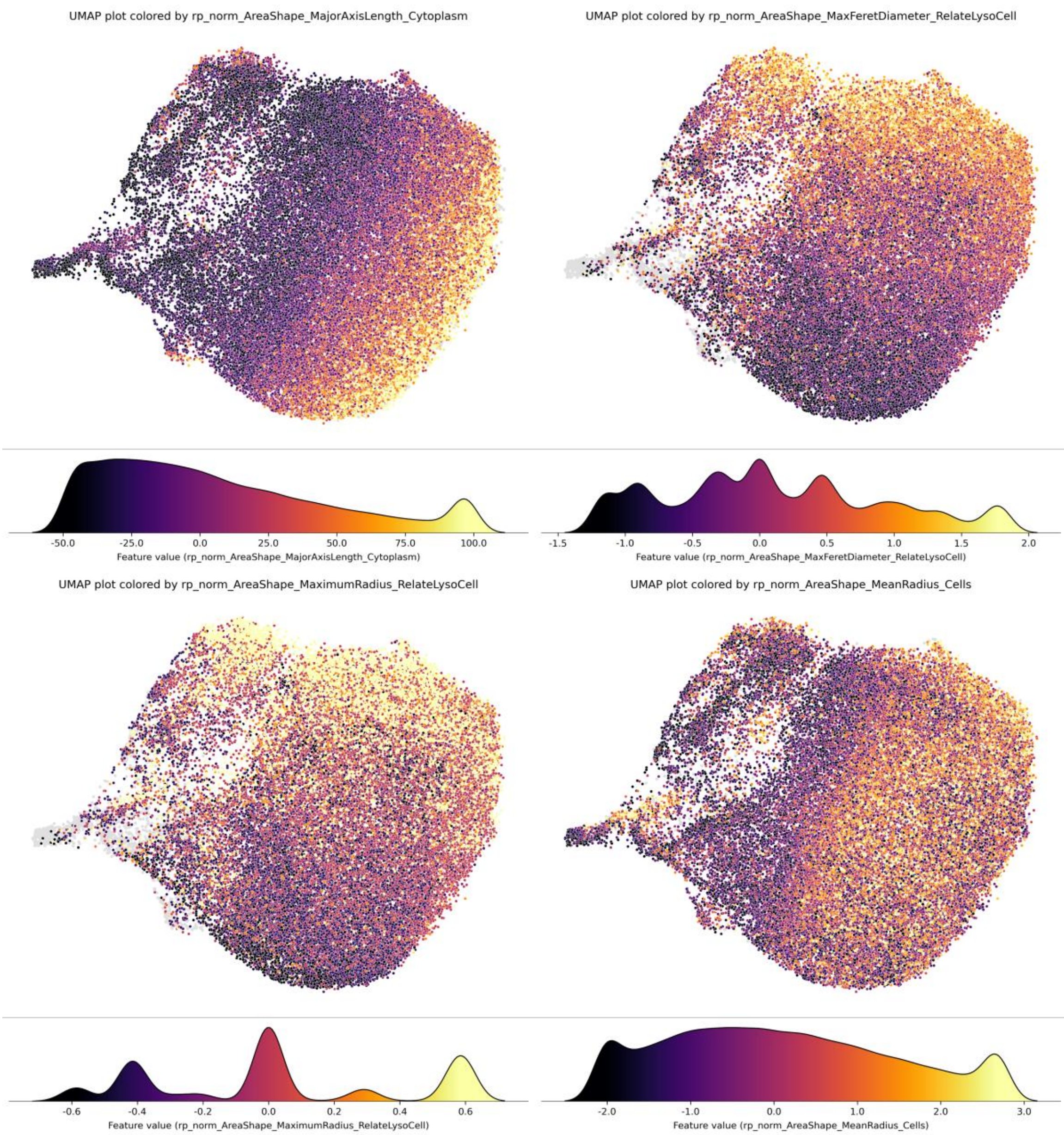
UMAP plot colored by rp_norm_AreaShape_MajorAxisLength_Cytoplasm
-50.0 -25.0 0.0 25.0 50.0 75.0 100.0
Feature value (rp_norm_AreaShape_MajorAxisLength_Cytoplasm)
UMAP plot colored by rp_norm_AreaShape_MaxFeretDiameter_RelateLysoCell
-1.5 -1.0 -0.5 0.0 0.5 1.0 1.5 2.0
Feature value (rp_norm_AreaShape_MaxFeretDiameter_RelateLysoCell)
UMAP plot colored by rp_norm_AreaShape_MaximumRadius_RelateLysoCell
-0.6 -0.4 -0.2 0.0 0.2 0.4 0.6
Feature value (rp_norm_AreaShape_MaximumRadius_RelateLysoCell)
UMAP plot colored by rp_norm_AreaShape_MeanRadius_Cells
-2.0 -1.0 0.0 1.0 2.0 3.0
Feature value (rp_norm_AreaShape_MeanRadius_Cells)

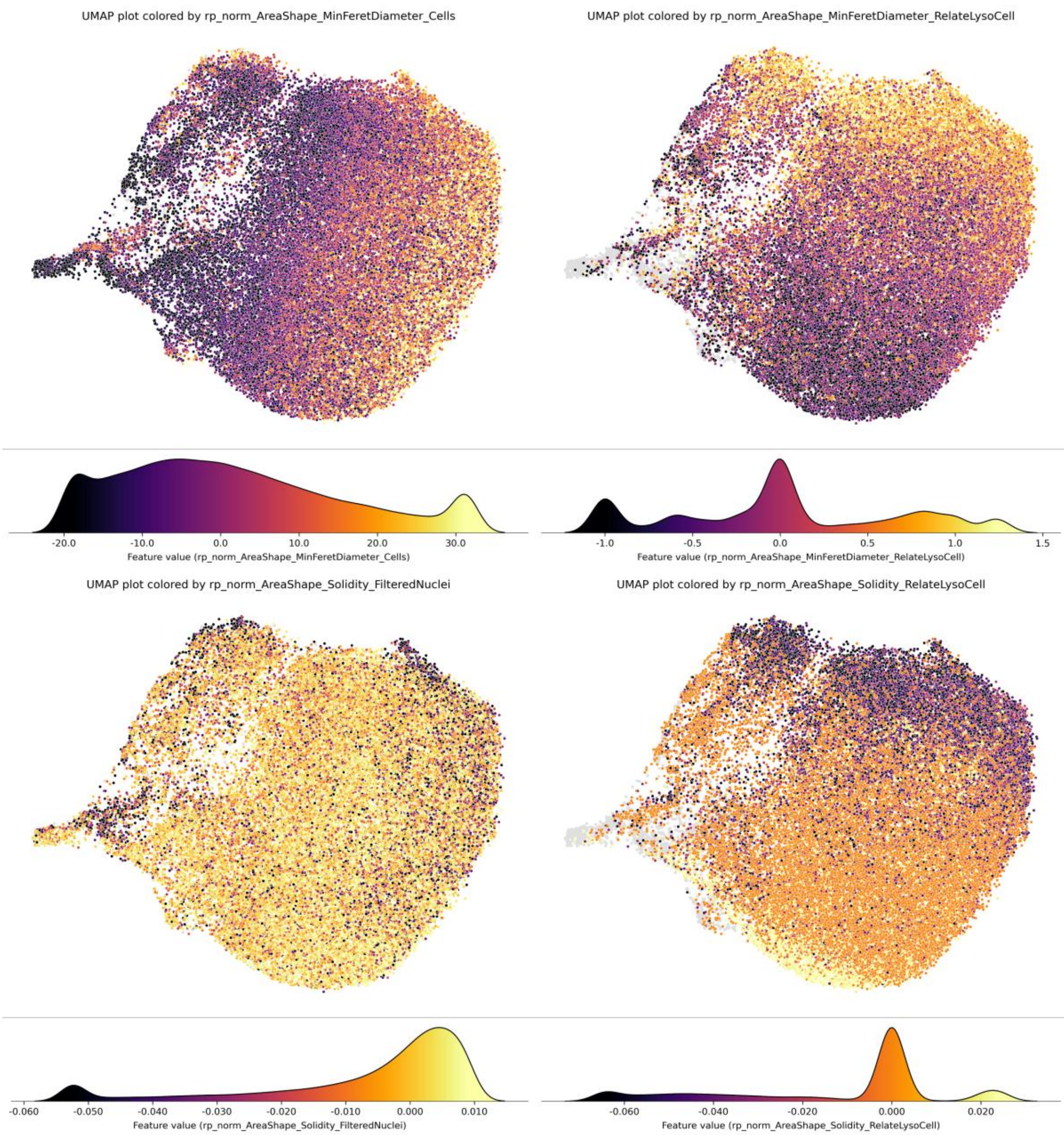
UMAP plot colored by rp_norm_AreaShape_MinFeretDiameter_Cells
-20.0
-10.0
0.0
10.0
20.0
30.0
Feature value (rp_norm_AreaShape_MinFeretDiameter_Cells)
UMAP plot colored by rp_norm_AreaShape_MinFeretDiameter_RelateLysoCell
-1.0
-0.5
0.0
0.5
1.0
1.5
Feature value (rp_norm_AreaShape_MinFeretDiameter_RelateLysoCell)
UMAP plot colored by rp_norm_AreaShape_Solidity_FilteredNuclei
-0.060
-0.050
-0.040
-0.030
-0.020
-0.010
0.000
0.010
Feature value (rp_norm_AreaShape_Solidity_FilteredNuclei)
UMAP plot colored by rp_norm_AreaShape_Solidity_RelateLysoCell
-0.060
-0.040
-0.020
0.000
0.020
Feature value (rp_norm_AreaShape_Solidity_RelateLysoCell)

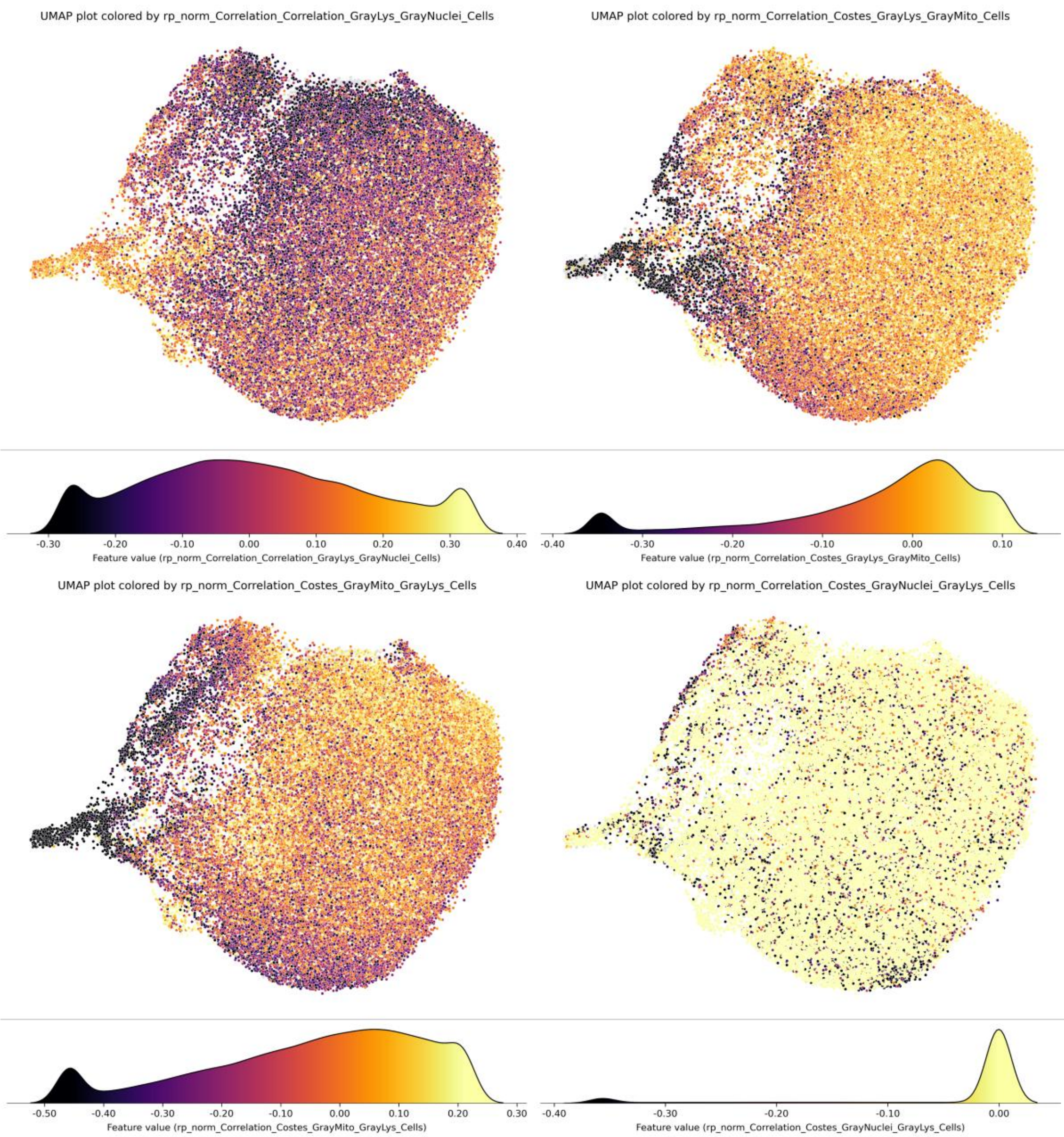
UMAP plot colored by rp_norm_Correlation_Correlation_GrayLys_GrayNuclei_Cells
UMAP plot colored by rp_norm_Correlation_Costes_GrayLys_GrayMito_Cells
-0.30 -0.20 -0.10 0.00 0.10 0.20 0.30 0.40
Feature value (rp_norm_Correlation_Correlation_GrayLys_GrayNuclei_Cells)
-0.40 -0.30 -0.20 -0.10 0.00 0.10
Feature value (rp_norm_Correlation_Costes_GrayLys_GrayMito_Cells)
UMAP plot colored by rp_norm_Correlation_Costes_GrayMito_GrayLys_Cells
UMAP plot colored by rp_norm_Correlation_Costes_GrayNuclei_GrayLys_Cells
-0.50 -0.40 -0.30 -0.20 -0.10 0.00 0.10 0.20 0.30
Feature value (rp_norm_Correlation_Costes_GrayMito_GrayLys_Cells)
-0.40 -0.30 -0.20 -0.10 0.00
Feature value (rp_norm_Correlation_Costes_GrayNuclei_GrayLys_Cells)

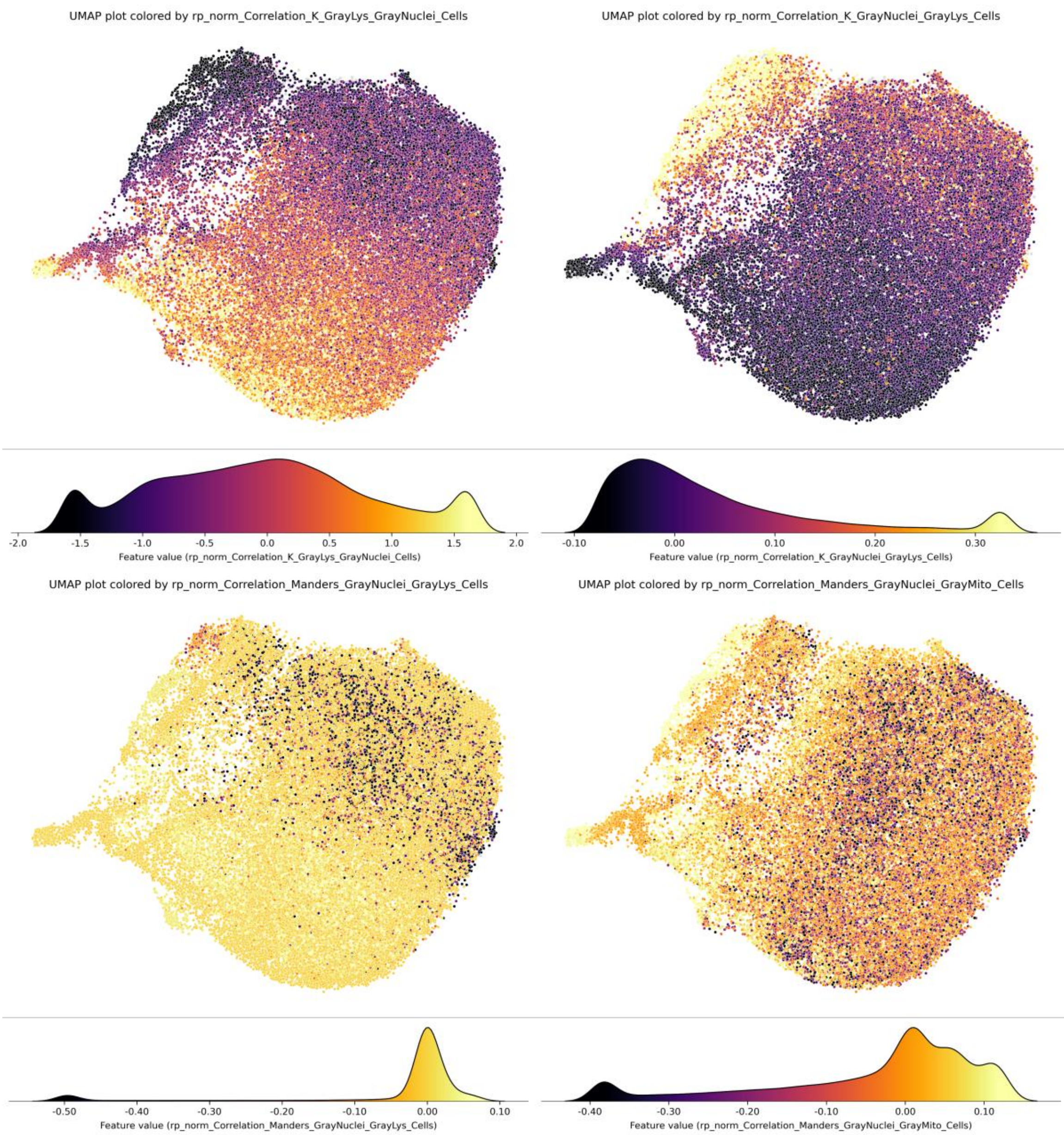
UMAP plot colored by rp_norm_Correlation_K_GrayLys_GrayNuclei_Cells
UMAP plot colored by rp_norm_Correlation_K_GrayNuclei_GrayLys_Cells
-2.0 -1.5 -1.0 -0.5 0.0 0.5 1.0 1.5 2.0
Feature value (rp_norm_Correlation_K_GrayLys_GrayNuclei_Cells)
-0.10 0.00 0.10 0.20 0.30
Feature value (rp_norm_Correlation_K_GrayNuclei_GrayLys_Cells)
UMAP plot colored by rp_norm_Correlation_Manders_GrayNuclei_GrayLys_Cells
UMAP plot colored by rp_norm_Correlation_Manders_GrayNuclei_GrayMito_Cells
-0.50 -0.40 -0.30 -0.20 -0.10 0.00 0.10
Feature value (rp_norm_Correlation_Manders_GrayNuclei_GrayLys_Cells)
-0.40 -0.30 -0.20 -0.10 0.00 0.10
Feature value (rp_norm_Correlation_Manders_GrayNuclei_GrayMito_Cells)

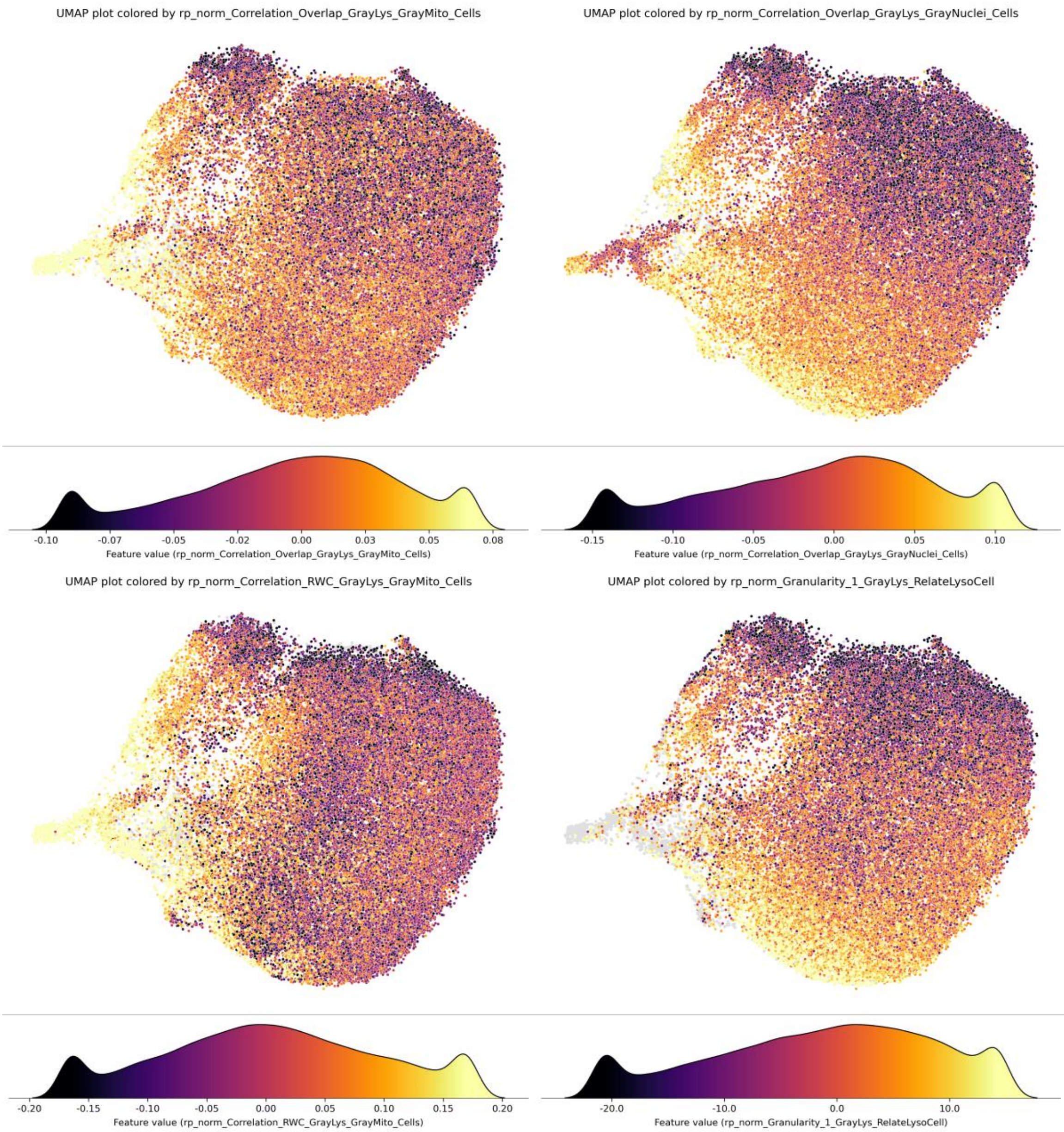
UMAP plot colored by rp_norm_Correlation_Overlap_GrayLys_GrayMito_Cells
-0.10 -0.07 -0.05 -0.02 0.00 0.03 0.05 0.08
Feature value (rp_norm_Correlation_Overlap_GrayLys_GrayMito_Cells)
UMAP plot colored by rp_norm_Correlation_Overlap_GrayLys_GrayNuclei_Cells
-0.15 -0.10 -0.05 0.00 0.05 0.10
Feature value (rp_norm_Correlation_Overlap_GrayLys_GrayNuclei_Cells)
UMAP plot colored by rp_norm_Correlation_RWC_GrayLys_GrayMito_Cells
-0.20 -0.15 -0.10 -0.05 0.00 0.05 0.10 0.15 0.20
Feature value (rp_norm_Correlation_RWC_GrayLys_GrayMito_Cells)
UMAP plot colored by rp_norm_Granularity_1_GrayLys_RelateLysoCell
-20.0 -10.0 0.0 10.0
Feature value (rp_norm_Granularity_1_GrayLys_RelateLysoCell)

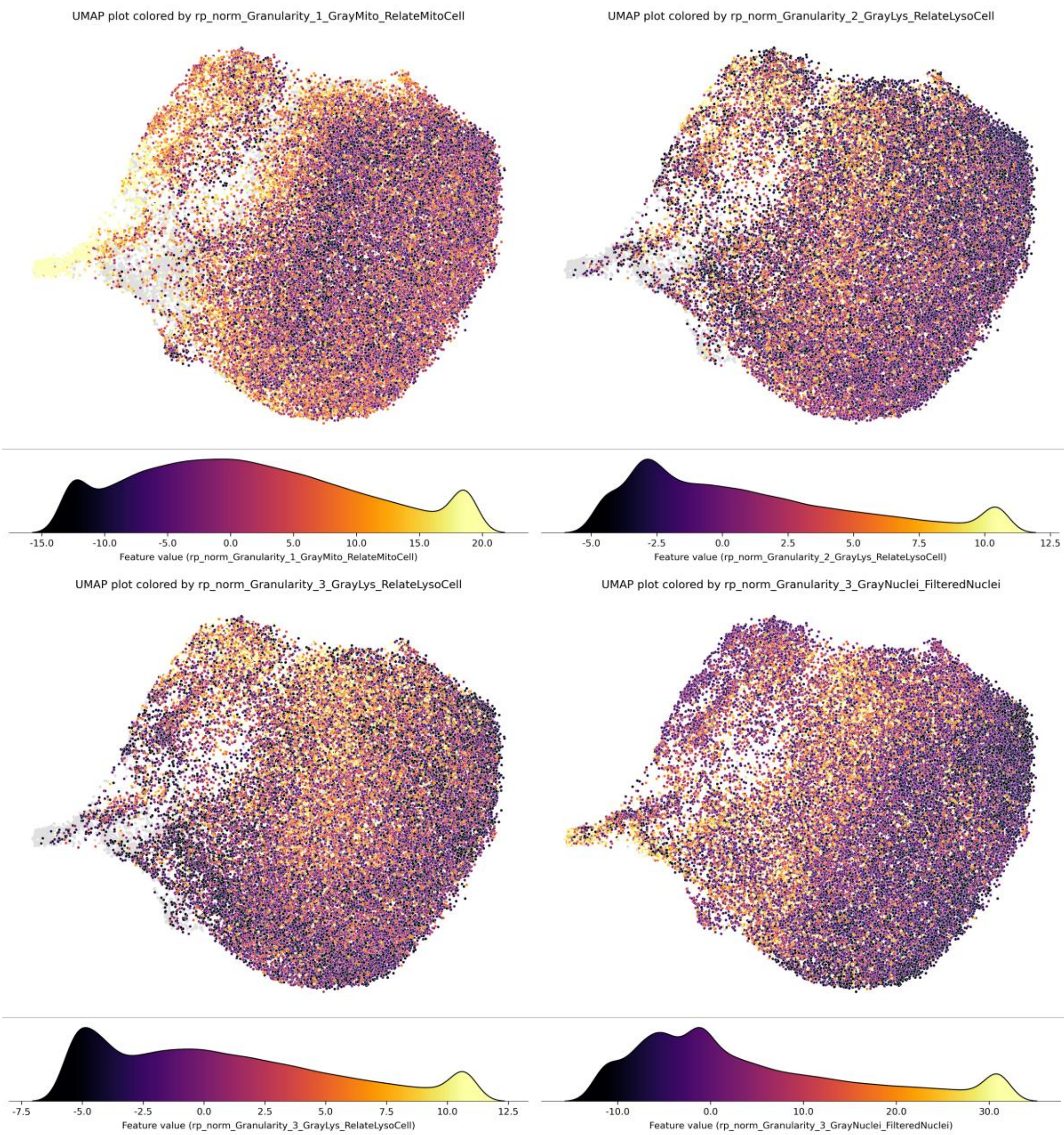
UMAP plot colored by rp_norm_Granularity_1_GrayMito_RelateMitoCell
UMAP plot colored by rp_norm_Granularity_2_GrayLys_RelateLysoCell
-15.0 -10.0 -5.0 0.0 5.0 10.0 15.0 20.0
Feature value (rp_norm_Granularity_1_GrayMito_RelateMitoCell)
-5.0 -2.5 0.0 2.5 5.0 7.5 10.0 12.5
Feature value (rp_norm_Granularity_2_GrayLys_RelateLysoCell)
UMAP plot colored by rp_norm_Granularity_3_GrayLys_RelateLysoCell
UMAP plot colored by rp_norm_Granularity_3_GrayNuclei_FilteredNuclei
-7.5 -5.0 -2.5 0.0 2.5 5.0 7.5 10.0 12.5
Feature value (rp_norm_Granularity_3_GrayLys_RelateLysoCell)
-10.0 0.0 10.0 20.0 30.0
Feature value (rp_norm_Granularity_3_GrayNuclei_FilteredNuclei)

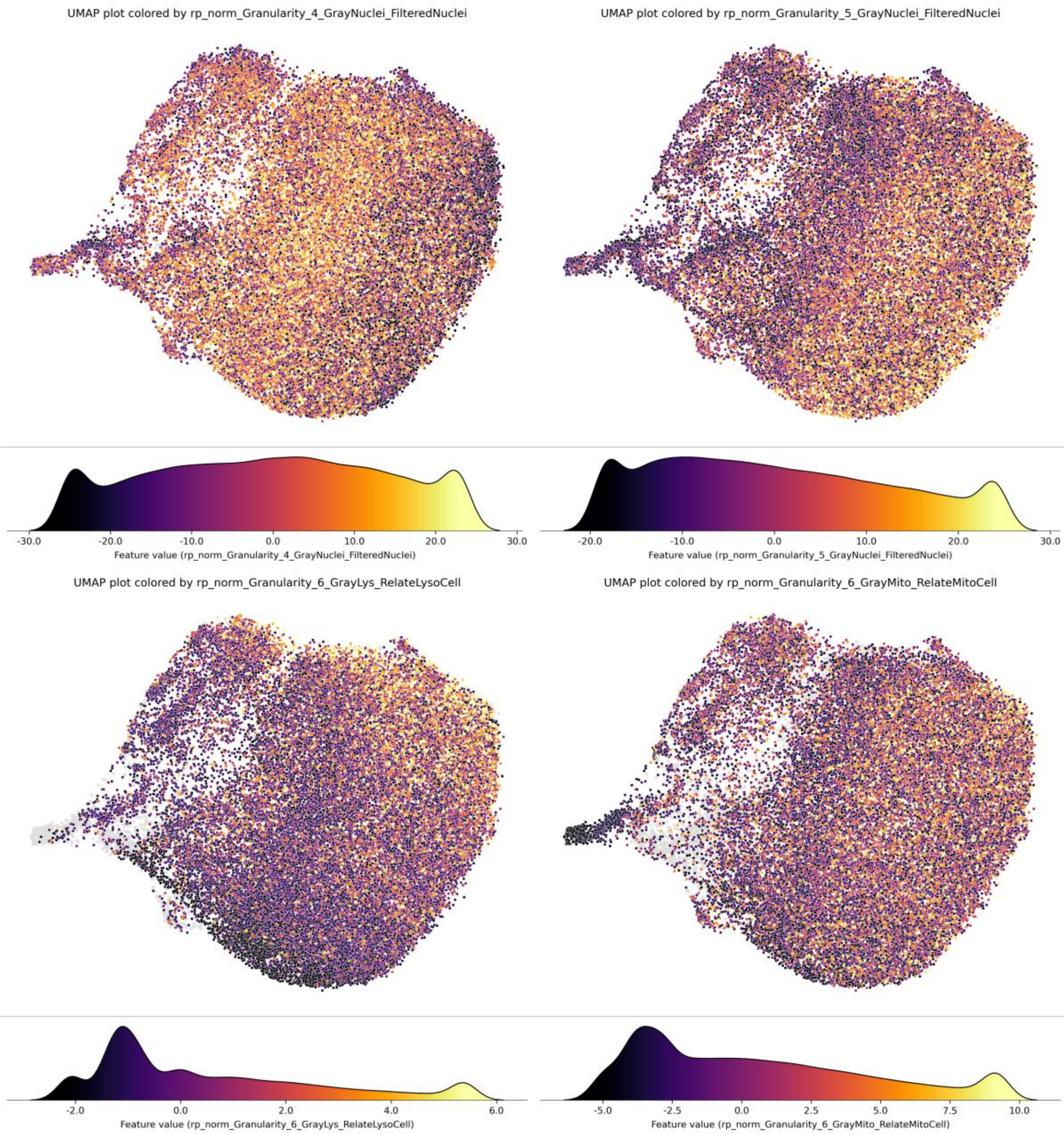
UMAP plot colored by rp_norm_Granularity_4_GrayNuclei_FilteredNuclei
-30.0
-20.0
-10.0
0.0
10.0
20.0
30.0
Feature value (rp_norm_Granularity_4_GrayNuclei_FilteredNuclei)
UMAP plot colored by rp_norm_Granularity_5_GrayNuclei_FilteredNuclei
-20.0
-10.0
0.0
10.0
20.0
30.0
Feature value (rp_norm_Granularity_5_GrayNuclei_FilteredNuclei)
UMAP plot colored by rp_norm_Granularity_6_GrayLys_RelateLysoCell
-2.0
0.0
2.0
4.0
6.0
Feature value (rp_norm_Granularity_6_GrayLys_RelateLysoCell)
UMAP plot colored by rp_norm_Granularity_6_GrayMito_RelateMitoCell
-5.0
-2.5
0.0
2.5
5.0
7.5
10.0
Feature value (rp_norm_Granularity_6_GrayMito_RelateMitoCell)

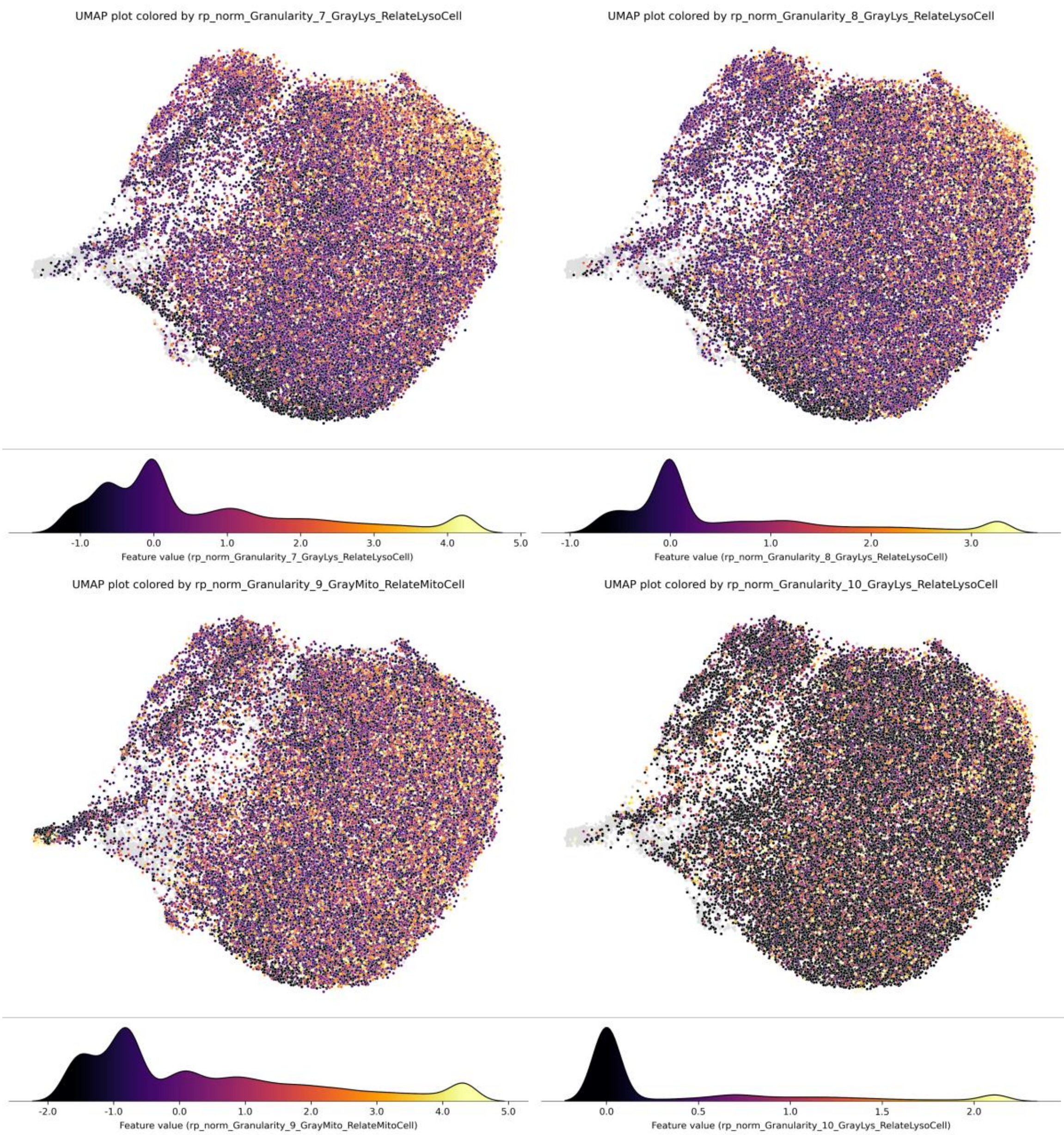
UMAP plot colored by rp_norm_Granularity_7_GrayLys_RelateLysoCell
-1.0 0.0 1.0 2.0 3.0 4.0 5.0
Feature value (rp_norm_Granularity_7_GrayLys_RelateLysoCell)
UMAP plot colored by rp_norm_Granularity_8_GrayLys_RelateLysoCell
-1.0 0.0 1.0 2.0 3.0
Feature value (rp_norm_Granularity_8_GrayLys_RelateLysoCell)
UMAP plot colored by rp_norm_Granularity_9_GrayMito_RelateMitoCell
-2.0 -1.0 0.0 1.0 2.0 3.0 4.0 5.0
Feature value (rp_norm_Granularity_9_GrayMito_RelateMitoCell)
UMAP plot colored by rp_norm_Granularity_10_GrayLys_RelateLysoCell
0.0 0.5 1.0 1.5 2.0
Feature value (rp_norm_Granularity_10_GrayLys_RelateLysoCell)

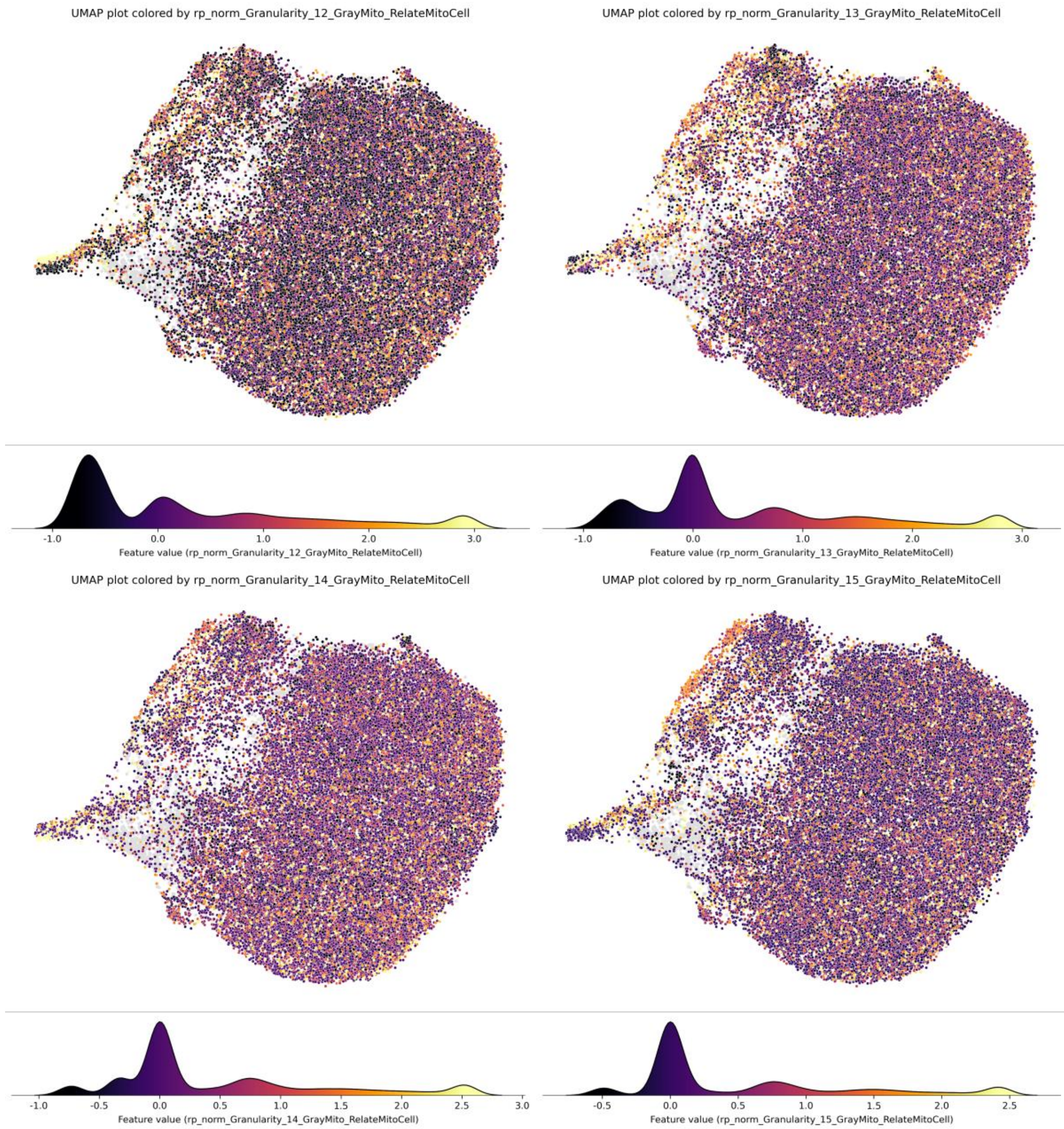
UMAP plot colored by rp_norm_Granularity_12_GrayMito_RelateMitoCell
-1.0
0.0
1.0
2.0
3.0
Feature value (rp_norm_Granularity_12_GrayMito_RelateMitoCell)
UMAP plot colored by rp_norm_Granularity_13_GrayMito_RelateMitoCell
-1.0
0.0
1.0
2.0
3.0
Feature value (rp_norm_Granularity_13_GrayMito_RelateMitoCell)
UMAP plot colored by rp_norm_Granularity_14_GrayMito_RelateMitoCell
-1.0
-0.5
0.0
0.5
1.0
1.5
2.0
2.5
3.0
Feature value (rp_norm_Granularity_14_GrayMito_RelateMitoCell)
UMAP plot colored by rp_norm_Granularity_15_GrayMito_RelateMitoCell
-0.5
0.0
0.5
1.0
1.5
2.0
2.5
Feature value (rp_norm_Granularity_15_GrayMito_RelateMitoCell)

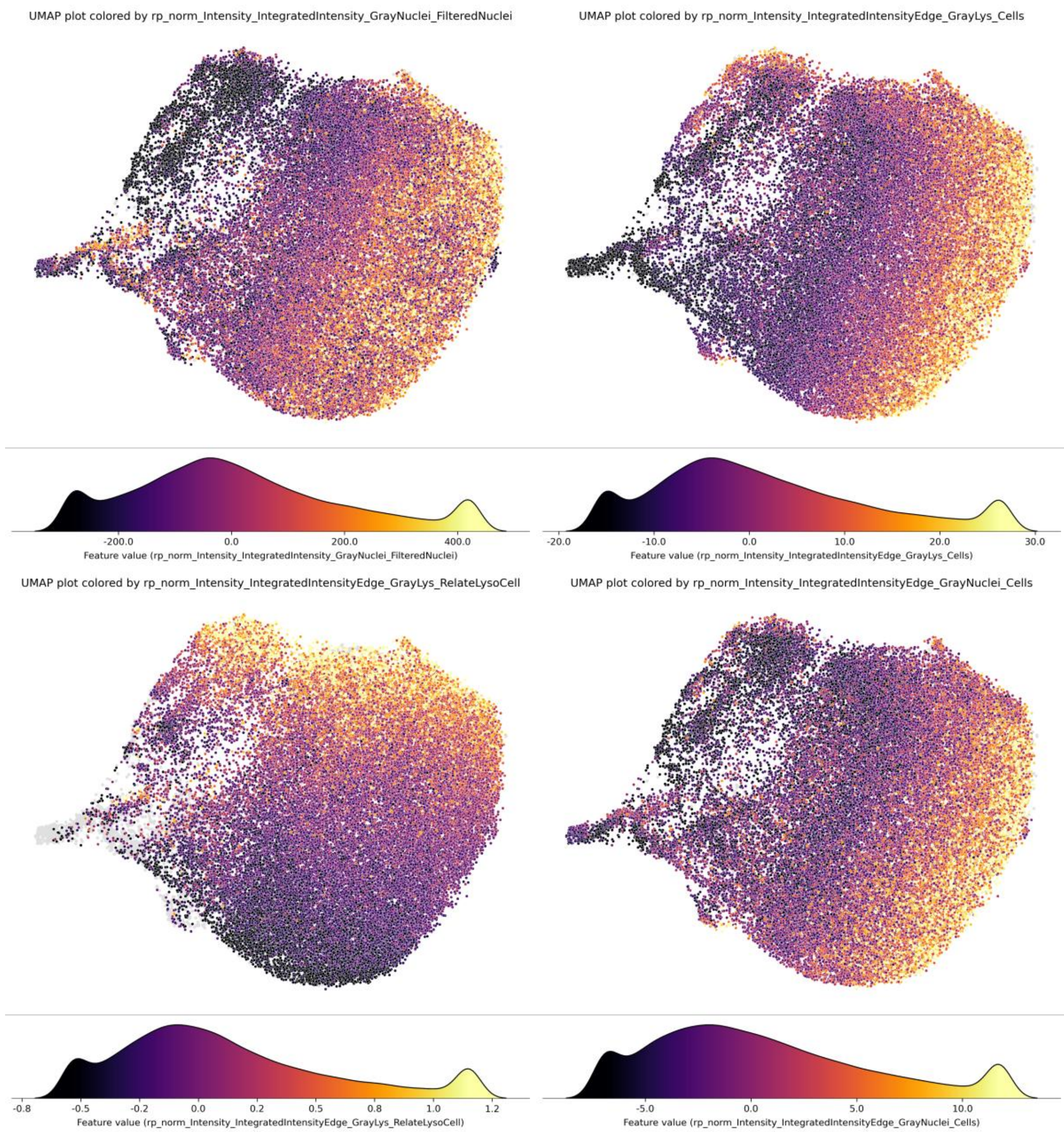
UMAP plot colored by rp_norm_Intensity_IntegratedIntensity_GrayNuclei_FilteredNuclei
-200.0
0.0
200.0
400.0
Feature value (rp_norm_Intensity_IntegratedIntensity_GrayNuclei_FilteredNuclei)
UMAP plot colored by rp_norm_Intensity_IntegratedIntensityEdge_GrayLys_Cells
-20.0
-10.0
0.0
10.0
20.0
30.0
Feature value (rp_norm_Intensity_IntegratedIntensityEdge_GrayLys_Cells)
UMAP plot colored by rp_norm_Intensity_IntegratedIntensityEdge_GrayLys_RelateLysoCell
-0.8
-0.5
-0.2
0.0
0.2
0.5
0.8
1.0
1.2
Feature value (rp_norm_Intensity_IntegratedIntensityEdge_GrayLys_RelateLysoCell)
UMAP plot colored by rp_norm_Intensity_IntegratedIntensityEdge_GrayNuclei_Cells
-5.0
0.0
5.0
10.0
Feature value (rp_norm_Intensity_IntegratedIntensityEdge_GrayNuclei_Cells)

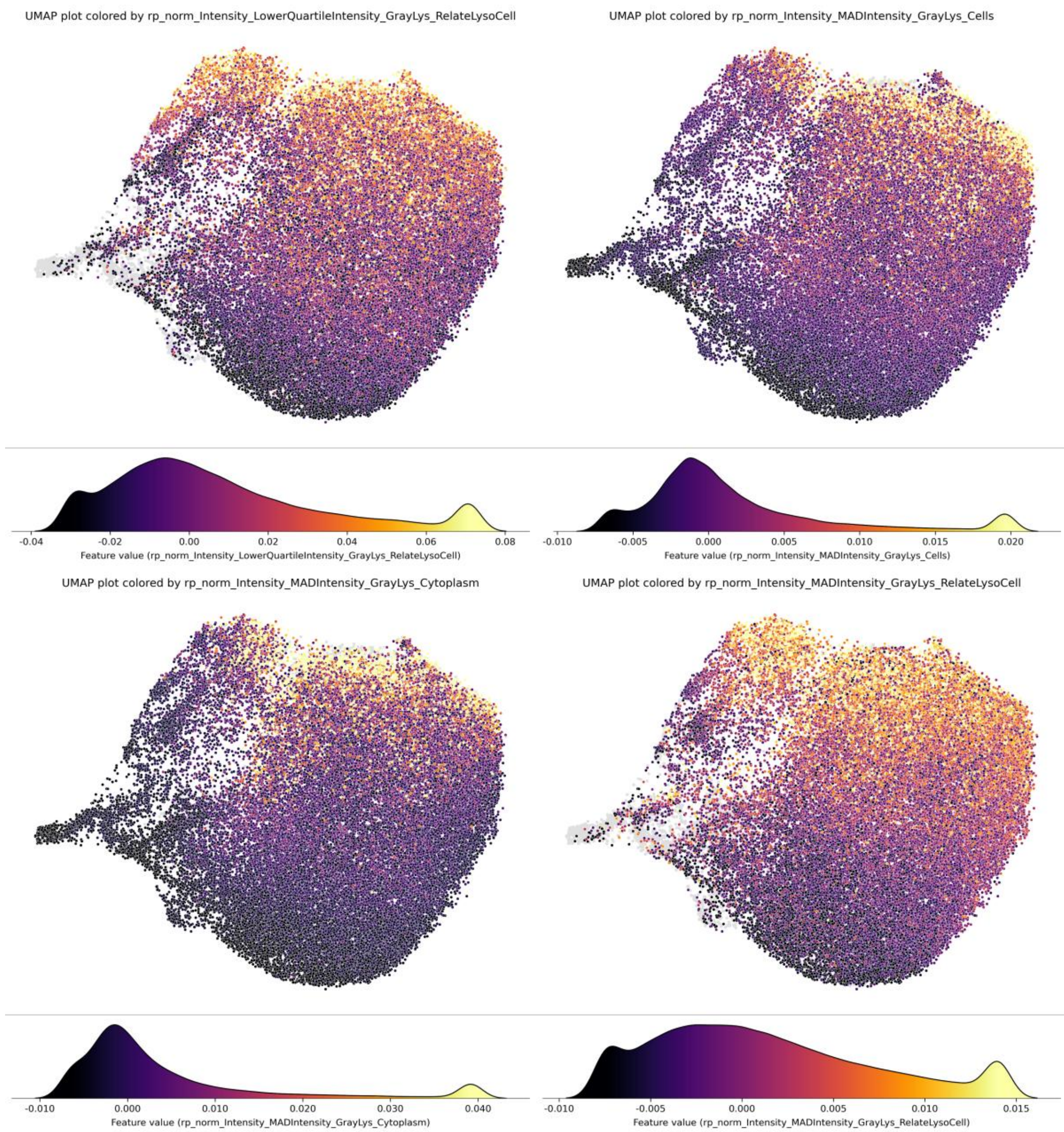

UMAP plot colored by rp_norm_Intensity_LowerQuartileIntensity_GrayLys_RelateLysoCell
UMAP plot colored by rp_norm_Intensity_MADIntensity_GrayLys_Cells
-0.04 -0.02 0.00 0.02 0.04 0.06 0.08
Feature value (rp_norm_Intensity_LowerQuartileIntensity_GrayLys_RelateLysoCell)
-0.010 -0.005 0.000 0.005 0.010 0.015 0.020
Feature value (rp_norm_Intensity_MADIntensity_GrayLys_Cells)
UMAP plot colored by rp_norm_Intensity_MADIntensity_GrayLys_Cytoplasm
UMAP plot colored by rp_norm_Intensity_MADIntensity_GrayLys_RelateLysoCell
-0.010 0.000 0.010 0.020 0.030 0.040
Feature value (rp_norm_Intensity_MADIntensity_GrayLys_Cytoplasm)
-0.010 -0.005 0.000 0.005 0.010 0.015
Feature value (rp_norm_Intensity_MADIntensity_GrayLys_RelateLysoCell)

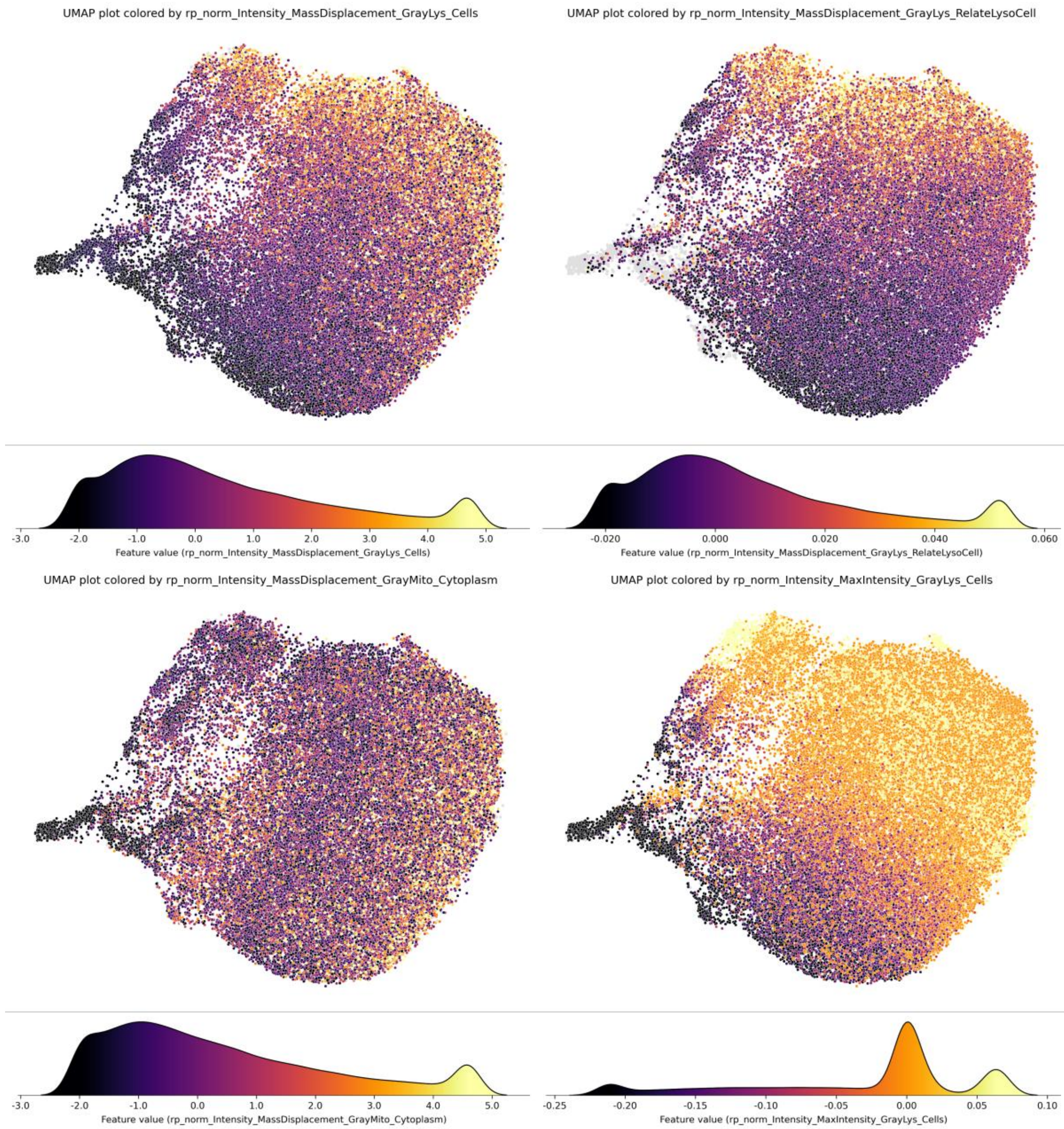
UMAP plot colored by rp_norm_Intensity_MassDisplacement_GrayLys_Cells
UMAP plot colored by rp_norm_Intensity_MassDisplacement_GrayLys_RelateLysoCell
-3.0 -2.0 -1.0 0.0 1.0 2.0 3.0 4.0 5.0
Feature value (rp_norm_Intensity_MassDisplacement_GrayLys_Cells)
-0.020 0.000 0.020 0.040 0.060
Feature value (rp_norm_Intensity_MassDisplacement_GrayLys_RelateLysoCell)
UMAP plot colored by rp_norm_Intensity_MassDisplacement_GrayMito_Cytoplasm
UMAP plot colored by rp_norm_Intensity_MaxIntensity_GrayLys_Cells
-3.0 -2.0 -1.0 0.0 1.0 2.0 3.0 4.0 5.0
Feature value (rp_norm_Intensity_MassDisplacement_GrayMito_Cytoplasm)
-0.25 -0.20 -0.15 -0.10 -0.05 0.00 0.05 0.10
Feature value (rp_norm_Intensity_MaxIntensity_GrayLys_Cells)

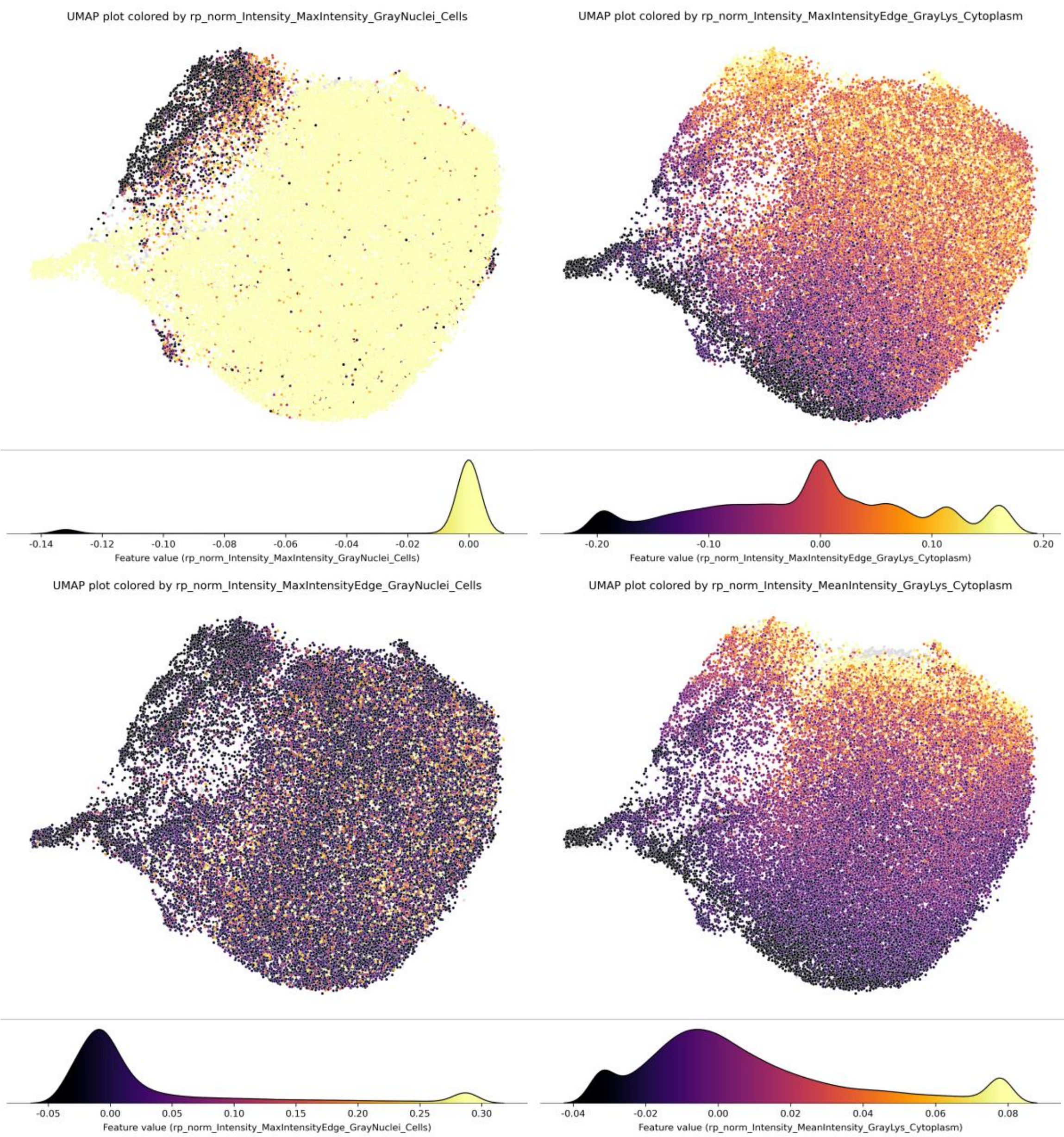
UMAP plot colored by rp_norm_Intensity_MaxIntensity_GrayNuclei_Cells
-0.14 -0.12 -0.10 -0.08 -0.06 -0.04 -0.02 0.00
Feature value (rp_norm_Intensity_MaxIntensity_GrayNuclei_Cells)
UMAP plot colored by rp_norm_Intensity_MaxIntensityEdge_GrayLys_Cytoplasm
-0.20 -0.10 0.00 0.10 0.20
Feature value (rp_norm_Intensity_MaxIntensityEdge_GrayLys_Cytoplasm)
UMAP plot colored by rp_norm_Intensity_MaxIntensityEdge_GrayNuclei_Cells
-0.05 0.00 0.05 0.10 0.15 0.20 0.25 0.30
Feature value (rp_norm_Intensity_MaxIntensityEdge_GrayNuclei_Cells)
UMAP plot colored by rp_norm_Intensity_MeanIntensity_GrayLys_Cytoplasm
-0.04 -0.02 0.00 0.02 0.04 0.06 0.08
Feature value (rp_norm_Intensity_MeanIntensity_GrayLys_Cytoplasm)

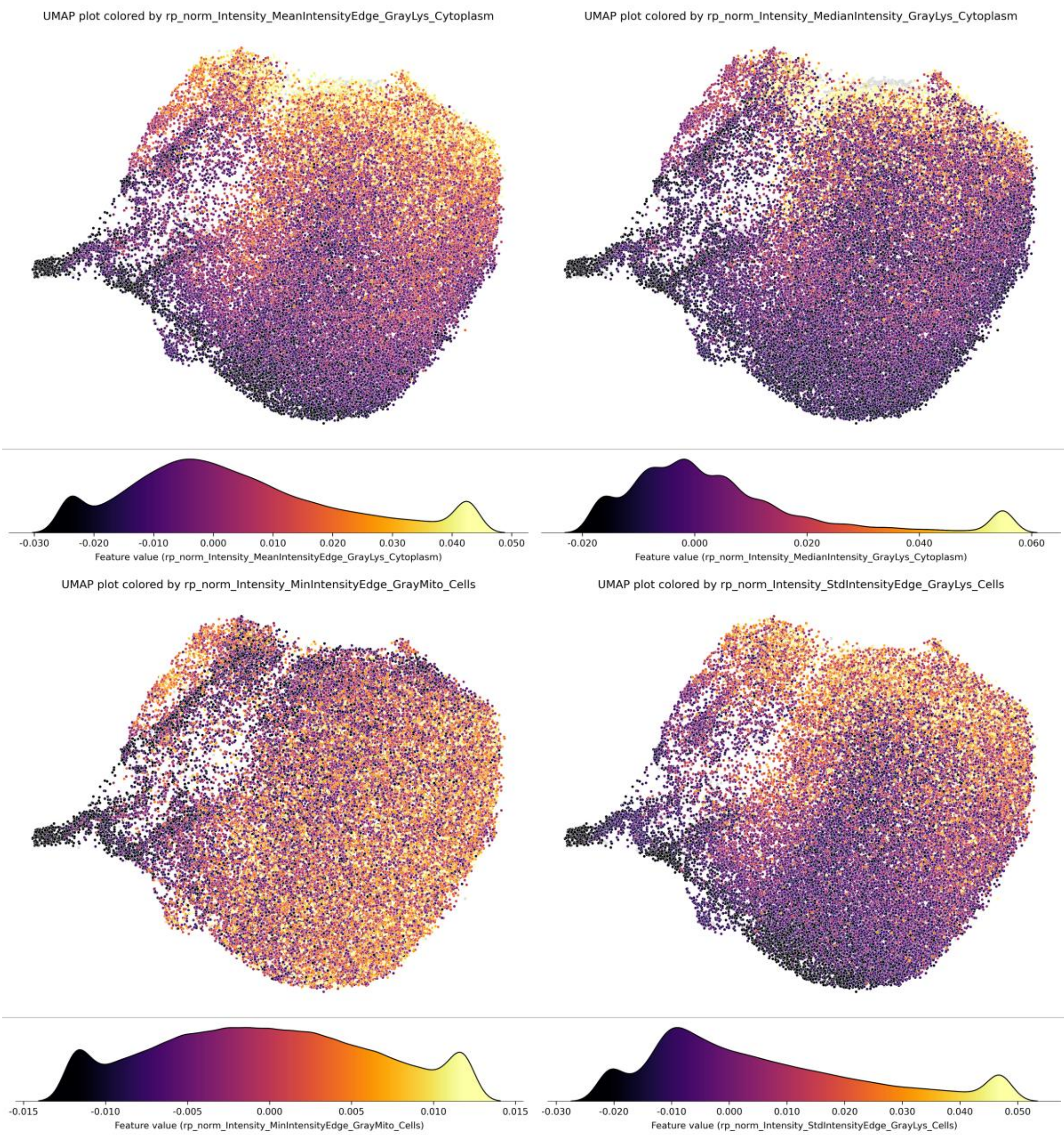
UMAP plot colored by rp_norm_Intensity_MeanIntensityEdge_GrayLys_Cytoplasm
-0.030 -0.020 -0.010 0.000 0.010 0.020 0.030 0.040 0.050
Feature value (rp_norm_Intensity_MeanIntensityEdge_GrayLys_Cytoplasm)
UMAP plot colored by rp_norm_Intensity_MedianIntensity_GrayLys_Cytoplasm
-0.020 0.000 0.020 0.040 0.060
Feature value (rp_norm_Intensity_MedianIntensity_GrayLys_Cytoplasm)
UMAP plot colored by rp_norm_Intensity_MinIntensityEdge_GrayMito_Cells
-0.015 -0.010 -0.005 0.000 0.005 0.010 0.015
Feature value (rp_norm_Intensity_MinIntensityEdge_GrayMito_Cells)
UMAP plot colored by rp_norm_Intensity_StdIntensityEdge_GrayLys_Cells
-0.030 -0.020 -0.010 0.000 0.010 0.020 0.030 0.040 0.050
Feature value (rp_norm_Intensity_StdIntensityEdge_GrayLys_Cells)

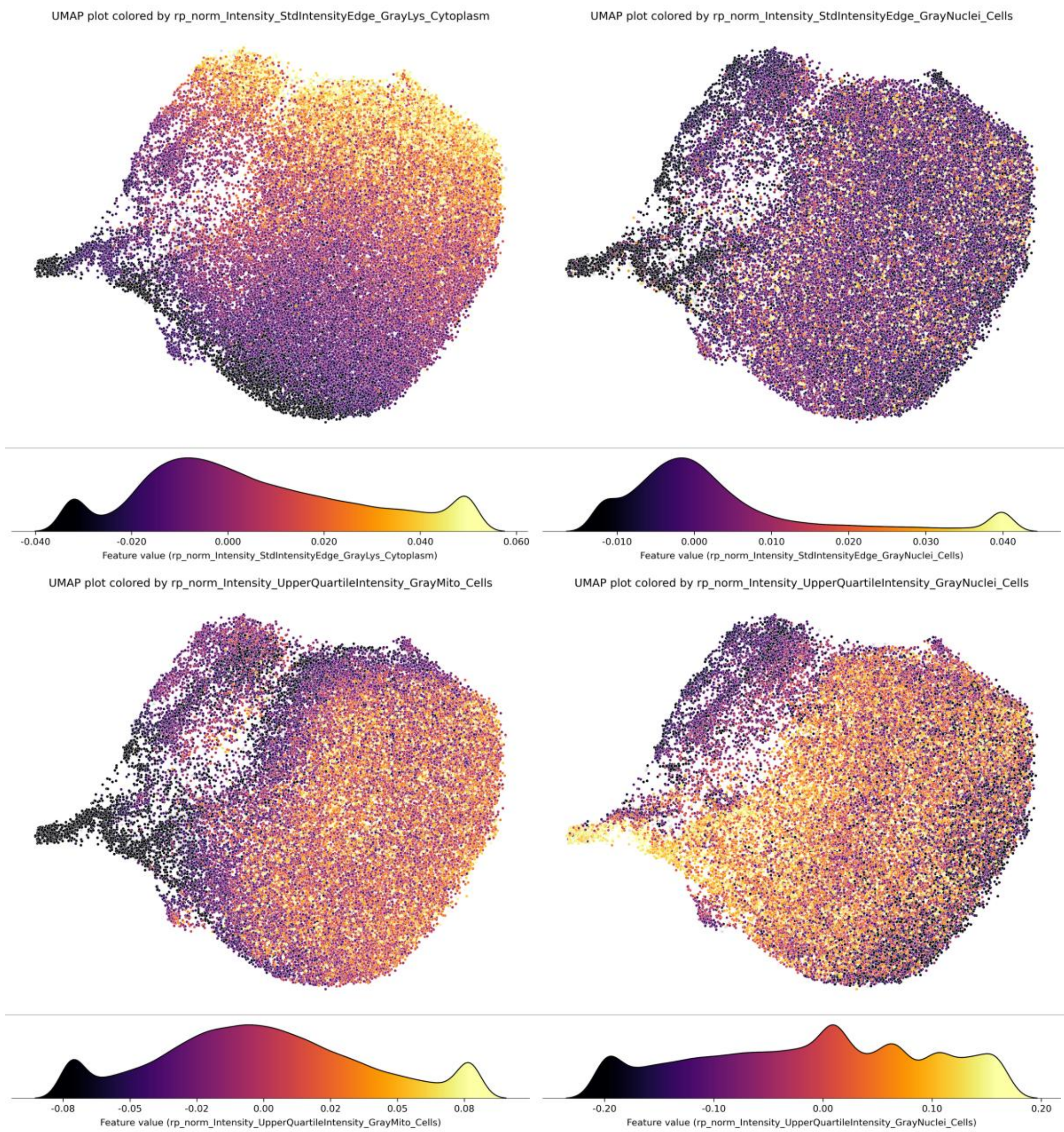
UMAP plot colored by rp_norm_Intensity_StdIntensityEdge_GrayLys_Cytoplasm
-0.040 -0.020 0.000 0.020 0.040 0.060
Feature value (rp_norm_Intensity_StdIntensityEdge_GrayLys_Cytoplasm)
UMAP plot colored by rp_norm_Intensity_StdIntensityEdge_GrayNuclei_Cells
-0.010 0.000 0.010 0.020 0.030 0.040
Feature value (rp_norm_Intensity_StdIntensityEdge_GrayNuclei_Cells)
UMAP plot colored by rp_norm_Intensity_UpperQuartileIntensity_GrayMito_Cells
-0.08 -0.05 -0.02 0.00 0.02 0.05 0.08
Feature value (rp_norm_Intensity_UpperQuartileIntensity_GrayMito_Cells)
UMAP plot colored by rp_norm_Intensity_UpperQuartileIntensity_GrayNuclei_Cells
-0.20 -0.10 0.00 0.10 0.20
Feature value (rp_norm_Intensity_UpperQuartileIntensity_GrayNuclei_Cells)

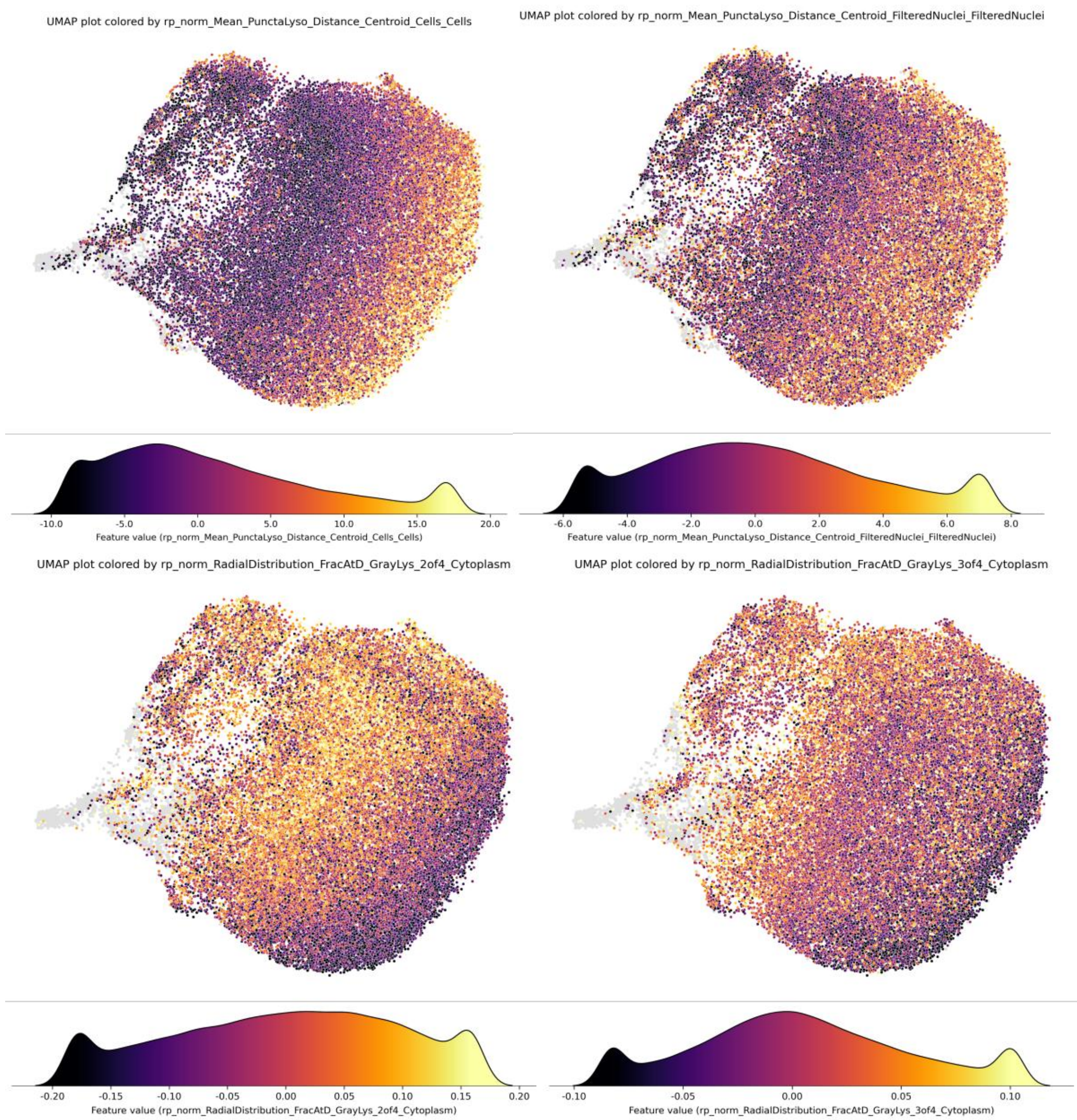
UMAP plot colored by rp_norm_Mean_PunctaLyso_Distance_Centroid_Cells_Cells
-10.0 -5.0 0.0 5.0 10.0 15.0 20.0
Feature value (rp_norm_Mean_PunctaLyso_Distance_Centroid_Cells_Cells)
UMAP plot colored by rp_norm_Mean_PunctaLyso_Distance_Centroid_FilteredNuclei_FilteredNuclei
-6.0 -4.0 -2.0 0.0 2.0 4.0 6.0 8.0
Feature value (rp_norm_Mean_PunctaLyso_Distance_Centroid_FilteredNuclei_FilteredNuclei)
UMAP plot colored by rp_norm_RadialDistribution_FracAtD_GrayLys_2of4_Cytoplasm
-0.20 -0.15 -0.10 -0.05 0.00 0.05 0.10 0.15 0.20
Feature value (rp_norm_RadialDistribution_FracAtD_GrayLys_2of4_Cytoplasm)
UMAP plot colored by rp_norm_RadialDistribution_FracAtD_GrayLys_3of4_Cytoplasm
-0.10 -0.05 0.00 0.05 0.10
Feature value (rp_norm_RadialDistribution_FracAtD_GrayLys_3of4_Cytoplasm)

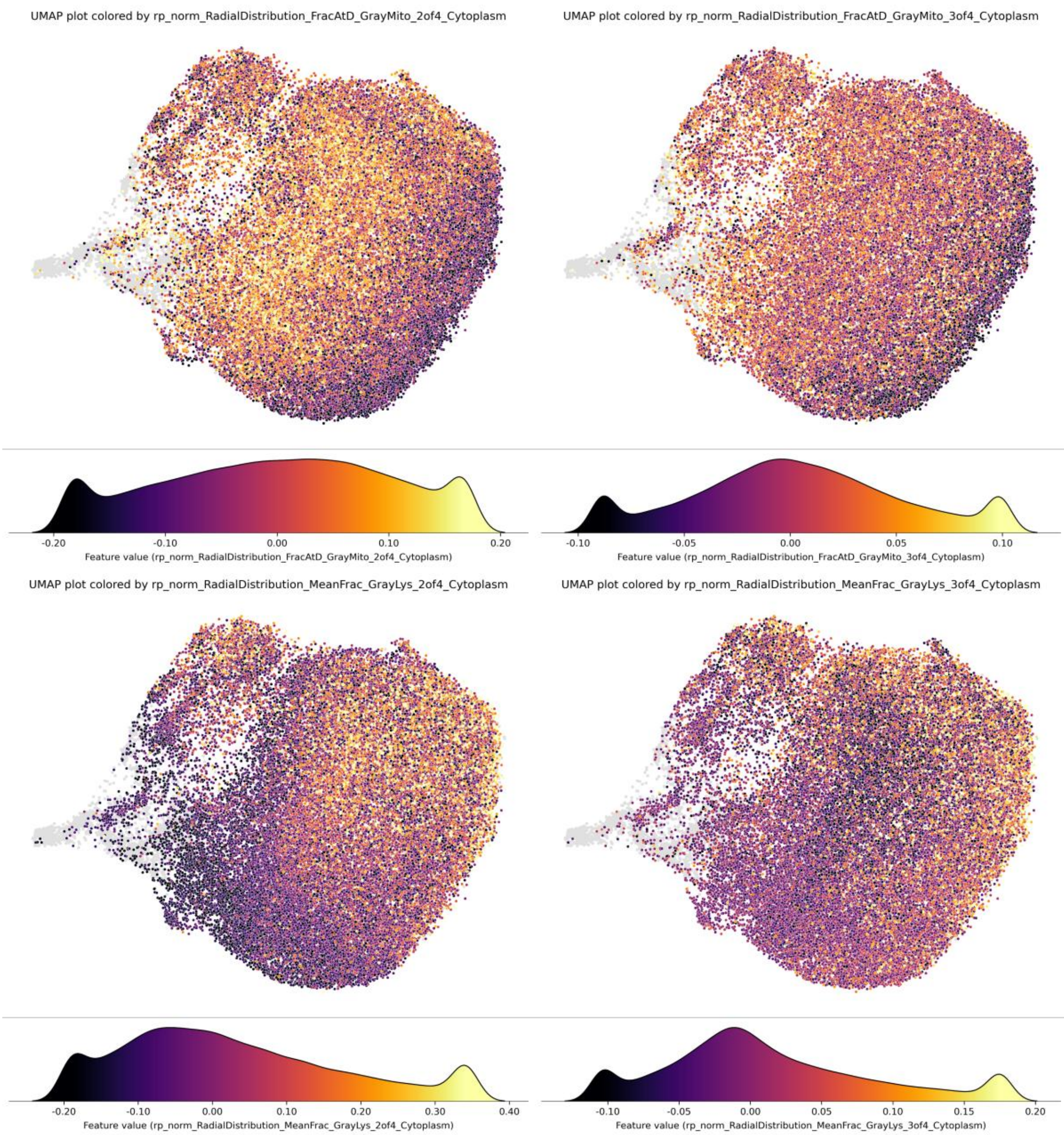
UMAP plot colored by rp_norm_RadialDistribution_FracAtD_GrayMito_2of4_Cytoplasm
-0.20 -0.10 0.00 0.10 0.20
Feature value (rp_norm_RadialDistribution_FracAtD_GrayMito_2of4_Cytoplasm)
UMAP plot colored by rp_norm_RadialDistribution_FracAtD_GrayMito_3of4_Cytoplasm
-0.10 -0.05 0.00 0.05 0.10
Feature value (rp_norm_RadialDistribution_FracAtD_GrayMito_3of4_Cytoplasm)
UMAP plot colored by rp_norm_RadialDistribution_MeanFrac_GrayLys_2of4_Cytoplasm
-0.20 -0.10 0.00 0.10 0.20 0.30 0.40
Feature value (rp_norm_RadialDistribution_MeanFrac_GrayLys_2of4_Cytoplasm)
UMAP plot colored by rp_norm_RadialDistribution_MeanFrac_GrayLys_3of4_Cytoplasm
-0.10 -0.05 0.00 0.05 0.10 0.15 0.20
Feature value (rp_norm_RadialDistribution_MeanFrac_GrayLys_3of4_Cytoplasm)

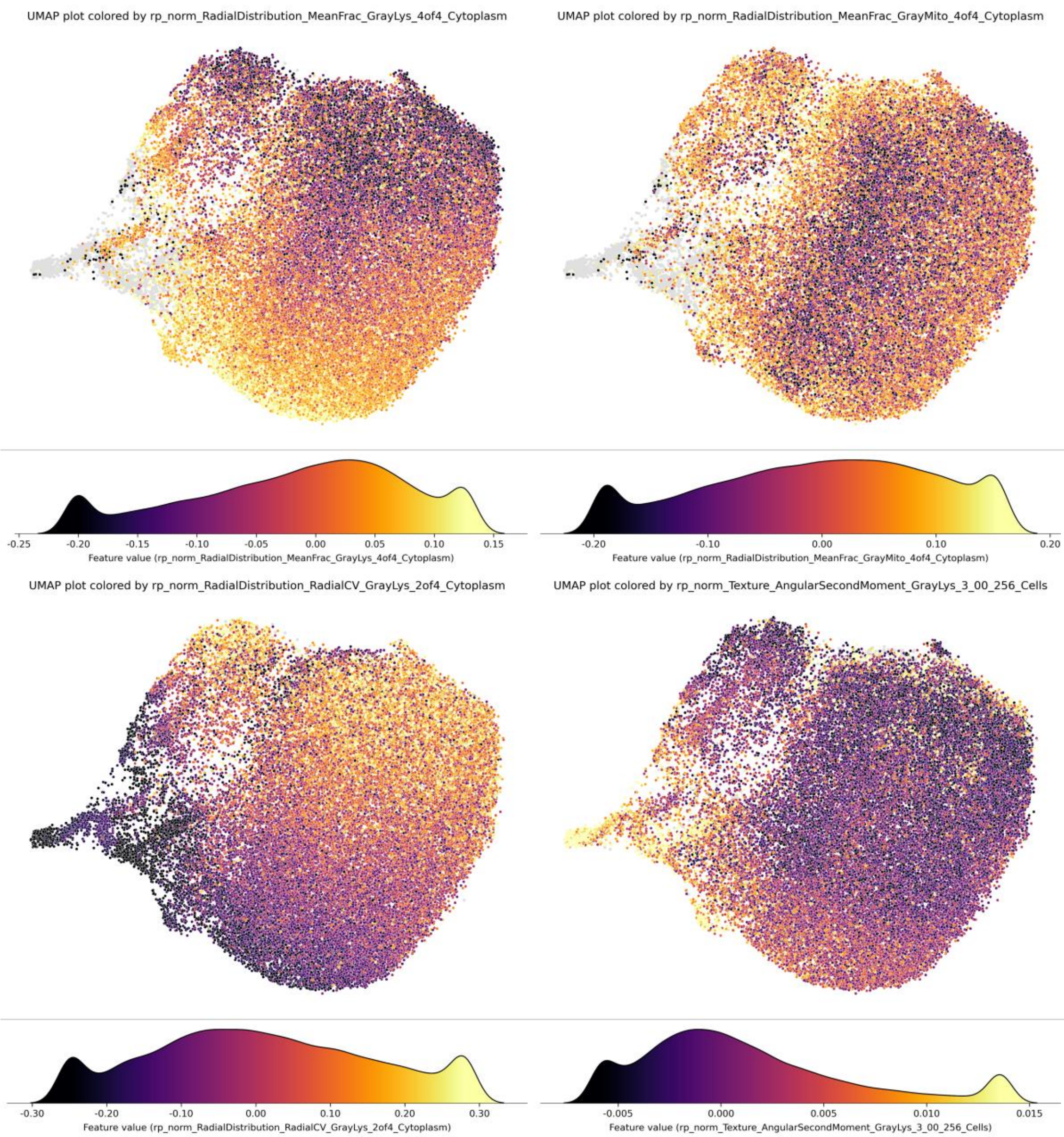
UMAP plot colored by rp_norm_RadialDistribution_MeanFrac_GrayLys_4of4_Cytoplasm
UMAP plot colored by rp_norm_RadialDistribution_MeanFrac_GrayMito_4of4_Cytoplasm
-0.25 -0.20 -0.15 -0.10 -0.05 0.00 0.05 0.10 0.15
Feature value (rp_norm_RadialDistribution_MeanFrac_GrayLys_4of4_Cytoplasm)
-0.20 -0.10 0.00 0.10 0.20
Feature value (rp_norm_RadialDistribution_MeanFrac_GrayMito_4of4_Cytoplasm)
UMAP plot colored by rp_norm_RadialDistribution_RadialCV_GrayLys_2of4_Cytoplasm
UMAP plot colored by rp_norm_Texture_AngularSecondMoment_GrayLys_3_00_256_Cells
-0.30 -0.20 -0.10 0.00 0.10 0.20 0.30
Feature value (rp_norm_RadialDistribution_RadialCV_GrayLys_2of4_Cytoplasm)
-0.005 0.000 0.005 0.010 0.015
Feature value (rp_norm_Texture_AngularSecondMoment_GrayLys_3_00_256_Cells)

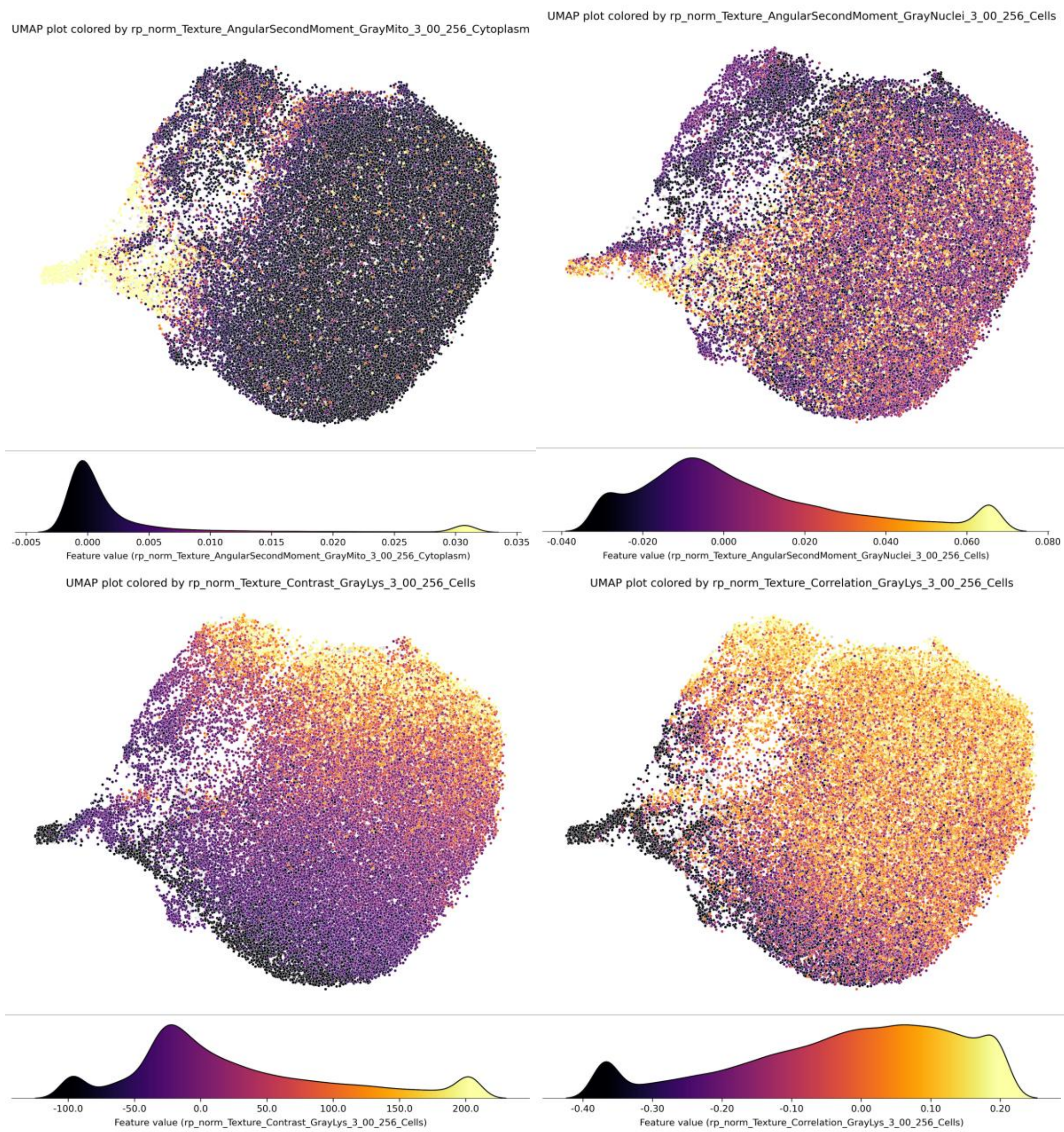
UMAP plot colored by rp_norm_Texture_AngularSecondMoment_GrayMito_3_00_256_Cytoplasm
-0.005 0.000 0.005 0.010 0.015 0.020 0.025 0.030 0.035
Feature value (rp_norm_Texture_AngularSecondMoment_GrayMito_3_00_256_Cytoplasm)
UMAP plot colored by rp_norm_Texture_AngularSecondMoment_GrayNuclei_3_00_256_Cells
-0.040 -0.020 0.000 0.020 0.040 0.060 0.080
Feature value (rp_norm_Texture_AngularSecondMoment_GrayNuclei_3_00_256_Cells)
UMAP plot colored by rp_norm_Texture_Contrast_GrayLys_3_00_256_Cells
-100.0 -50.0 0.0 50.0 100.0 150.0 200.0
Feature value (rp_norm_Texture_Contrast_GrayLys_3_00_256_Cells)
UMAP plot colored by rp_norm_Texture_Correlation_GrayLys_3_00_256_Cells
-0.40 -0.30 -0.20 -0.10 0.00 0.10 0.20
Feature value (rp_norm_Texture_Correlation_GrayLys_3_00_256_Cells)

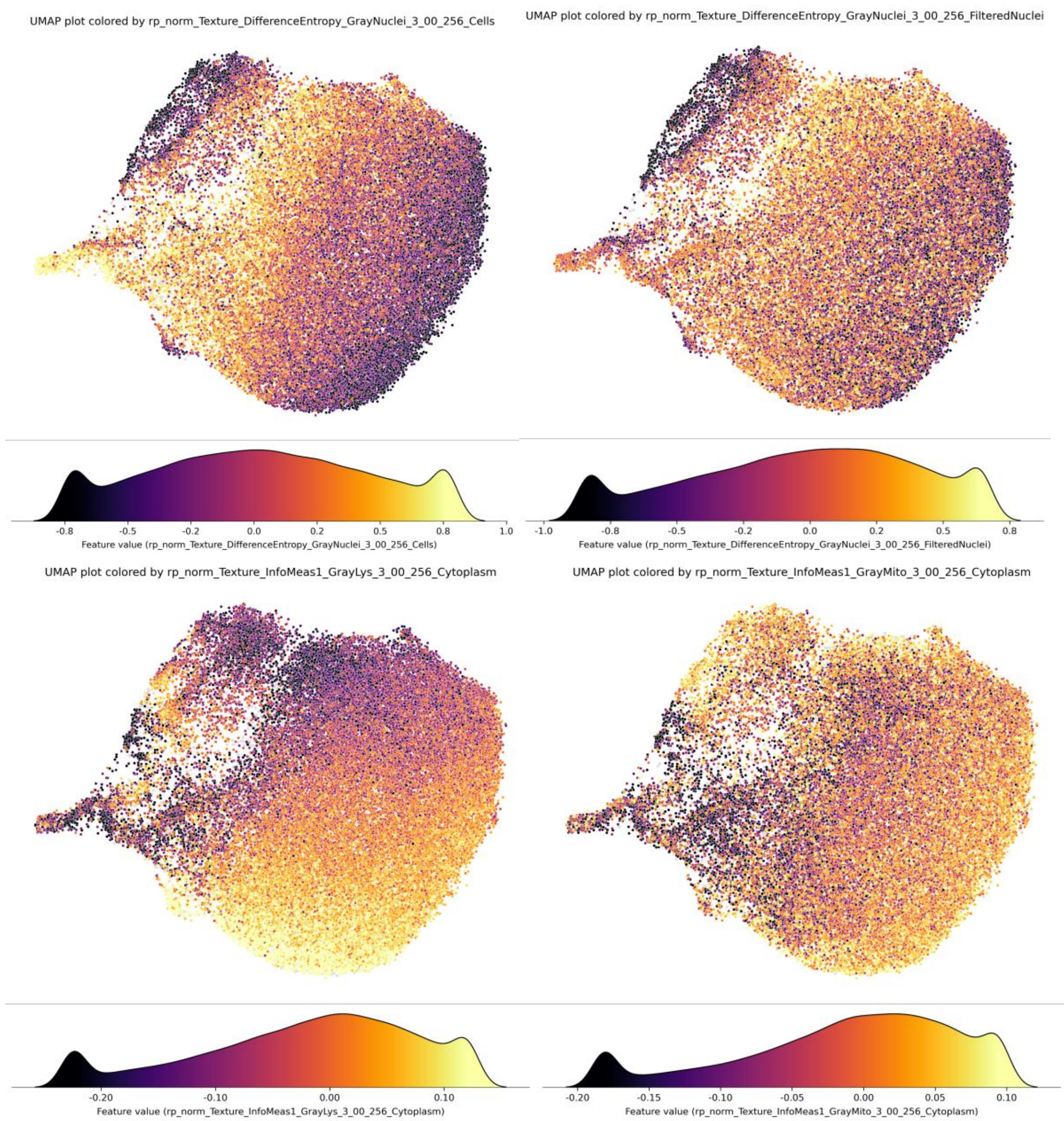
UMAP plot colored by rp_norm_Texture_DifferenceEntropy_GrayNuclei_3_00_256_Cells
UMAP plot colored by rp_norm_Texture_DifferenceEntropy_GrayNuclei_3_00_256_FilteredNuclei
Feature value (rp_norm_Texture_DifferenceEntropy_GrayNuclei_3_00_256_Cells)
Feature value (rp_norm_Texture_DifferenceEntropy_GrayNuclei_3_00_256_FilteredNuclei)
UMAP plot colored by rp_norm_Texture_InfoMeas1_GrayLys_3_00_256_Cytoplasm
UMAP plot colored by rp_norm_Texture_InfoMeas1_GrayMito_3_00_256_Cytoplasm
Feature value (rp_norm_Texture_InfoMeas1_GrayLys_3_00_256_Cytoplasm)
Feature value (rp_norm_Texture_InfoMeas1_GrayMito_3_00_256_Cytoplasm)

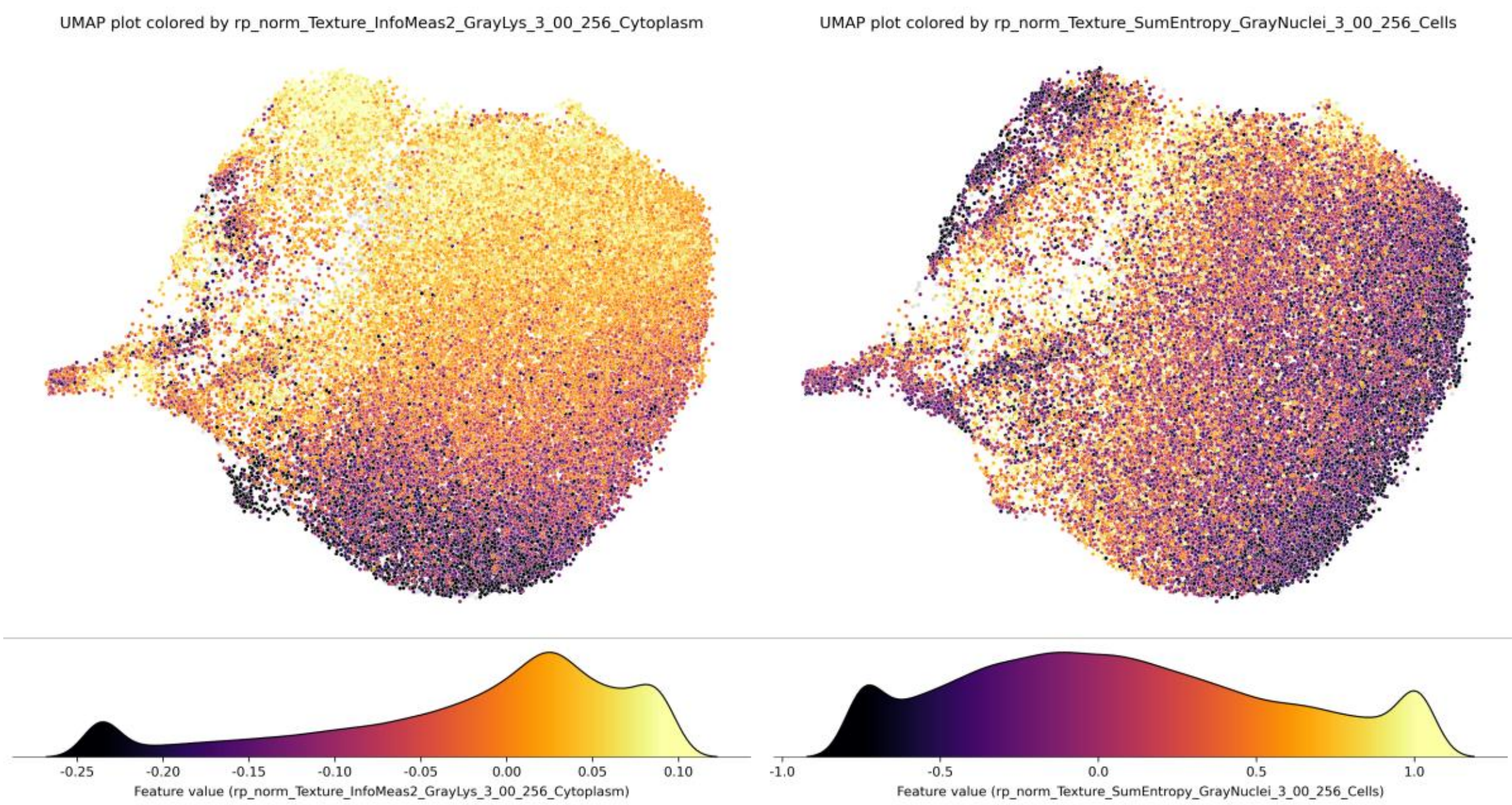

Figures S2: All selected 94 features colored on a UMAP embedding for Day 5 of the exposure. Each feature has a distribution plot of standardized measurements (x-axis has arbitrary units). Gray values (points) indicate missing data.

S3: Image Quality Classifier Performance Summary

Table S3: Image Quality Control Classifier Performance

Classifier	Total Images	Clean Images	Problematic Images	Problem Rate (%)	Mean Confidence	High Confidence Rate (>80%)
**Artifacts**	4,806	4,744	62	1.3%	83.9% ± 7.0%	73.3%
**Empty**	4,806	4,737	69	1.4%	98.0% ± 6.5%	96.7%
**Oversaturation**	4,806	4,752	54	1.1%	89.2% ± 5.5%	94.2%

Each Random Forest classifier was trained with 100 estimators using gini criterion, square root feature sampling, and bootstrap aggregation (all other parameters at Scikit-learn 1.3.2 defaults) on 148 CellProfiler-derived image quality features (MeasureImageQuality module). Performance metrics were evaluated on the complete dataset of 4806 images.

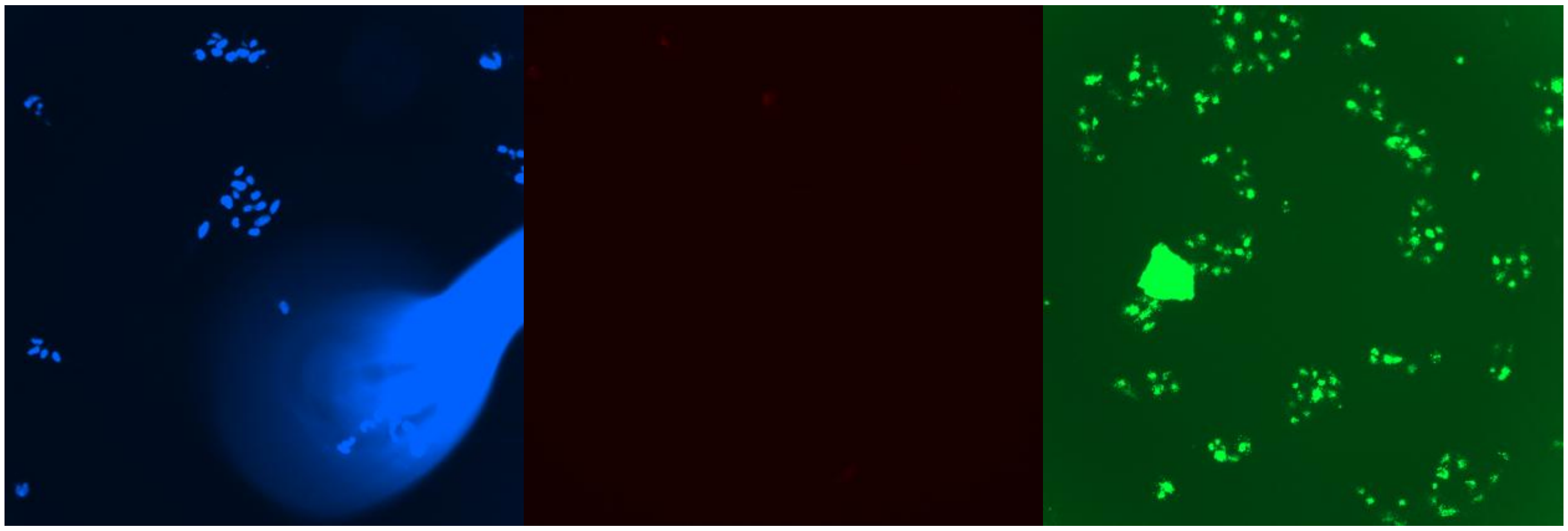

Figure S3: Examples of images removed, tagged with Artifacts, Empty, Oversaturation, consecutively.

S4: Poor segmentations artefacts

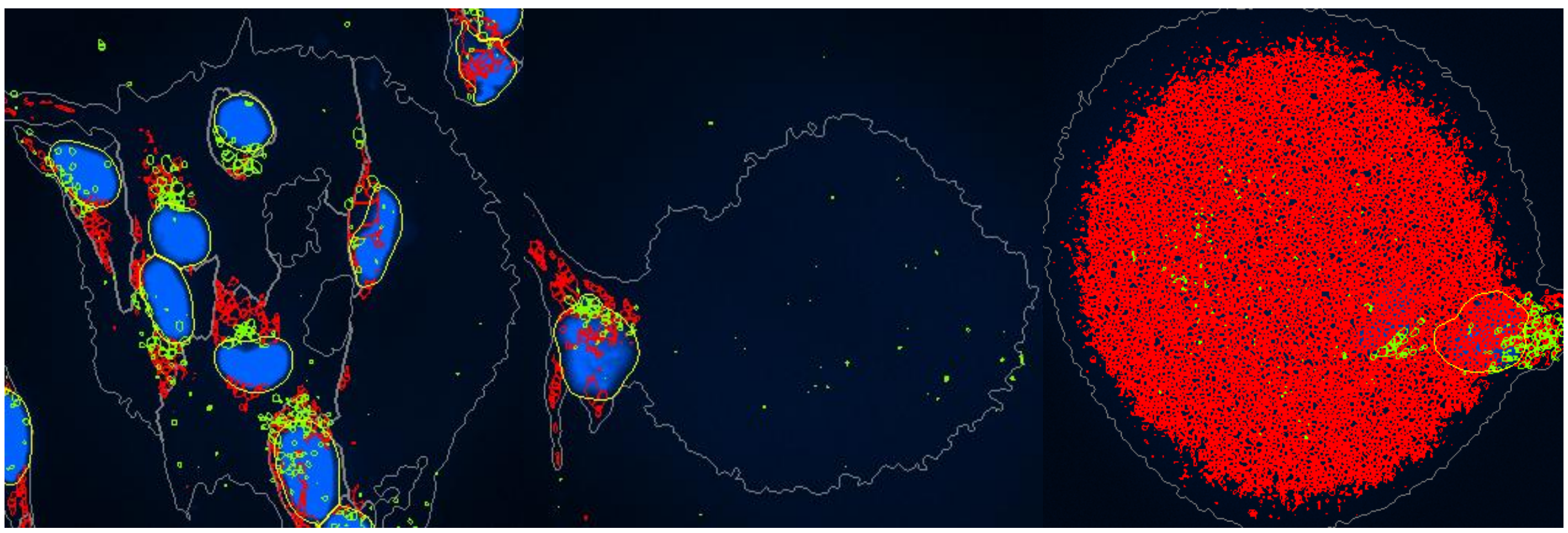

Figure S4.1: Examples of incorrectly segmented cells producing extreme values. We observe oversegmentation (thin white line) capturing noisy signal, and false identification of mitochondrial signal (red blob).

Incorrect segmentation in this dataset arose primarily from two sources: intensity variability across images within the dataset, which can cause classical thresholding algorithms to over- or under-segment cell boundaries, and occasional artefacts not captured by the image-level QC classifiers that introduced atypical intensity or morphology values at the cell level. Both sources are inherent limitations of threshold-based segmentation pipelines applied to heterogeneous biological samples.

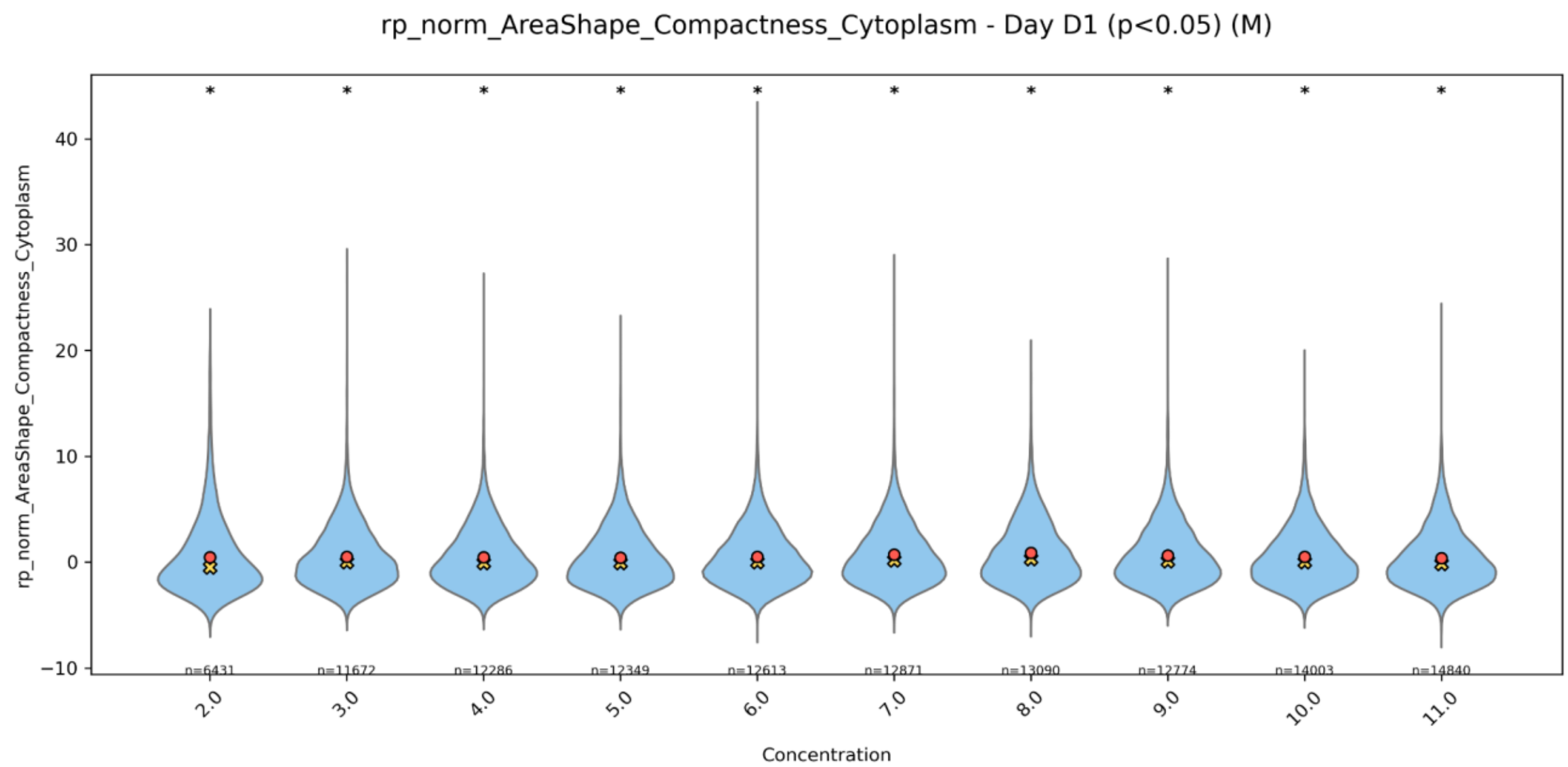

Figure S4.2: Example of violin plots for data (normalized feature AreaShape_Compactness_Cytoplasm) where outlier removal **wasn't** performed.

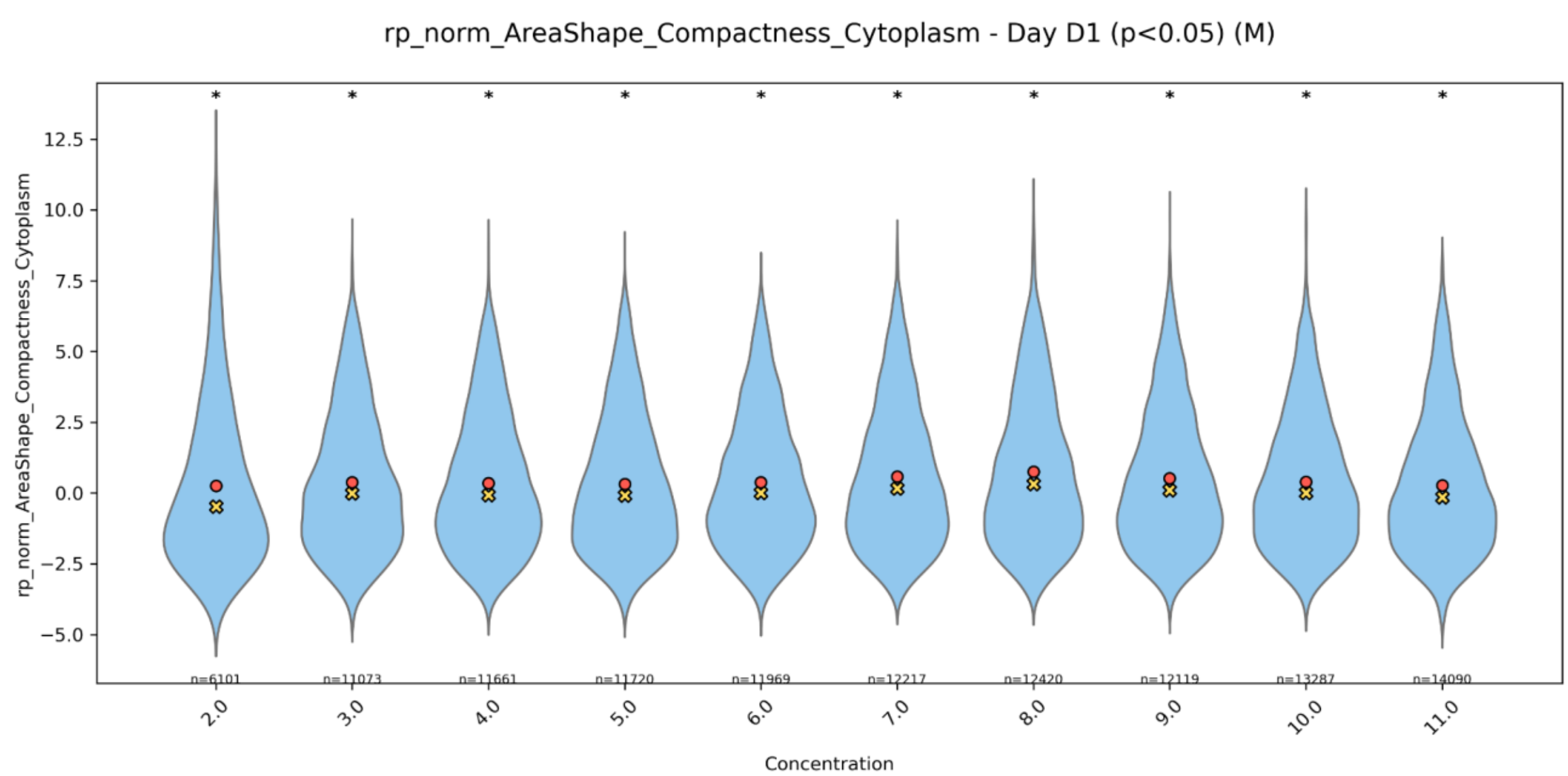

Figure S4.3: Example of violin plots for data (normalized feature AreaShape_Compactness_Cytoplasm) where outlier removal **was** performed.

Concentration X-axis: 2.0 - is the highest exposure, where 10.0 is the lowest exposure concentration; 11.0 being control. Violin plots were generated with the following code: https://github.com/NIB-SI/HCS-proc/blob/main/scripts/alternative/violin_plots_per_feature/violin_plots_per_feature.py

All segmented images and violin plots for all the features with or without outlier removal are available at: https://doi.org/10.5281/zenodo.17951792.

S5: Z-score standardization comparison

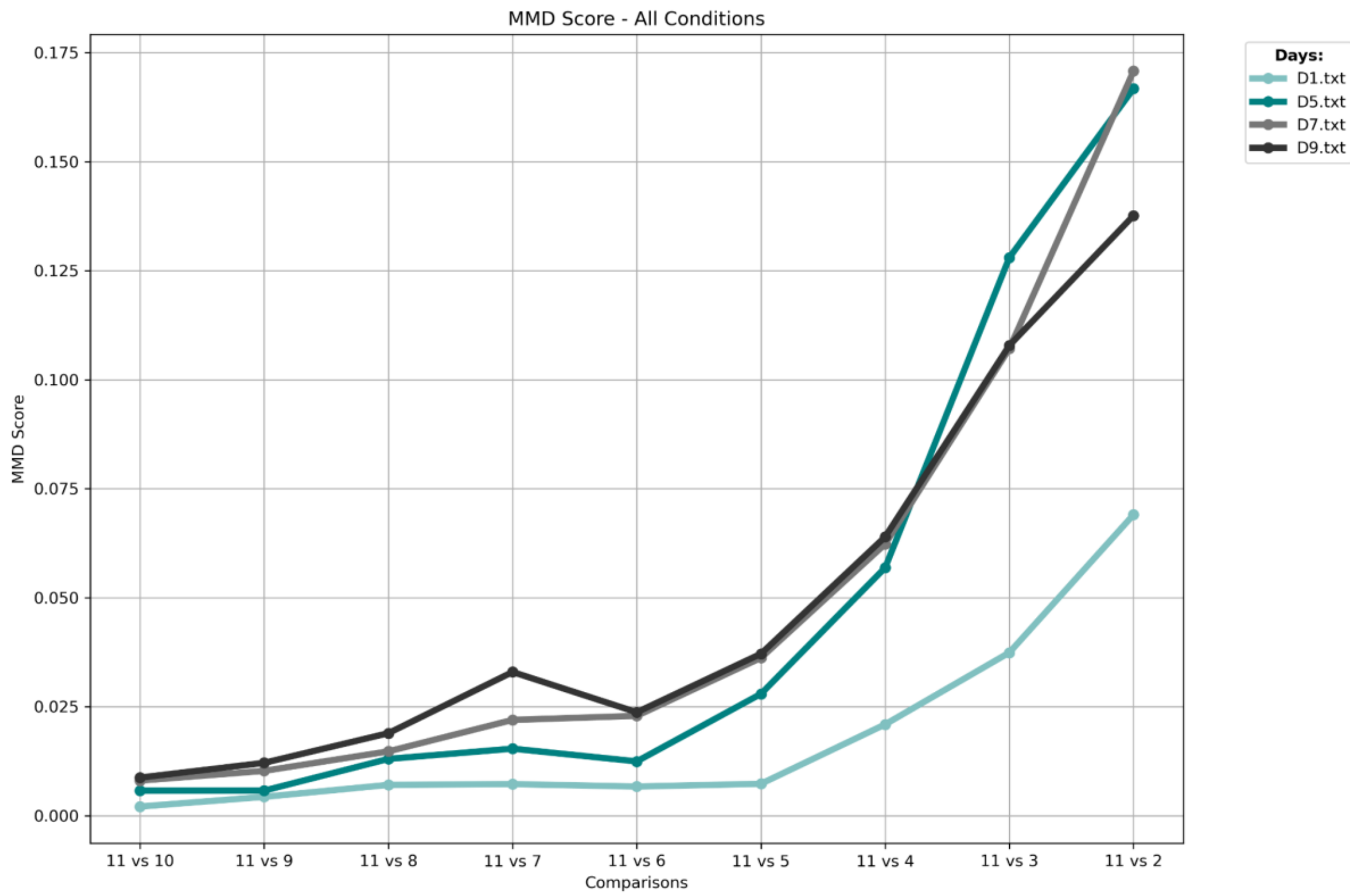

Figure S5.1: MMD plot of data processed with **Z-score** normalization.

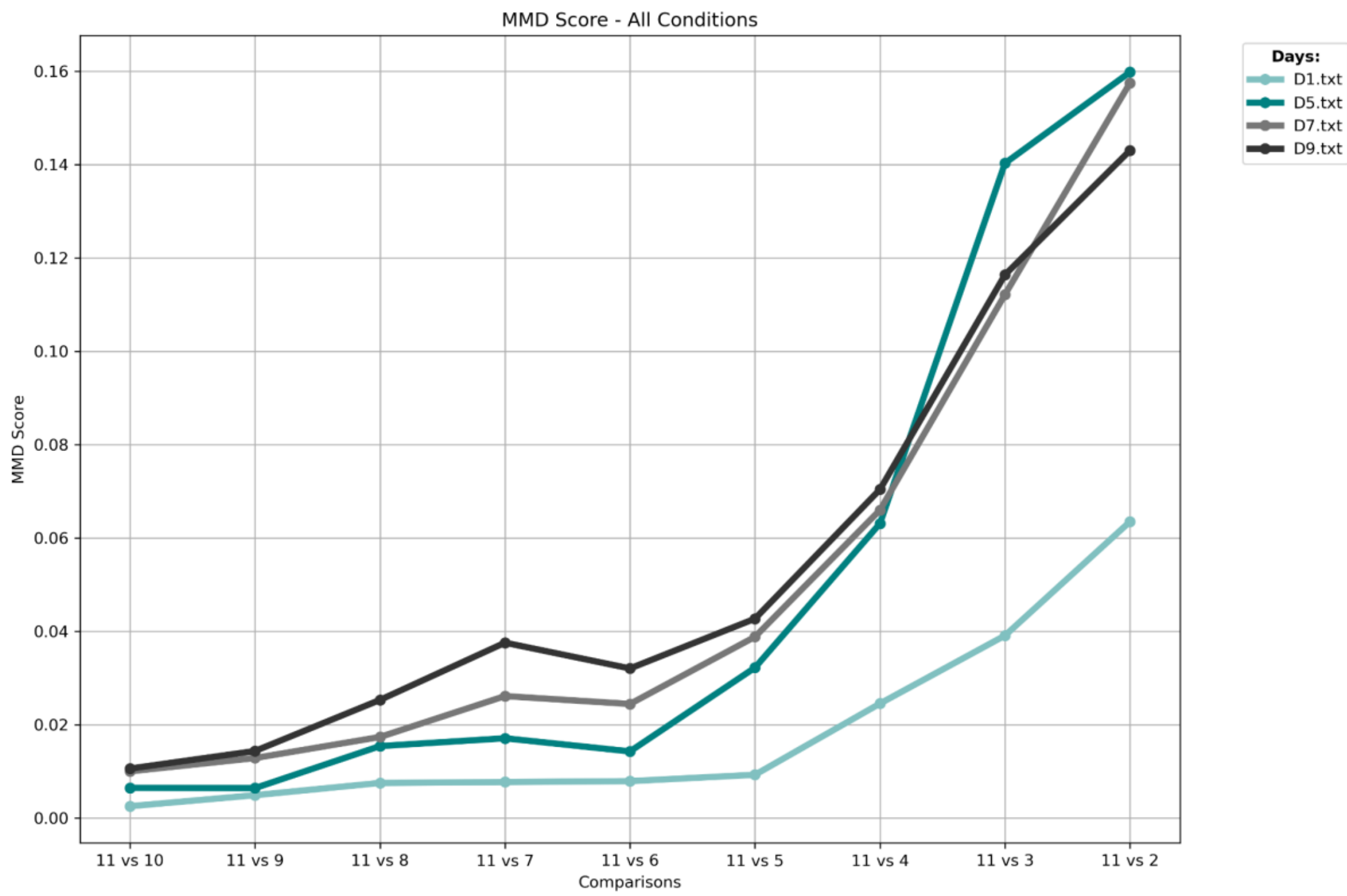

Figure S5.2: MMD plot of data processed with two-step standardization.

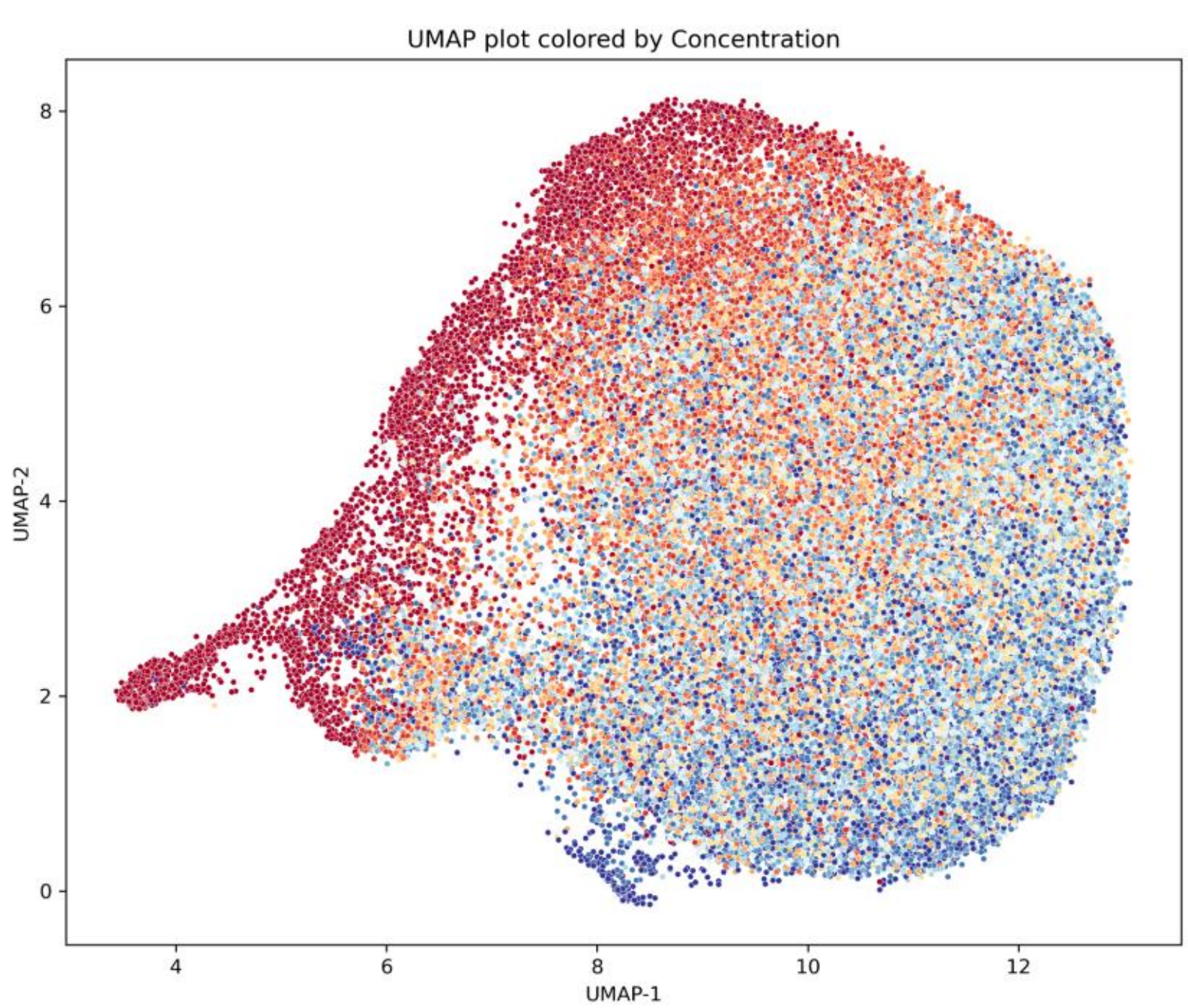

Figure S5.3: UMAP visualization of data processed with **Z-score** normalization.
Concentrations on X-axis: **11 vs 2** - is the highest exposure concentration compared to control, where **11 vs 10** is the lowest exposure compared to control.

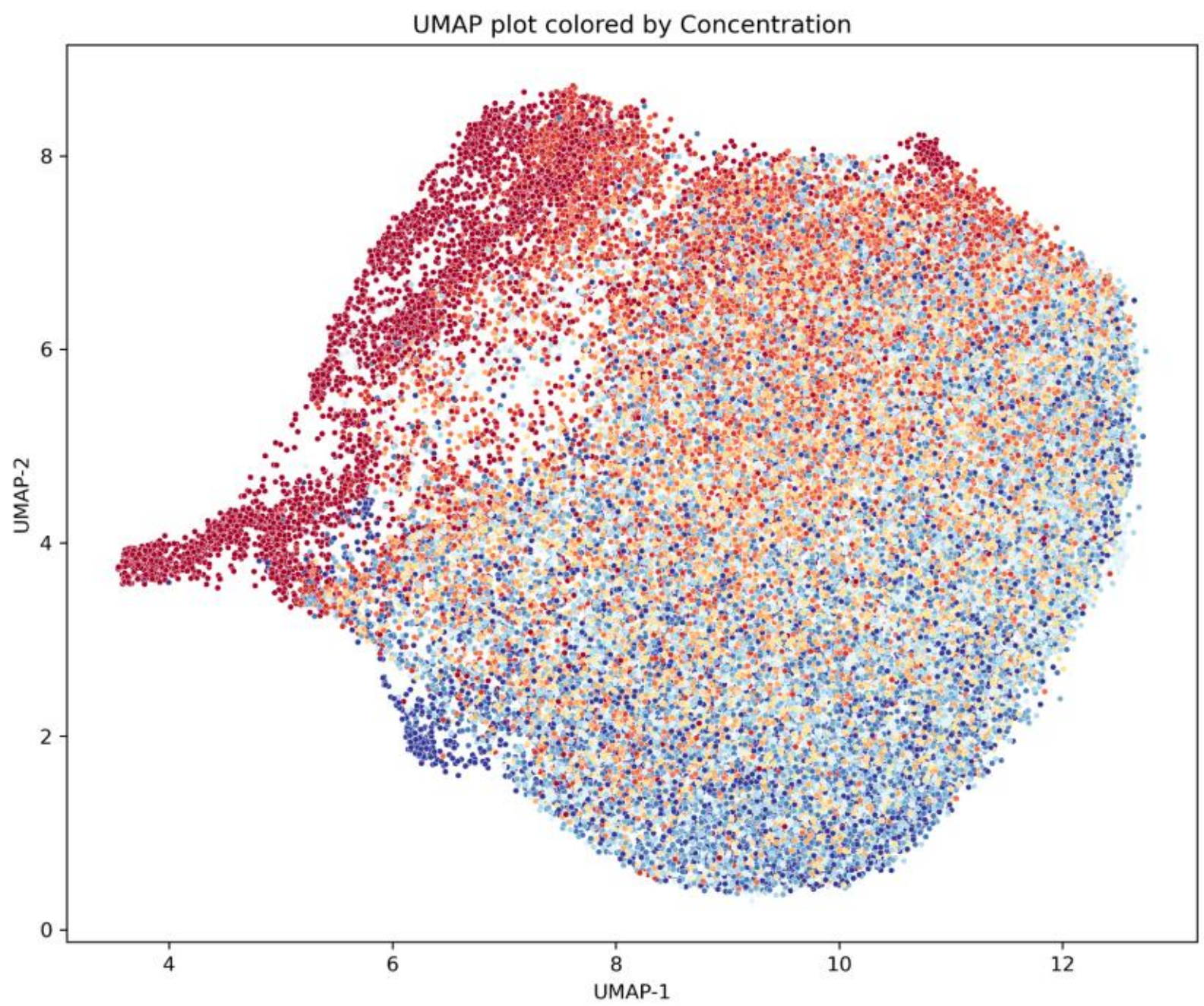

Figure S5.3: UMAP visualization of data processed with two-step standardization.

S6: Concentration-response feature selection results

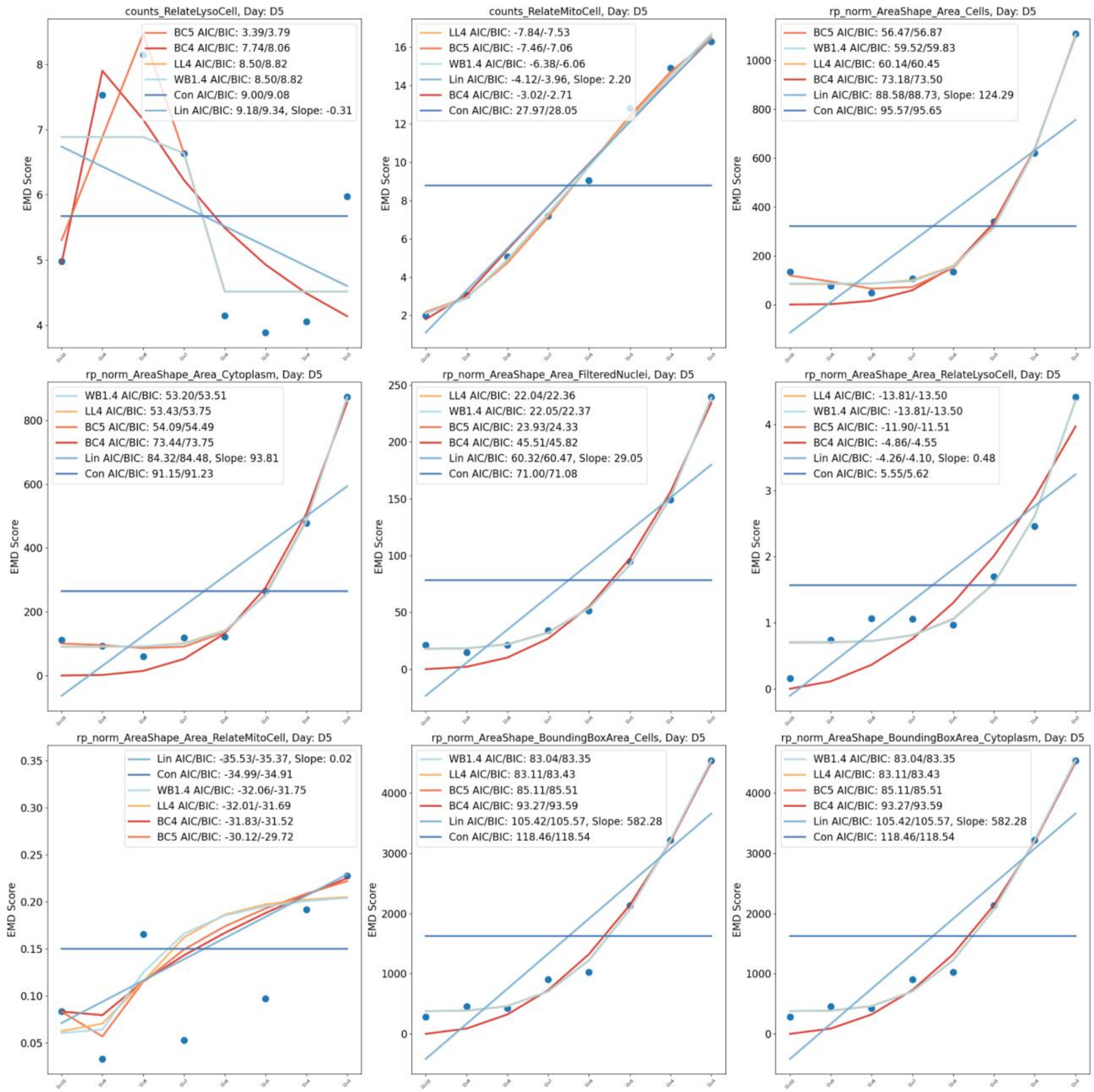

Figure S6: Concentration-response model fitting for each feature, where we calculated EMD values of treatments versus control.

Equations for the drc models:

LL4 - Four-parameter log-logistic:

$$f(x) = c + \frac{d - c}{1 + exp\,[b(ln\,x - ln\,e)]}$$

BC4 - Brain-Cousens four-parameter (c fixed at 0):

$$f(x) = \frac{d + f \cdot x}{1 + exp\,[b(ln\,x - ln\,e)]}$$

BC5 - Brain-Cousens five-parameter (hormetic):

$$f(x) = c + \frac{(d - c) + f \cdot x}{1 + exp\,[b(ln\,x - ln\,e)]}$$

WB1.4 - Four-parameter Weibull (type 1):

$$f(x) = c + (d - c) \cdot exp\,\{- exp\,[b(ln\,x - ln\,e)]\}$$

Lin - Linear:

$$f(x) = m \cdot x + b_0$$

Con - Constant:

$$f(x) = c$$

**Parameter**	**Interpretation**
b	Slope / Hill coefficient at the inflection point
c	Lower asymptote
d	Upper asymptote
e	EC50
f	Hormesis coefficient
m, b_0	Slope and intercept

Multiple variations of additional plots with concentration-response model fitting feature selection are available at https://doi.org/10.5281/zenodo.17951792.

S7: Concentration-response experiments

A second 96-well plate was used in parallel with the plates used for image analysis to assess cell metabolic activity (alamarBlue™, AB), cell membrane integrity (5-Carboxyfluorescein Diacetate, Acetoxymethyl Ester, CFDA-AM), and cell counting (Hoechst-33342). For this purpose, following the removal of the exposure medium, the cells were washed with PBS, supplemented with Ca^{2+} and Mg^{2+} (BioWest, Nuaillé, France). Next, AB and CFDA-AM (5% v/v and 4 µM, respectively, dissolved in PBS, supplemented with Ca^{2+} and Mg^{2+}) were added to half of the exposed cells as well as to two wells that had the highest concentration of tebuthiuron but did not contain cells, to detect potential interactions between tebuthiuron and the dyes. In addition, two cell-free wells that had only contained regular cell culture medium were used for background subtraction. Hoechst-33342 was added to the other half of the cells. Following a 30-minute incubation period, AB and CFDA-AM were measured at the corresponding wavelengths (i.e., excitation wavelengths of 530 nm and 493 nm for AB and CFDA-AM, respectively, and emission wavelengths of 594 nm and 541 nm, respectively). Cells were counted as described in the main text, but by imaging the entire well. All measurements were performed on a BioTek Cytation 5 Cell Imaging Multimode Reader (Agilent, Santa Clara, CA, USA).

Following the subtraction of the background, AB and CFDA-AM fluorescence values were normalized by the values of the unexposed, negative control. Hoechst-33342 values did not require background subtraction and were directly normalized to the control. Two-parameter concentration-response curves were fitted using the R package *drc*[1] and results were visualized using *ggplot2*[2]. Table S7 shows the fitted EC50s and hill slopes and Figure 1B shows the resulting curves.

Table S7: Fitted values from concentration-response experiments (based on measured concentrations).

Day	Dye	EC50 (mg/L)	Hill	SE EC50	SE Hill	EC50 CI (Low - High)	Hill CI (Low - High)	RMSE
1	AB	595.32	0.73	100.51	0.11	395.22 – 795.42	0.50 – 0.95	0.144
5	AB	297.96	1.53	33.11	0.27	232.04 – 363.88	0.98 – 2.08	0.192
7	AB	331.54	2.01	22.29	0.31	287.16 – 375.92	1.39 – 2.62	0.142
9	AB	309.81	1.72	18.37	0.20	273.23 – 346.39	1.33 – 2.11	0.109
1	CFDA-AM	1,259,929	735.59	10.00	10.00	1,259,909 – 1,259,949	715.68 – 755.50	0.139
5	CFDA-AM	514.17	1.61	44.91	0.23	424.76 – 603.57	1.14 – 2.07	0.154
7	CFDA-AM	597.13	2.29	39.83	0.37	517.85 – 676.42	1.56 – 3.03	0.151
9	CFDA-AM	555.39	2.24	23.14	0.20	509.33 – 601.45	1.84 – 2.65	0.094
1	Hoechst-33342	67,177.42	10.25	10.00	10.00	67,156 – 67,198	-10.76 – 31.26	0.061
5	Hoechst-33342	708.62	1.34	49.45	0.15	604.74 – 812.51	1.03 – 1.65	0.045
7	Hoechst-33342	530.47	1.14	34.94	0.11	457.08 – 603.87	0.92 – 1.37	0.043
9	Hoechst-33342	464.79	1.07	32.47	0.10	396.57 – 533.01	0.86 – 1.29	0.047

Medium concentrations were analytically determined, following the purification approach described by Balk *et al.*[3] In brief, for each concentration, 50 µL of each of the three wells designated for Hoechst-33342 measurements was pooled in a 1.5 mL Eppendorf tube. Next, 100 µL of this mixture was transferred to another 1.5 mL Eppendorf tube and 400 µL of MeOH was added and the sample was put at -70°C for at least 20 minutes in order to aid protein precipitation. Following 10 min of sonication, the samples were centrifuged at 4350 rcf for 10 min and 100 µL of the supernatant were transferred to another 1.5 ml Eppendorf tube containing 400 µL MeOH, which was used to repeat the process one additional time.

Lastly, the samples were diluted in an equivolumetric MeOH:H_2O mixture to the range of the calibration series (0.2648019 mg/L - 0.0020688 mg/L) and measured on an HPLC-MS/MS (Agilent Technologies, Santa Clara, CA, USA).

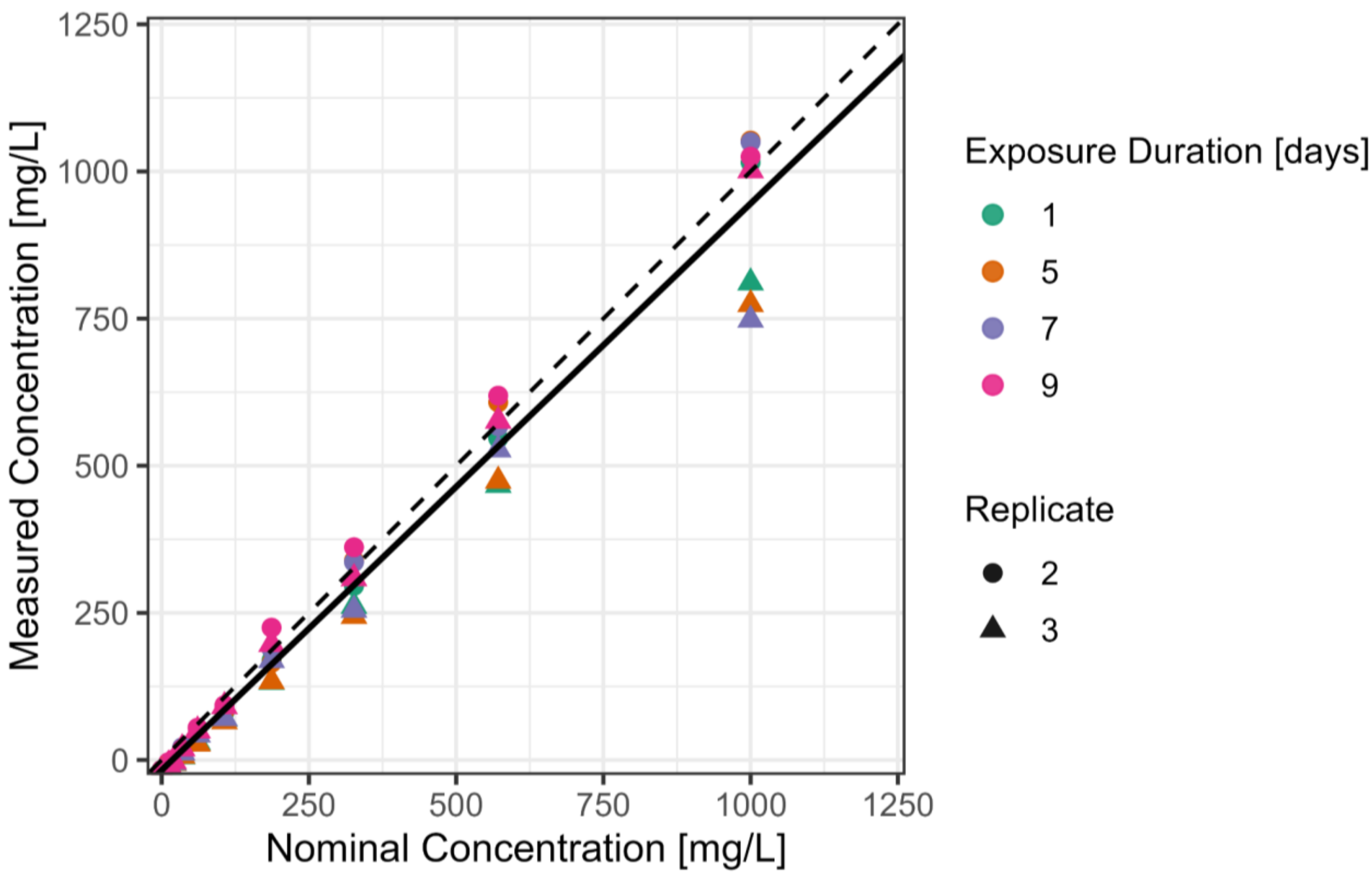

Figure S7.1. Comparison between nominal and measured concentrations. The dashed line corresponds to a perfect equivalence of the two, while the solid line is the result of a linear regression. The result of the linear regression was statistically significant, with concentration strongly predicting measured concentration (slope = 0.963, $p < 2 \times 10^{-16}$), explaining 97.6% of the variance ($R^2 = 0.976$).

Medium concentrations were analytically confirmed to be close to nominal across two independent biological replicates; therefore, concentrations are presented as nominal for all subsequent analyses.

S8: High variability, low activity feature selection

An alternative feature selection approach following Pearson et al. (2022)[4] was applied, using EMD scores to filter features with high variability among biological replicas in control populations (all above upper inner quartile threshold were removed) and features with very low activity between treated and control populations. Specifically, it was a two step removal:

- Removal of “High Variability” features: Uses the 75th percentile (IQR) of control EMD averages as a threshold. Features with control EMD scores above this threshold were removed.

- Removal of “Low Activity” features: Uses a fixed threshold of EMD < 0.05. Features where all treatment conditions in a pair-wise comparison showed EMD scores below 0.05 were removed.

Features that passed both criteria were selected.

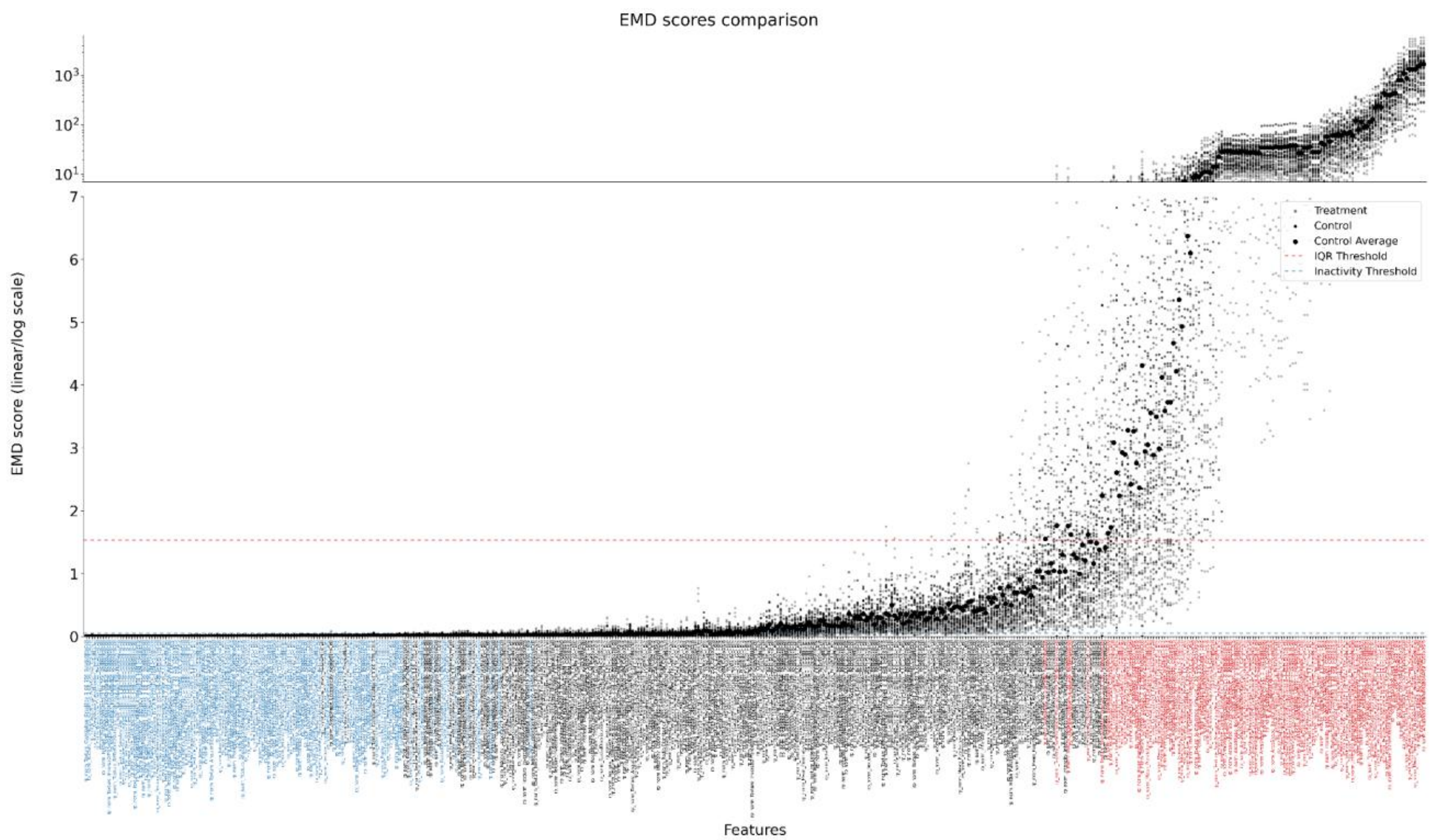

Figure S8.1: EMD scores of all pair-wise comparisons for each feature between biological replicas and between different exposures/controls.

In bold, the average EMD score for each feature. “Low Activity” features are colored blue, “High Variability” features red. Features that were retained are colored black.

Code for selection and visualization available at: https://github.com/NIB-SI/HCS-proc/blob/main/scripts/alternative/high_low_feature_selection/plots_emd_high_low.py

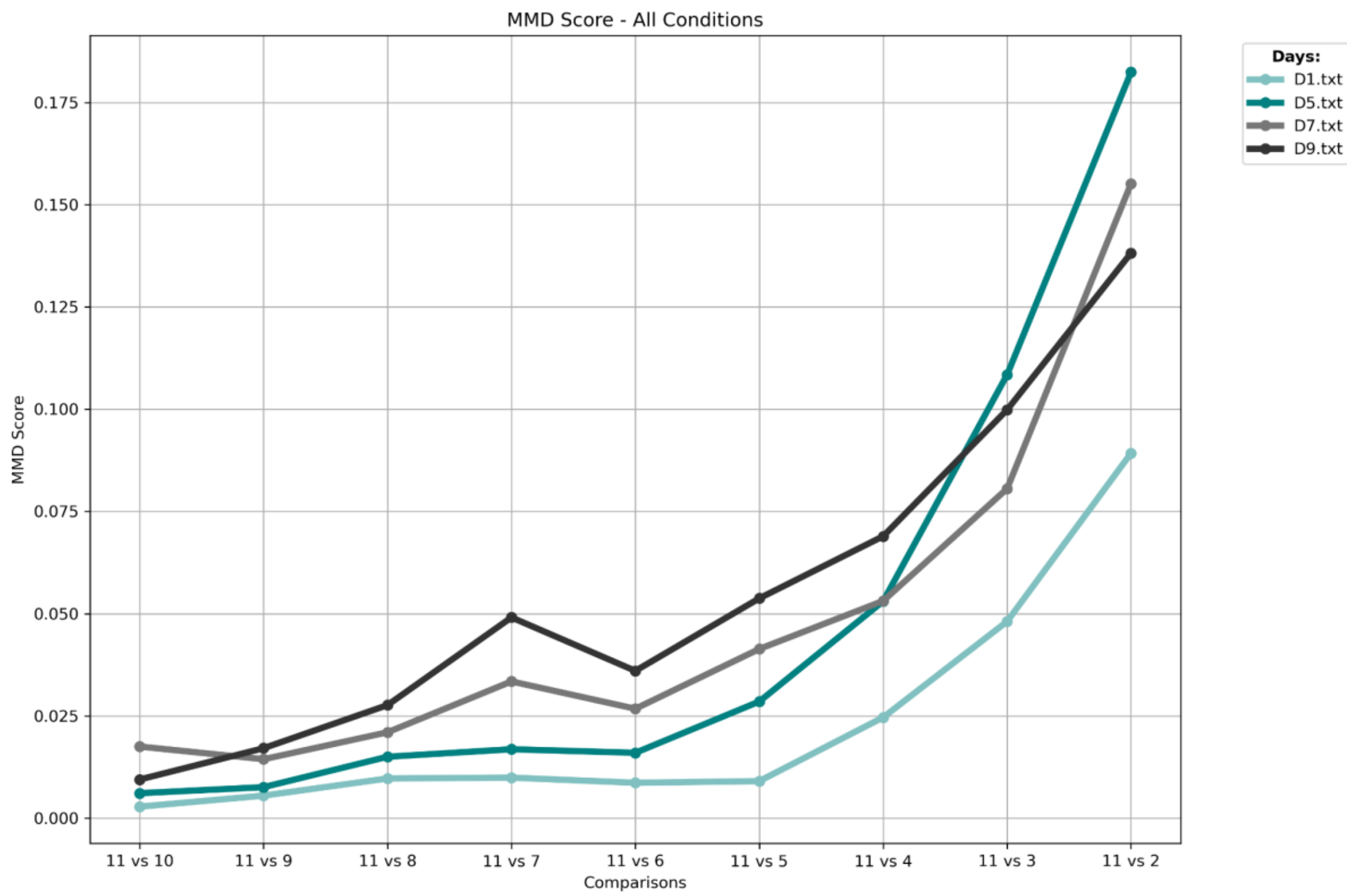

Figure S8.2: MMD plot of data processed with high variability and low activity feature selection.

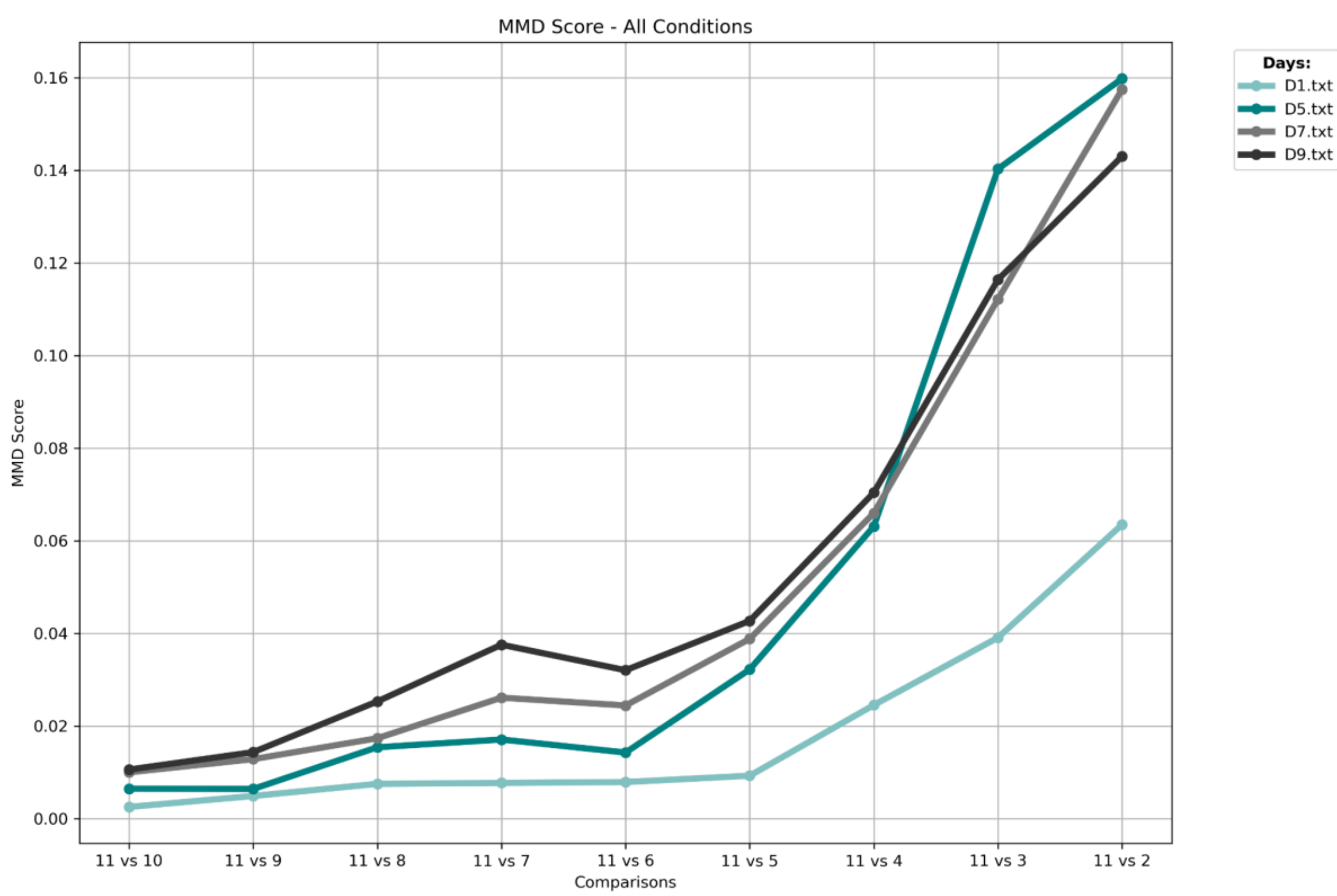

Figure S8.3: MMD plot of data processed with concentration-response model fitting feature selection.

Concentrations on X-axis: **11 vs 2** - is the highest exposure concentration compared to control, where **11 vs 10** is the lowest exposure compared to control.

S9: Effect of multiple highly similar features on global UMAP structure

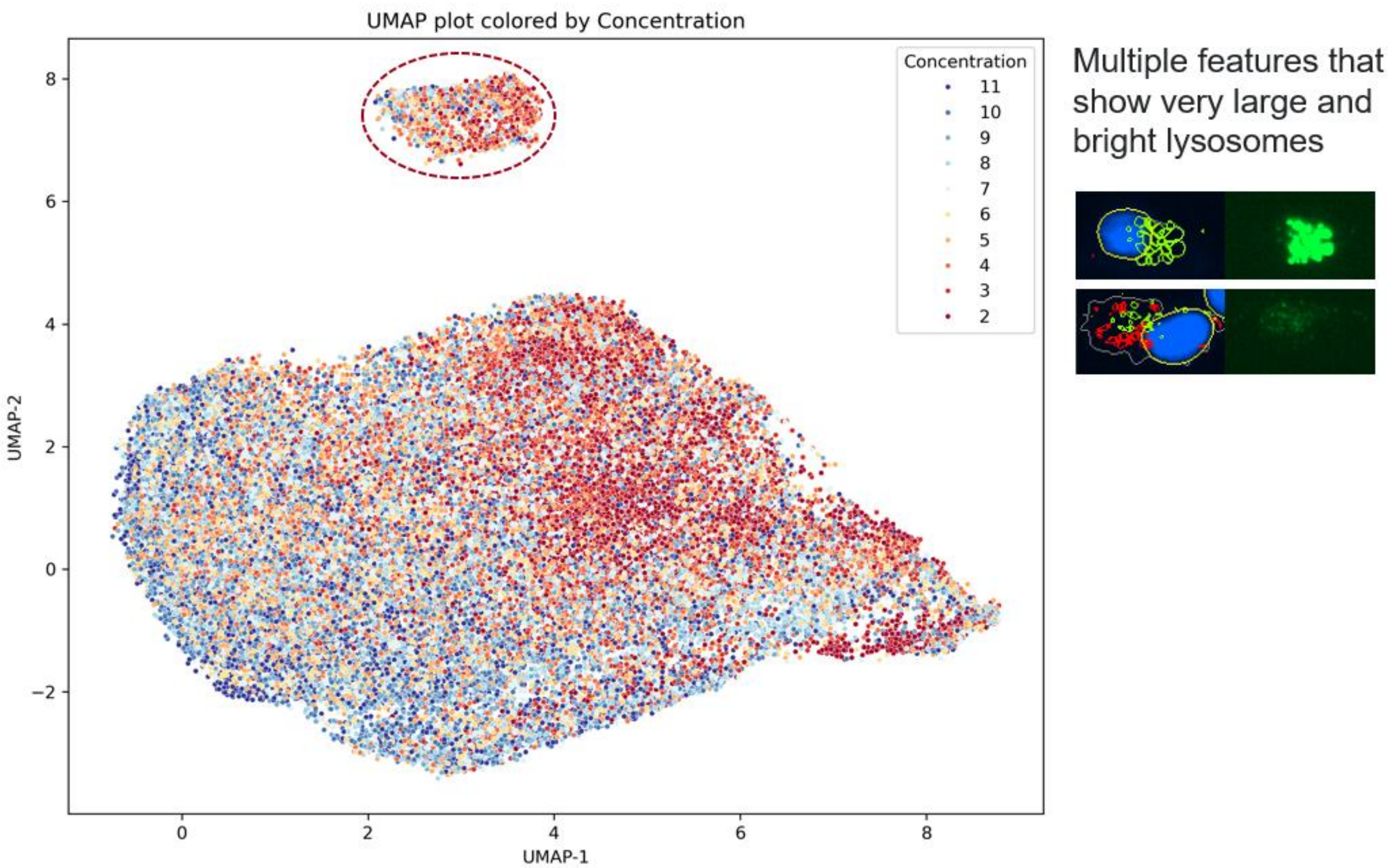

Figure S9: Day 1 UMAP without correlation-based filtering

List of similar features that show high lysosomal signal:
rp_norm_Intensity_StdIntensityEdge_GrayLys_Cytoplasm
rp_norm_Intensity_StdIntensity_GrayLys_Cells
rp_norm_Intensity_StdIntensity_GrayLys_Cytoplasm
rp_norm_Texture_InfoMeas1_GrayLys_3_00_256_Cells
rp_norm_Texture_SumVariance_GrayLys_3_00_256_Cells
rp_norm_Texture_SumVariance_GrayLys_3_00_256_Cytoplasm
rp_norm_Texture_Variance_GrayLys_3_00_256_Cells
rp_norm_Texture_Variance_GrayLys_3_00_256_Cytoplasm

After filtering, we only retained *rp_norm_Texture_InfoMeas1_GrayLys_3_00_256_Cells*.

S10: MMD, EMD and Mahalanobis distance comparison

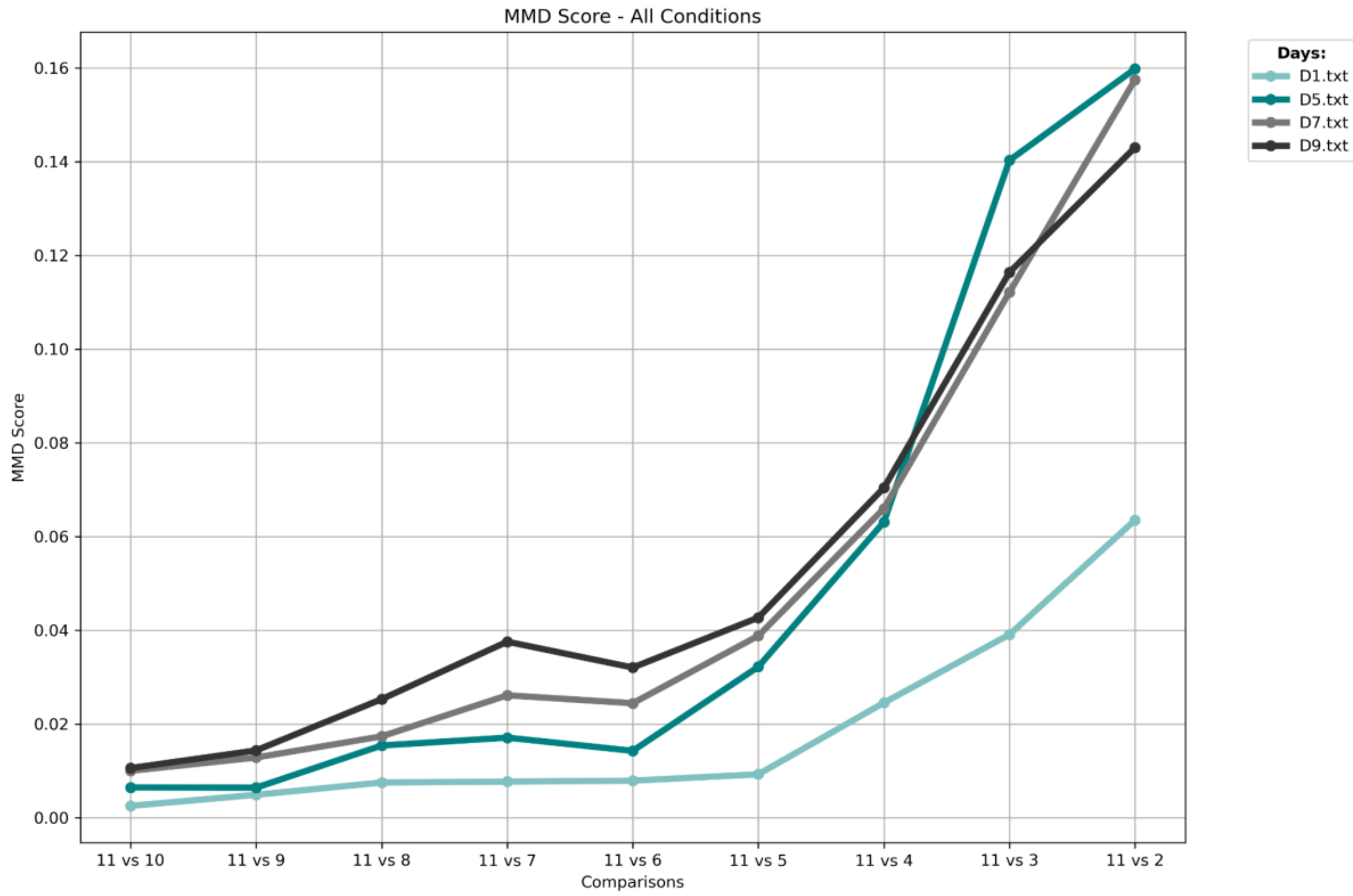

Figure S10.1: MMD plot for comparison of different concentrations against the control for each day.

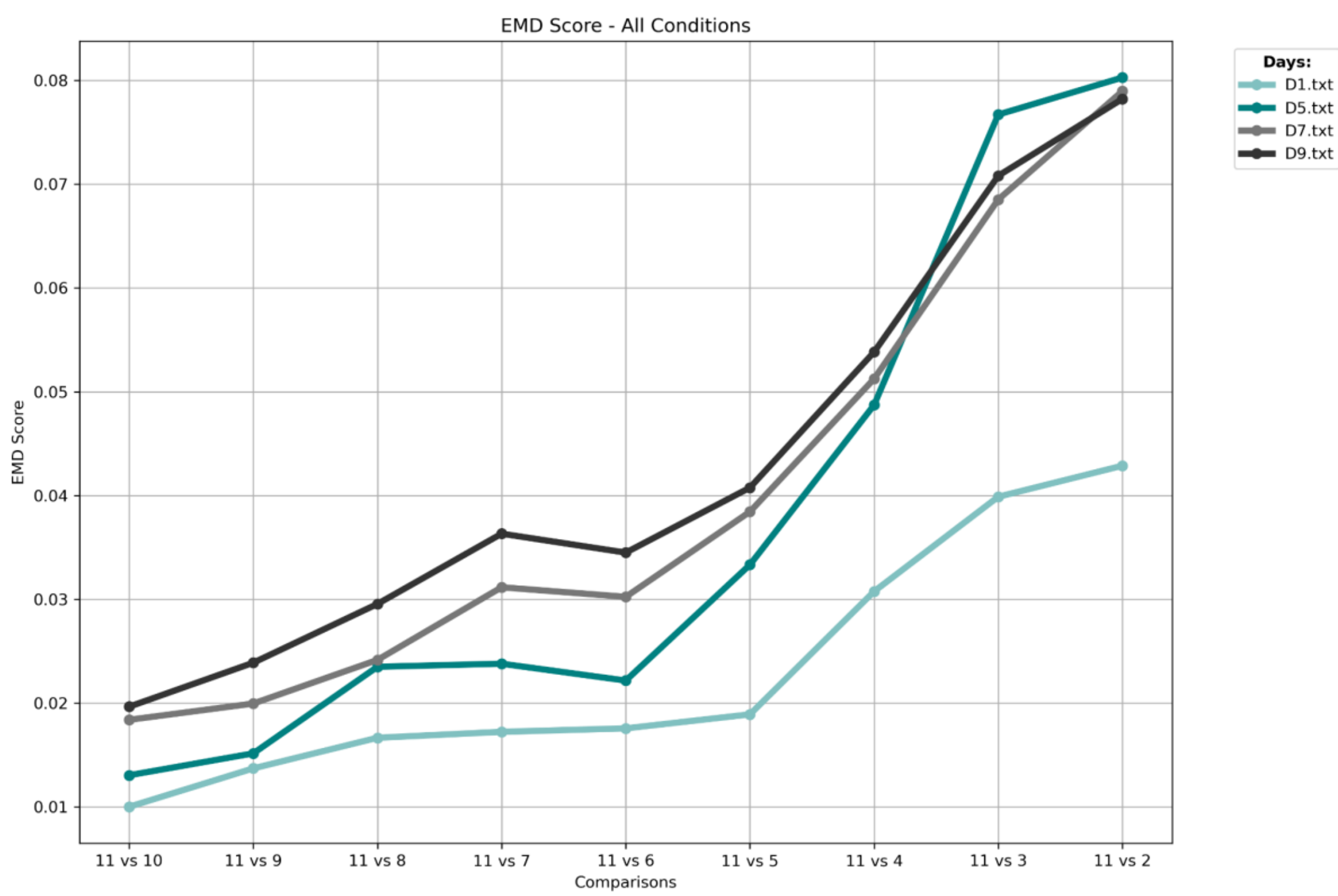

Figure S10.2: EMD plot for comparison of different concentrations against the control for each day.

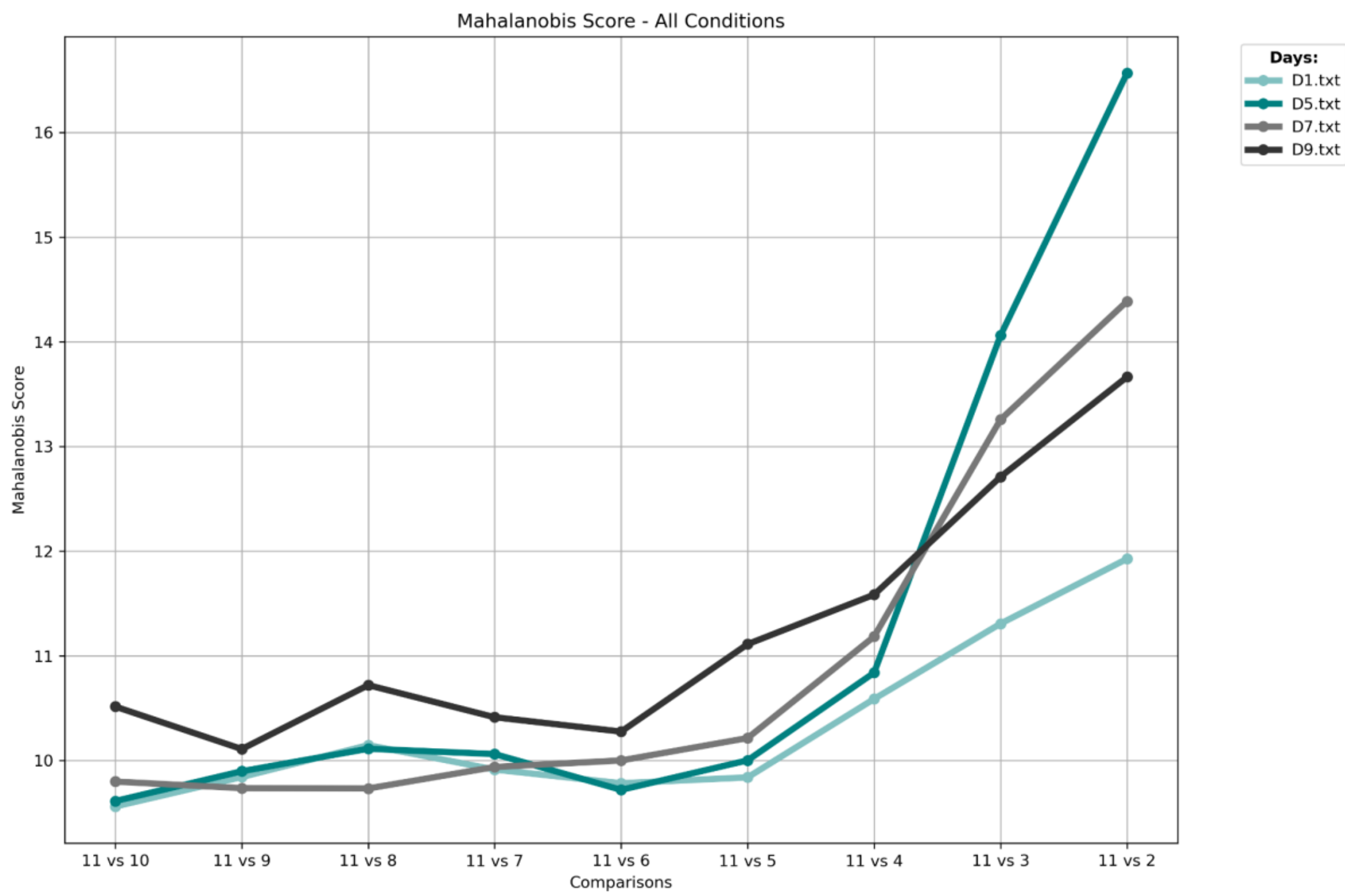

Figure S10.3: Mahalanobis plot for comparison of different concentrations against the control for each day.

Concentrations on X-axis: **11 vs 2** - is the highest exposure concentration compared to control, where **11 vs 10** is the lowest exposure compared to control.

Both MMD and EMD performed similarly, with MMD showing clearer separation at higher exposure concentrations. Both metrics retained temporal and concentration-dependent responses across different processing conditions (Supplementary Files), demonstrating remarkable robustness for routine evaluation of high-dimensional feature profiles in HCS assays. Mahalanobis distance proved less sensitive in separating both temporal and concentration-dependent responses.

S11: List of features

List S11.1: List of extracted features with CellProfiler aggregated per cell before feature selection:

AreaShape_Area_Cells
AreaShape_Area_Cytoplasm
AreaShape_Area_FilteredNuclei
AreaShape_Area_RelateLysoCell
AreaShape_Area_RelateMitoCell
AreaShape_BoundingBoxArea_Cells
AreaShape_BoundingBoxArea_Cytoplasm
AreaShape_BoundingBoxArea_FilteredNuclei

AreaShape_BoundingBoxArea_RelateLysoCell
AreaShape_BoundingBoxArea_RelateMitoCell
AreaShape_BoundingBoxMaximum_X_Cells
AreaShape_BoundingBoxMaximum_X_Cytoplasm
AreaShape_BoundingBoxMaximum_X_FilteredNuclei
AreaShape_BoundingBoxMaximum_X_RelateLysoCell
AreaShape_BoundingBoxMaximum_X_RelateMitoCell
AreaShape_BoundingBoxMaximum_Y_Cells
AreaShape_BoundingBoxMaximum_Y_Cytoplasm
AreaShape_BoundingBoxMaximum_Y_FilteredNuclei
AreaShape_BoundingBoxMaximum_Y_RelateLysoCell
AreaShape_BoundingBoxMaximum_Y_RelateMitoCell
AreaShape_BoundingBoxMinimum_X_Cells
AreaShape_BoundingBoxMinimum_X_Cytoplasm
AreaShape_BoundingBoxMinimum_X_FilteredNuclei
AreaShape_BoundingBoxMinimum_X_RelateLysoCell
AreaShape_BoundingBoxMinimum_X_RelateMitoCell
AreaShape_BoundingBoxMinimum_Y_Cells
AreaShape_BoundingBoxMinimum_Y_Cytoplasm
AreaShape_BoundingBoxMinimum_Y_FilteredNuclei
AreaShape_BoundingBoxMinimum_Y_RelateLysoCell
AreaShape_BoundingBoxMinimum_Y_RelateMitoCell
AreaShape_Center_X_Cells
AreaShape_Center_X_Cytoplasm
AreaShape_Center_X_FilteredNuclei
AreaShape_Center_X_RelateLysoCell
AreaShape_Center_X_RelateMitoCell
AreaShape_Center_Y_Cells
AreaShape_Center_Y_Cytoplasm
AreaShape_Center_Y_FilteredNuclei
AreaShape_Center_Y_RelateLysoCell
AreaShape_Center_Y_RelateMitoCell
AreaShape_Compactness_Cells
AreaShape_Compactness_Cytoplasm
AreaShape_Compactness_FilteredNuclei
AreaShape_Compactness_RelateLysoCell
AreaShape_Compactness_RelateMitoCell
AreaShape_ConvexArea_Cells
AreaShape_ConvexArea_Cytoplasm
AreaShape_ConvexArea_FilteredNuclei
AreaShape_ConvexArea_RelateLysoCell
AreaShape_ConvexArea_RelateMitoCell
AreaShape_Eccentricity_Cells
AreaShape_Eccentricity_Cytoplasm
AreaShape_Eccentricity_FilteredNuclei
AreaShape_Eccentricity_RelateLysoCell
AreaShape_Eccentricity_RelateMitoCell
AreaShape_EquivalentDiameter_Cells
AreaShape_EquivalentDiameter_Cytoplasm
AreaShape_EquivalentDiameter_FilteredNuclei
AreaShape_EquivalentDiameter_RelateLysoCell
AreaShape_EquivalentDiameter_RelateMitoCell
AreaShape_EulerNumber_Cells
AreaShape_EulerNumber_Cytoplasm
AreaShape_EulerNumber_FilteredNuclei
AreaShape_EulerNumber_RelateLysoCell
AreaShape_EulerNumber_RelateMitoCell

AreaShape_Extent_Cells
AreaShape_Extent_Cytoplasm
AreaShape_Extent_FilteredNuclei
AreaShape_Extent_RelateLysoCell
AreaShape_Extent_RelateMitoCell
AreaShape_FormFactor_Cells
AreaShape_FormFactor_Cytoplasm
AreaShape_FormFactor_FilteredNuclei
AreaShape_FormFactor_RelateLysoCell
AreaShape_FormFactor_RelateMitoCell
AreaShape_MajorAxisLength_Cells
AreaShape_MajorAxisLength_Cytoplasm
AreaShape_MajorAxisLength_FilteredNuclei
AreaShape_MajorAxisLength_RelateLysoCell
AreaShape_MajorAxisLength_RelateMitoCell
AreaShape_MaxFeretDiameter_Cells
AreaShape_MaxFeretDiameter_Cytoplasm
AreaShape_MaxFeretDiameter_FilteredNuclei
AreaShape_MaxFeretDiameter_RelateLysoCell
AreaShape_MaxFeretDiameter_RelateMitoCell
AreaShape_MaximumRadius_Cells
AreaShape_MaximumRadius_Cytoplasm
AreaShape_MaximumRadius_FilteredNuclei
AreaShape_MaximumRadius_RelateLysoCell
AreaShape_MaximumRadius_RelateMitoCell
AreaShape_MeanRadius_Cells
AreaShape_MeanRadius_Cytoplasm
AreaShape_MeanRadius_FilteredNuclei
AreaShape_MeanRadius_RelateLysoCell
AreaShape_MeanRadius_RelateMitoCell
AreaShape_MedianRadius_Cells
AreaShape_MedianRadius_Cytoplasm
AreaShape_MedianRadius_FilteredNuclei
AreaShape_MedianRadius_RelateLysoCell
AreaShape_MedianRadius_RelateMitoCell
AreaShape_MinFeretDiameter_Cells
AreaShape_MinFeretDiameter_Cytoplasm
AreaShape_MinFeretDiameter_FilteredNuclei
AreaShape_MinFeretDiameter_RelateLysoCell
AreaShape_MinFeretDiameter_RelateMitoCell
AreaShape_MinorAxisLength_Cells
AreaShape_MinorAxisLength_Cytoplasm
AreaShape_MinorAxisLength_FilteredNuclei
AreaShape_MinorAxisLength_RelateLysoCell
AreaShape_MinorAxisLength_RelateMitoCell
AreaShape_Orientation_Cells
AreaShape_Orientation_Cytoplasm
AreaShape_Orientation_FilteredNuclei
AreaShape_Orientation_RelateLysoCell
AreaShape_Orientation_RelateMitoCell
AreaShape_Perimeter_Cells
AreaShape_Perimeter_Cytoplasm
AreaShape_Perimeter_FilteredNuclei
AreaShape_Perimeter_RelateLysoCell
AreaShape_Perimeter_RelateMitoCell
AreaShape_Solidity_Cells
AreaShape_Solidity_Cytoplasm

AreaShape_Solidity_FilteredNuclei
AreaShape_Solidity_RelateLysoCell
AreaShape_Solidity_RelateMitoCell
Correlation_Correlation_GrayLys_GrayMito_Cells
Correlation_Correlation_GrayLys_GrayNuclei_Cells
Correlation_Correlation_GrayMito_GrayNuclei_Cells
Correlation_Costes_GrayLys_GrayMito_Cells
Correlation_Costes_GrayLys_GrayNuclei_Cells
Correlation_Costes_GrayMito_GrayLys_Cells
Correlation_Costes_GrayMito_GrayNuclei_Cells
Correlation_Costes_GrayNuclei_GrayLys_Cells
Correlation_Costes_GrayNuclei_GrayMito_Cells
Correlation_K_GrayLys_GrayMito_Cells
Correlation_K_GrayLys_GrayNuclei_Cells
Correlation_K_GrayMito_GrayLys_Cells
Correlation_K_GrayMito_GrayNuclei_Cells
Correlation_K_GrayNuclei_GrayLys_Cells
Correlation_K_GrayNuclei_GrayMito_Cells
Correlation_Manders_GrayLys_GrayMito_Cells
Correlation_Manders_GrayLys_GrayNuclei_Cells
Correlation_Manders_GrayMito_GrayLys_Cells
Correlation_Manders_GrayMito_GrayNuclei_Cells
Correlation_Manders_GrayNuclei_GrayLys_Cells
Correlation_Manders_GrayNuclei_GrayMito_Cells
Correlation_Overlap_GrayLys_GrayMito_Cells
Correlation_Overlap_GrayLys_GrayNuclei_Cells
Correlation_Overlap_GrayMito_GrayNuclei_Cells
Correlation_RWC_GrayLys_GrayMito_Cells
Correlation_RWC_GrayLys_GrayNuclei_Cells
Correlation_RWC_GrayMito_GrayLys_Cells
Correlation_RWC_GrayMito_GrayNuclei_Cells
Correlation_RWC_GrayNuclei_GrayLys_Cells
Correlation_RWC_GrayNuclei_GrayMito_Cells
Granularity_10_GrayLys_RelateLysoCell
Granularity_10_GrayMito_RelateMitoCell
Granularity_10_GrayNuclei_FilteredNuclei
Granularity_11_GrayLys_RelateLysoCell
Granularity_11_GrayMito_RelateMitoCell
Granularity_11_GrayNuclei_FilteredNuclei
Granularity_12_GrayLys_RelateLysoCell
Granularity_12_GrayMito_RelateMitoCell
Granularity_12_GrayNuclei_FilteredNuclei
Granularity_13_GrayLys_RelateLysoCell
Granularity_13_GrayMito_RelateMitoCell
Granularity_13_GrayNuclei_FilteredNuclei
Granularity_14_GrayLys_RelateLysoCell
Granularity_14_GrayMito_RelateMitoCell
Granularity_14_GrayNuclei_FilteredNuclei
Granularity_15_GrayLys_RelateLysoCell
Granularity_15_GrayMito_RelateMitoCell
Granularity_16_GrayMito_RelateMitoCell
Granularity_17_GrayMito_RelateMitoCell
Granularity_18_GrayMito_RelateMitoCell
Granularity_19_GrayMito_RelateMitoCell
Granularity_1_GrayLys_RelateLysoCell
Granularity_1_GrayMito_RelateMitoCell
Granularity_1_GrayNuclei_FilteredNuclei

Granularity_20_GrayMito_RelateMitoCell
Granularity_2_GrayLys_RelateLysoCell
Granularity_2_GrayMito_RelateMitoCell
Granularity_2_GrayNuclei_FilteredNuclei
Granularity_3_GrayLys_RelateLysoCell
Granularity_3_GrayMito_RelateMitoCell
Granularity_3_GrayNuclei_FilteredNuclei
Granularity_4_GrayLys_RelateLysoCell
Granularity_4_GrayMito_RelateMitoCell
Granularity_4_GrayNuclei_FilteredNuclei
Granularity_5_GrayLys_RelateLysoCell
Granularity_5_GrayMito_RelateMitoCell
Granularity_5_GrayNuclei_FilteredNuclei
Granularity_6_GrayLys_RelateLysoCell
Granularity_6_GrayMito_RelateMitoCell
Granularity_6_GrayNuclei_FilteredNuclei
Granularity_7_GrayLys_RelateLysoCell
Granularity_7_GrayMito_RelateMitoCell
Granularity_7_GrayNuclei_FilteredNuclei
Granularity_8_GrayLys_RelateLysoCell
Granularity_8_GrayMito_RelateMitoCell
Granularity_8_GrayNuclei_FilteredNuclei
Granularity_9_GrayLys_RelateLysoCell
Granularity_9_GrayMito_RelateMitoCell
Granularity_9_GrayNuclei_FilteredNuclei
Intensity_IntegratedIntensityEdge_GrayLys_Cells
Intensity_IntegratedIntensityEdge_GrayLys_Cytoplasm
Intensity_IntegratedIntensityEdge_GrayLys_RelateLysoCell
Intensity_IntegratedIntensityEdge_GrayMito_Cells
Intensity_IntegratedIntensityEdge_GrayMito_Cytoplasm
Intensity_IntegratedIntensityEdge_GrayMito_RelateMitoCell
Intensity_IntegratedIntensityEdge_GrayNuclei_Cells
Intensity_IntegratedIntensityEdge_GrayNuclei_FilteredNuclei
Intensity_IntegratedIntensity_GrayLys_Cells
Intensity_IntegratedIntensity_GrayLys_Cytoplasm
Intensity_IntegratedIntensity_GrayLys_RelateLysoCell
Intensity_IntegratedIntensity_GrayMito_Cells
Intensity_IntegratedIntensity_GrayMito_Cytoplasm
Intensity_IntegratedIntensity_GrayMito_RelateMitoCell
Intensity_IntegratedIntensity_GrayNuclei_Cells
Intensity_IntegratedIntensity_GrayNuclei_FilteredNuclei
Intensity_LowerQuartileIntensity_GrayLys_Cells
Intensity_LowerQuartileIntensity_GrayLys_Cytoplasm
Intensity_LowerQuartileIntensity_GrayLys_RelateLysoCell
Intensity_LowerQuartileIntensity_GrayMito_Cells
Intensity_LowerQuartileIntensity_GrayMito_Cytoplasm
Intensity_LowerQuartileIntensity_GrayMito_RelateMitoCell
Intensity_LowerQuartileIntensity_GrayNuclei_Cells
Intensity_LowerQuartileIntensity_GrayNuclei_FilteredNuclei
Intensity_MADIntensity_GrayLys_Cells
Intensity_MADIntensity_GrayLys_Cytoplasm
Intensity_MADIntensity_GrayLys_RelateLysoCell
Intensity_MADIntensity_GrayMito_Cells
Intensity_MADIntensity_GrayMito_Cytoplasm
Intensity_MADIntensity_GrayMito_RelateMitoCell
Intensity_MADIntensity_GrayNuclei_Cells
Intensity_MADIntensity_GrayNuclei_FilteredNuclei

Intensity_MassDisplacement_GrayLys_Cells
Intensity_MassDisplacement_GrayLys_Cytoplasm
Intensity_MassDisplacement_GrayLys_RelateLysoCell
Intensity_MassDisplacement_GrayMito_Cells
Intensity_MassDisplacement_GrayMito_Cytoplasm
Intensity_MassDisplacement_GrayMito_RelateMitoCell
Intensity_MassDisplacement_GrayNuclei_Cells
Intensity_MassDisplacement_GrayNuclei_FilteredNuclei
Intensity_MaxIntensityEdge_GrayLys_Cells
Intensity_MaxIntensityEdge_GrayLys_Cytoplasm
Intensity_MaxIntensityEdge_GrayLys_RelateLysoCell
Intensity_MaxIntensityEdge_GrayMito_Cells
Intensity_MaxIntensityEdge_GrayMito_Cytoplasm
Intensity_MaxIntensityEdge_GrayMito_RelateMitoCell
Intensity_MaxIntensityEdge_GrayNuclei_Cells
Intensity_MaxIntensityEdge_GrayNuclei_FilteredNuclei
Intensity_MaxIntensity_GrayLys_Cells
Intensity_MaxIntensity_GrayLys_Cytoplasm
Intensity_MaxIntensity_GrayLys_RelateLysoCell
Intensity_MaxIntensity_GrayMito_Cells
Intensity_MaxIntensity_GrayMito_Cytoplasm
Intensity_MaxIntensity_GrayMito_RelateMitoCell
Intensity_MaxIntensity_GrayNuclei_Cells
Intensity_MaxIntensity_GrayNuclei_FilteredNuclei
Intensity_MeanIntensityEdge_GrayLys_Cells
Intensity_MeanIntensityEdge_GrayLys_Cytoplasm
Intensity_MeanIntensityEdge_GrayLys_RelateLysoCell
Intensity_MeanIntensityEdge_GrayMito_Cells
Intensity_MeanIntensityEdge_GrayMito_Cytoplasm
Intensity_MeanIntensityEdge_GrayMito_RelateMitoCell
Intensity_MeanIntensityEdge_GrayNuclei_Cells
Intensity_MeanIntensityEdge_GrayNuclei_FilteredNuclei
Intensity_MeanIntensity_GrayLys_Cells
Intensity_MeanIntensity_GrayLys_Cytoplasm
Intensity_MeanIntensity_GrayLys_RelateLysoCell
Intensity_MeanIntensity_GrayMito_Cells
Intensity_MeanIntensity_GrayMito_Cytoplasm
Intensity_MeanIntensity_GrayMito_RelateMitoCell
Intensity_MeanIntensity_GrayNuclei_Cells
Intensity_MeanIntensity_GrayNuclei_FilteredNuclei
Intensity_MedianIntensity_GrayLys_Cells
Intensity_MedianIntensity_GrayLys_Cytoplasm
Intensity_MedianIntensity_GrayLys_RelateLysoCell
Intensity_MedianIntensity_GrayMito_Cells
Intensity_MedianIntensity_GrayMito_Cytoplasm
Intensity_MedianIntensity_GrayMito_RelateMitoCell
Intensity_MedianIntensity_GrayNuclei_Cells
Intensity_MedianIntensity_GrayNuclei_FilteredNuclei
Intensity_MinIntensityEdge_GrayLys_Cells
Intensity_MinIntensityEdge_GrayLys_Cytoplasm
Intensity_MinIntensityEdge_GrayLys_RelateLysoCell
Intensity_MinIntensityEdge_GrayMito_Cells
Intensity_MinIntensityEdge_GrayMito_Cytoplasm
Intensity_MinIntensityEdge_GrayMito_RelateMitoCell
Intensity_MinIntensityEdge_GrayNuclei_Cells
Intensity_MinIntensityEdge_GrayNuclei_FilteredNuclei
Intensity_MinIntensity_GrayLys_Cells

Intensity_MinIntensity_GrayLys_Cytoplasm
Intensity_MinIntensity_GrayLys_RelateLysoCell
Intensity_MinIntensity_GrayMito_Cells
Intensity_MinIntensity_GrayMito_Cytoplasm
Intensity_MinIntensity_GrayMito_RelateMitoCell
Intensity_MinIntensity_GrayNuclei_Cells
Intensity_MinIntensity_GrayNuclei_FilteredNuclei
Intensity_StdIntensityEdge_GrayLys_Cells
Intensity_StdIntensityEdge_GrayLys_Cytoplasm
Intensity_StdIntensityEdge_GrayLys_RelateLysoCell
Intensity_StdIntensityEdge_GrayMito_Cells
Intensity_StdIntensityEdge_GrayMito_Cytoplasm
Intensity_StdIntensityEdge_GrayMito_RelateMitoCell
Intensity_StdIntensityEdge_GrayNuclei_Cells
Intensity_StdIntensityEdge_GrayNuclei_FilteredNuclei
Intensity_StdIntensity_GrayLys_Cells
Intensity_StdIntensity_GrayLys_Cytoplasm
Intensity_StdIntensity_GrayLys_RelateLysoCell
Intensity_StdIntensity_GrayMito_Cells
Intensity_StdIntensity_GrayMito_Cytoplasm
Intensity_StdIntensity_GrayMito_RelateMitoCell
Intensity_StdIntensity_GrayNuclei_Cells
Intensity_StdIntensity_GrayNuclei_FilteredNuclei
Intensity_UpperQuartileIntensity_GrayLys_Cells
Intensity_UpperQuartileIntensity_GrayLys_Cytoplasm
Intensity_UpperQuartileIntensity_GrayLys_RelateLysoCell
Intensity_UpperQuartileIntensity_GrayMito_Cells
Intensity_UpperQuartileIntensity_GrayMito_Cytoplasm
Intensity_UpperQuartileIntensity_GrayMito_RelateMitoCell
Intensity_UpperQuartileIntensity_GrayNuclei_Cells
Intensity_UpperQuartileIntensity_GrayNuclei_FilteredNuclei
Mean_PunctaLyso_Distance_Centroid_Cells_Cells
Mean_PunctaLyso_Distance_Centroid_Cytoplasm_Cells
Mean_PunctaLyso_Distance_Centroid_Cytoplasm_Cytoplasm
Mean_PunctaLyso_Distance_Centroid_Cytoplasm_FilteredNuclei
Mean_PunctaLyso_Distance_Centroid_FilteredNuclei_Cells
Mean_PunctaLyso_Distance_Centroid_FilteredNuclei_FilteredNuclei
Mean_PunctaLyso_Distance_Minimum_Cytoplasm_Cells
Mean_PunctaLyso_Distance_Minimum_Cytoplasm_Cytoplasm
Mean_PunctaLyso_Distance_Minimum_Cytoplasm_FilteredNuclei
Mean_PunctaLyso_Number_Object_Number_Cells
Mean_PunctaLyso_Number_Object_Number_Cytoplasm
Mean_PunctaLyso_Number_Object_Number_FilteredNuclei
Number_Object_Number_Cells
Number_Object_Number_Cytoplasm
Number_Object_Number_FilteredNuclei
Number_Object_Number_RelateLysoCell
Number_Object_Number_RelateMitoCell
RadialDistribution_FracAtD_GrayLys_1of4_Cytoplasm
RadialDistribution_FracAtD_GrayLys_2of4_Cytoplasm
RadialDistribution_FracAtD_GrayLys_3of4_Cytoplasm
RadialDistribution_FracAtD_GrayLys_4of4_Cytoplasm
RadialDistribution_FracAtD_GrayMito_1of4_Cytoplasm
RadialDistribution_FracAtD_GrayMito_2of4_Cytoplasm
RadialDistribution_FracAtD_GrayMito_3of4_Cytoplasm
RadialDistribution_FracAtD_GrayMito_4of4_Cytoplasm
RadialDistribution_MeanFrac_GrayLys_1of4_Cytoplasm

RadialDistribution_MeanFrac_GrayLys_2of4_Cytoplasm
RadialDistribution_MeanFrac_GrayLys_3of4_Cytoplasm
RadialDistribution_MeanFrac_GrayLys_4of4_Cytoplasm
RadialDistribution_MeanFrac_GrayMito_1of4_Cytoplasm
RadialDistribution_MeanFrac_GrayMito_2of4_Cytoplasm
RadialDistribution_MeanFrac_GrayMito_3of4_Cytoplasm
RadialDistribution_MeanFrac_GrayMito_4of4_Cytoplasm
RadialDistribution_RadialCV_GrayLys_1of4_Cytoplasm
RadialDistribution_RadialCV_GrayLys_2of4_Cytoplasm
RadialDistribution_RadialCV_GrayLys_3of4_Cytoplasm
RadialDistribution_RadialCV_GrayLys_4of4_Cytoplasm
RadialDistribution_RadialCV_GrayMito_1of4_Cytoplasm
RadialDistribution_RadialCV_GrayMito_2of4_Cytoplasm
RadialDistribution_RadialCV_GrayMito_3of4_Cytoplasm
RadialDistribution_RadialCV_GrayMito_4of4_Cytoplasm
Texture_AngularSecondMoment_GrayLys_3_00/01/02/03_256_Cells
Texture_AngularSecondMoment_GrayLys_3_00/01/02/03_256_Cytoplasm
Texture_AngularSecondMoment_GrayLys_3_00/01/02/03_256_RelateLysoCell
Texture_AngularSecondMoment_GrayMito_3_00/01/02/03_256_Cells
Texture_AngularSecondMoment_GrayMito_3_00/01/02/03_256_Cytoplasm
Texture_AngularSecondMoment_GrayMito_3_00/01/02/03_256_RelateMitoCell
Texture_AngularSecondMoment_GrayNuclei_3_00/01/02/03_256_Cells
Texture_AngularSecondMoment_GrayNuclei_3_00/01/02/03_256_FilteredNuclei
Texture_Contrast_GrayLys_3_00/01/02/03_256_Cells
Texture_Contrast_GrayLys_3_00/01/02/03_256_Cytoplasm
Texture_Contrast_GrayLys_3_00/01/02/03_256_RelateLysoCell
Texture_Contrast_GrayMito_3_00/01/02/03_256_Cells
Texture_Contrast_GrayMito_3_00/01/02/03_256_Cytoplasm
Texture_Contrast_GrayMito_3_00/01/02/03_256_RelateMitoCell
Texture_Contrast_GrayNuclei_3_00/01/02/03_256_Cells
Texture_Contrast_GrayNuclei_3_00/01/02/03_256_FilteredNuclei
Texture_Correlation_GrayLys_3_00/01/02/03_256_Cells
Texture_Correlation_GrayLys_3_00/01/02/03_256_Cytoplasm
Texture_Correlation_GrayLys_3_00/01/02/03_256_RelateLysoCell
Texture_Correlation_GrayMito_3_00/01/02/03_256_Cells
Texture_Correlation_GrayMito_3_00/01/02/03_256_Cytoplasm
Texture_Correlation_GrayMito_3_00/01/02/03_256_RelateMitoCell
Texture_Correlation_GrayNuclei_3_00/01/02/03_256_Cells
Texture_Correlation_GrayNuclei_3_00/01/02/03_256_FilteredNuclei
Texture_DifferenceEntropy_GrayLys_3_00/01/02/03_256_Cells
Texture_DifferenceEntropy_GrayLys_3_00/01/02/03_256_Cytoplasm
Texture_DifferenceEntropy_GrayLys_3_00/01/02/03_256_RelateLysoCell
Texture_DifferenceEntropy_GrayMito_3_00/01/02/03_256_Cells
Texture_DifferenceEntropy_GrayMito_3_00/01/02/03_256_Cytoplasm
Texture_DifferenceEntropy_GrayMito_3_00/01/02/03_256_RelateMitoCell
Texture_DifferenceEntropy_GrayNuclei_3_00/01/02/03_256_Cells
Texture_DifferenceEntropy_GrayNuclei_3_00/01/02/03_256_FilteredNuclei
Texture_DifferenceVariance_GrayLys_3_00/01/02/03_256_Cells
Texture_DifferenceVariance_GrayLys_3_00/01/02/03_256_Cytoplasm
Texture_DifferenceVariance_GrayLys_3_00/01/02/03_256_RelateLysoCell
Texture_DifferenceVariance_GrayMito_3_00/01/02/03_256_Cells
Texture_DifferenceVariance_GrayMito_3_00/01/02/03_256_Cytoplasm
Texture_DifferenceVariance_GrayMito_3_00/01/02/03_256_RelateMitoCell
Texture_DifferenceVariance_GrayNuclei_3_00/01/02/03_256_Cells
Texture_DifferenceVariance_GrayNuclei_3_00/01/02/03_256_FilteredNuclei
Texture_Entropy_GrayLys_3_00/01/02/03_256_Cells
Texture_Entropy_GrayLys_3_00/01/02/03_256_Cytoplasm

Texture_Entropy_GrayLys_3_00/01/02/03_256_RelateLysoCell
Texture_Entropy_GrayMito_3_00/01/02/03_256_Cells
Texture_Entropy_GrayMito_3_00/01/02/03_256_Cytoplasm
Texture_Entropy_GrayMito_3_00/01/02/03_256_RelateMitoCell
Texture_Entropy_GrayNuclei_3_00/01/02/03_256_Cells
Texture_Entropy_GrayNuclei_3_00/01/02/03_256_FilteredNuclei
Texture_InfoMeas1_GrayLys_3_00/01/02/03_256_Cells
Texture_InfoMeas1_GrayLys_3_00/01/02/03_256_Cytoplasm
Texture_InfoMeas1_GrayLys_3_00/01/02/03_256_RelateLysoCell
Texture_InfoMeas1_GrayMito_3_00/01/02/03_256_Cells
Texture_InfoMeas1_GrayMito_3_00/01/02/03_256_Cytoplasm
Texture_InfoMeas1_GrayMito_3_00/01/02/03_256_RelateMitoCell
Texture_InfoMeas1_GrayNuclei_3_00/01/02/03_256_Cells
Texture_InfoMeas1_GrayNuclei_3_00/01/02/03_256_FilteredNuclei
Texture_InfoMeas2_GrayLys_3_00/01/02/03_256_Cells
Texture_InfoMeas2_GrayLys_3_00/01/02/03_256_Cytoplasm
Texture_InfoMeas2_GrayLys_3_00/01/02/03_256_RelateLysoCell
Texture_InfoMeas2_GrayMito_3_00/01/02/03_256_Cells
Texture_InfoMeas2_GrayMito_3_00/01/02/03_256_Cytoplasm
Texture_InfoMeas2_GrayMito_3_00/01/02/03_256_RelateMitoCell
Texture_InfoMeas2_GrayNuclei_3_00/01/02/03_256_Cells
Texture_InfoMeas2_GrayNuclei_3_00/01/02/03_256_FilteredNuclei
Texture_InverseDifferenceMoment_GrayLys_3_00/01/02/03_256_Cells
Texture_InverseDifferenceMoment_GrayLys_3_00/01/02/03_256_Cytoplasm
Texture_InverseDifferenceMoment_GrayLys_3_00/01/02/03_256_RelateLysoCell
Texture_InverseDifferenceMoment_GrayMito_3_00/01/02/03_256_Cells
Texture_InverseDifferenceMoment_GrayMito_3_00/01/02/03_256_Cytoplasm
Texture_InverseDifferenceMoment_GrayMito_3_00/01/02/03_256_RelateMitoCell
Texture_InverseDifferenceMoment_GrayNuclei_3_00/01/02/03_256_Cells
Texture_InverseDifferenceMoment_GrayNuclei_3_00/01/02/03_256_FilteredNuclei
Texture_SumAverage_GrayLys_3_00/01/02/03_256_Cells
Texture_SumAverage_GrayLys_3_00/01/02/03_256_Cytoplasm
Texture_SumAverage_GrayLys_3_00/01/02/03_256_RelateLysoCell
Texture_SumAverage_GrayMito_3_00/01/02/03_256_Cells
Texture_SumAverage_GrayMito_3_00/01/02/03_256_Cytoplasm
Texture_SumAverage_GrayMito_3_00/01/02/03_256_RelateMitoCell
Texture_SumAverage_GrayNuclei_3_00/01/02/03_256_Cells
Texture_SumAverage_GrayNuclei_3_00/01/02/03_256_FilteredNuclei
Texture_SumEntropy_GrayLys_3_00/01/02/03_256_Cells
Texture_SumEntropy_GrayLys_3_00/01/02/03_256_Cytoplasm
Texture_SumEntropy_GrayLys_3_00/01/02/03_256_RelateLysoCell
Texture_SumEntropy_GrayMito_3_00/01/02/03_256_Cells
Texture_SumEntropy_GrayMito_3_00/01/02/03_256_Cytoplasm
Texture_SumEntropy_GrayMito_3_00/01/02/03_256_RelateMitoCell
Texture_SumEntropy_GrayNuclei_3_00/01/02/03_256_Cells
Texture_SumEntropy_GrayNuclei_3_00/01/02/03_256_FilteredNuclei
Texture_SumVariance_GrayLys_3_00/01/02/03_256_Cells
Texture_SumVariance_GrayLys_3_00/01/02/03_256_Cytoplasm
Texture_SumVariance_GrayLys_3_00/01/02/03_256_RelateLysoCell
Texture_SumVariance_GrayMito_3_00/01/02/03_256_Cells
Texture_SumVariance_GrayMito_3_00/01/02/03_256_Cytoplasm
Texture_SumVariance_GrayMito_3_00/01/02/03_256_RelateMitoCell
Texture_SumVariance_GrayNuclei_3_00/01/02/03_256_Cells
Texture_SumVariance_GrayNuclei_3_00/01/02/03_256_FilteredNuclei
Texture_Variance_GrayLys_3_00/01/02/03_256_Cells
Texture_Variance_GrayLys_3_00/01/02/03_256_Cytoplasm
Texture_Variance_GrayLys_3_00/01/02/03_256_RelateLysoCell

Texture_Variance_GrayMito_3_00/01/02/03_256_Cells
Texture_Variance_GrayMito_3_00/01/02/03_256_Cytoplasm
Texture_Variance_GrayMito_3_00/01/02/03_256_RelateMitoCell
Texture_Variance_GrayNuclei_3_00/01/02/03_256_Cells
Texture_Variance_GrayNuclei_3_00/01/02/03_256_FilteredNuclei

Texture_* features have three variations _00_, _01_, _02_, _03_, indicated by "*_00/01/02/03_*".

List S11.2: List of features we retained after full processing:
counts_RelateLysoCell
rp_norm_AreaShape_Area_RelateLysoCell
rp_norm_AreaShape_Area_RelateMitoCell
rp_norm_AreaShape_BoundingBoxArea_FilteredNuclei
rp_norm_AreaShape_BoundingBoxArea_RelateLysoCell
rp_norm_AreaShape_Compactness_Cytoplasm
rp_norm_AreaShape_Compactness_FilteredNuclei
rp_norm_AreaShape_Eccentricity_Cells
rp_norm_AreaShape_Eccentricity_RelateLysoCell
rp_norm_AreaShape_Extent_RelateLysoCell
rp_norm_AreaShape_Extent_RelateMitoCell
rp_norm_AreaShape_FormFactor_RelateMitoCell
rp_norm_AreaShape_MajorAxisLength_Cytoplasm
rp_norm_AreaShape_MaxFeretDiameter_RelateLysoCell
rp_norm_AreaShape_MaximumRadius_RelateLysoCell
rp_norm_AreaShape_MeanRadius_Cells
rp_norm_AreaShape_MinFeretDiameter_Cells
rp_norm_AreaShape_MinFeretDiameter_RelateLysoCell
rp_norm_AreaShape_Solidity_FilteredNuclei
rp_norm_AreaShape_Solidity_RelateLysoCell
rp_norm_Correlation_Correlation_GrayLys_GrayNuclei_Cells
rp_norm_Correlation_Costes_GrayLys_GrayMito_Cells
rp_norm_Correlation_Costes_GrayMito_GrayLys_Cells
rp_norm_Correlation_Costes_GrayNuclei_GrayLys_Cells
rp_norm_Correlation_K_GrayLys_GrayNuclei_Cells
rp_norm_Correlation_K_GrayNuclei_GrayLys_Cells
rp_norm_Correlation_Manders_GrayNuclei_GrayLys_Cells
rp_norm_Correlation_Manders_GrayNuclei_GrayMito_Cells
rp_norm_Correlation_Overlap_GrayLys_GrayMito_Cells
rp_norm_Correlation_Overlap_GrayLys_GrayNuclei_Cells
rp_norm_Correlation_RWC_GrayLys_GrayMito_Cells
rp_norm_Granularity_10_GrayLys_RelateLysoCell
rp_norm_Granularity_12_GrayMito_RelateMitoCell
rp_norm_Granularity_13_GrayMito_RelateMitoCell
rp_norm_Granularity_14_GrayMito_RelateMitoCell
rp_norm_Granularity_15_GrayMito_RelateMitoCell
rp_norm_Granularity_1_GrayLys_RelateLysoCell
rp_norm_Granularity_1_GrayMito_RelateMitoCell
rp_norm_Granularity_2_GrayLys_RelateLysoCell
rp_norm_Granularity_3_GrayLys_RelateLysoCell
rp_norm_Granularity_3_GrayNuclei_FilteredNuclei
rp_norm_Granularity_4_GrayNuclei_FilteredNuclei
rp_norm_Granularity_5_GrayNuclei_FilteredNuclei
rp_norm_Granularity_6_GrayLys_RelateLysoCell
rp_norm_Granularity_6_GrayMito_RelateMitoCell
rp_norm_Granularity_7_GrayLys_RelateLysoCell
rp_norm_Granularity_8_GrayLys_RelateLysoCell

rp_norm_Granularity_9_GrayMito_RelateMitoCell
rp_norm_Intensity_IntegratedIntensityEdge_GrayLys_Cells
rp_norm_Intensity_IntegratedIntensityEdge_GrayLys_RelateLysoCell
rp_norm_Intensity_IntegratedIntensityEdge_GrayNuclei_Cells
rp_norm_Intensity_IntegratedIntensity_GrayNuclei_FilteredNuclei
rp_norm_Intensity_LowerQuartileIntensity_GrayLys_RelateLysoCell
rp_norm_Intensity_MADIntensity_GrayLys_Cells
rp_norm_Intensity_MADIntensity_GrayLys_Cytoplasm
rp_norm_Intensity_MADIntensity_GrayLys_RelateLysoCell
rp_norm_Intensity_MassDisplacement_GrayLys_Cells
rp_norm_Intensity_MassDisplacement_GrayLys_RelateLysoCell
rp_norm_Intensity_MassDisplacement_GrayMito_Cytoplasm
rp_norm_Intensity_MaxIntensityEdge_GrayLys_Cytoplasm
rp_norm_Intensity_MaxIntensityEdge_GrayNuclei_Cells
rp_norm_Intensity_MaxIntensity_GrayLys_Cells
rp_norm_Intensity_MaxIntensity_GrayNuclei_Cells
rp_norm_Intensity_MeanIntensityEdge_GrayLys_Cytoplasm
rp_norm_Intensity_MeanIntensity_GrayLys_Cytoplasm
rp_norm_Intensity_MedianIntensity_GrayLys_Cytoplasm
rp_norm_Intensity_MinIntensityEdge_GrayMito_Cells
rp_norm_Intensity_StdIntensityEdge_GrayLys_Cells
rp_norm_Intensity_StdIntensityEdge_GrayLys_Cytoplasm
rp_norm_Intensity_StdIntensityEdge_GrayNuclei_Cells
rp_norm_Intensity_UpperQuartileIntensity_GrayMito_Cells
rp_norm_Intensity_UpperQuartileIntensity_GrayNuclei_Cells
rp_norm_Mean_PunctaLyso_Distance_Centroid_Cells_Cells
rp_norm_Mean_PunctaLyso_Distance_Centroid_FilteredNuclei_FilteredNuclei
rp_norm_RadialDistribution_FracAtD_GrayLys_2of4_Cytoplasm
rp_norm_RadialDistribution_FracAtD_GrayLys_3of4_Cytoplasm
rp_norm_RadialDistribution_FracAtD_GrayMito_2of4_Cytoplasm
rp_norm_RadialDistribution_FracAtD_GrayMito_3of4_Cytoplasm
rp_norm_RadialDistribution_MeanFrac_GrayLys_2of4_Cytoplasm
rp_norm_RadialDistribution_MeanFrac_GrayLys_3of4_Cytoplasm
rp_norm_RadialDistribution_MeanFrac_GrayLys_4of4_Cytoplasm
rp_norm_RadialDistribution_MeanFrac_GrayMito_4of4_Cytoplasm
rp_norm_RadialDistribution_RadialCV_GrayLys_2of4_Cytoplasm
rp_norm_Texture_AngularSecondMoment_GrayLys_3_00_256_Cells
rp_norm_Texture_AngularSecondMoment_GrayMito_3_00_256_Cytoplasm
rp_norm_Texture_AngularSecondMoment_GrayNuclei_3_00_256_Cells
rp_norm_Texture_Contrast_GrayLys_3_00_256_Cells
rp_norm_Texture_Correlation_GrayLys_3_00_256_Cells
rp_norm_Texture_DifferenceEntropy_GrayNuclei_3_00_256_Cells
rp_norm_Texture_DifferenceEntropy_GrayNuclei_3_00_256_FilteredNuclei
rp_norm_Texture_InfoMeas1_GrayLys_3_00_256_Cytoplasm
rp_norm_Texture_InfoMeas1_GrayMito_3_00_256_Cytoplasm
rp_norm_Texture_InfoMeas2_GrayLys_3_00_256_Cytoplasm
rp_norm_Texture_SumEntropy_GrayNuclei_3_00_256_Cells

*rp_norm_** prefix indicates that the features were standardized.

S12: Removal of similar features

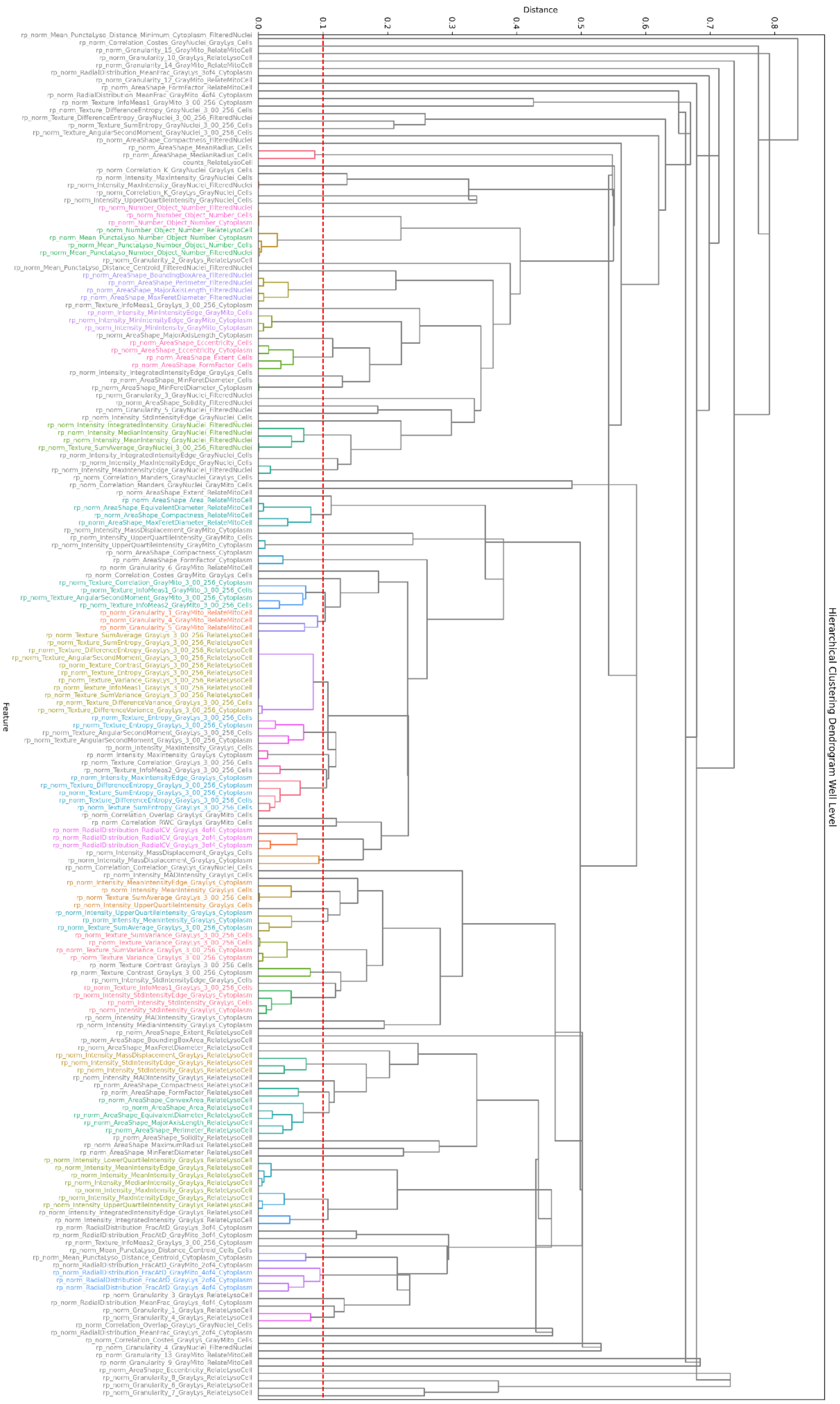

Figure S12: Visual representation of removal of very similar features (Pearson correlation coefficients ≥ 0.9).

Hierarchical clustering with average linkage. Colored feature names represent different highly similar groups, where we only retained one feature, the first in alphabetical order.

Full dataset

A full dataset with full image set, all results, and intermediate files is available in the Zenodo repository - https://doi.org/10.5281/zenodo.17951792 (release v1[5]).

References

(1) Ritz, C.; Baty, F.; Streibig, J. C.; Gerhard, D. Dose-Response Analysis Using R. *PLOS ONE* **2015**, *10* (12), e0146021. https://doi.org/10.1371/journal.pone.0146021.
(2) Wickham, H. *Ggplot2*; Use R!; Springer International Publishing: Cham, 2016. https://doi.org/10.1007/978-3-319-24277-4.
(3) Balk, F.; Hollender, J.; Schirmer, K. Investigating the Bioaccumulation Potential of Anionic Organic Compounds Using a Permanent Rainbow Trout Liver Cell Line. *Environ. Int.* **2023**, *174*, 107798. https://doi.org/10.1016/j.envint.2023.107798.
(4) Pearson, Y. E.; Kremb, S.; Butterfoss, G. L.; Xie, X.; Fahs, H.; Gunsalus, K. C. A Statistical Framework for High-Content Phenotypic Profiling Using Cellular Feature Distributions. *Commun. Biol.* **2022**, *5* (1), 1409. https://doi.org/10.1038/s42003-022-04343-3.
(5) Tome, M.; Janovic Jozef, B. A High-Content Imaging Pipeline to Investigate Subcytotoxic Effects in RTgill-W1 Cells, 2025. https://doi.org/10.5281/zenodo.17951793.
